# Gene flow drives periods of both evolutionary stasis and change in a wild bird population

**DOI:** 10.1371/journal.pbio.3004004

**Published:** 2026-09-21

**Authors:** Simon R. Evans, Henri Bouwmeester, Arie J. van Noordwijk, Marcel E. Visser, Erik Postma

**Affiliations:** 1 Centre for Ecology and Conservation, University of Exeter, Penryn, United Kingdom; 2 Department of Animal Ecology, Netherlands Institute of Ecology, Wageningen, The Netherlands; The Australian National University, AUSTRALIA

## Abstract

The struggle to explain the absence of evolutionary change in heritable, fitness-related traits of free-living populations has become emblematic of our ignorance of the evolutionary process in the wild. Yet while this ‘paradox of stasis’ has been the subject of much discussion, it is primarily identified *a posteriori* and rarely subject to direct quantification. We examined the evolution of clutch size in neighbouring populations of great tits (*Parus major*) before, during and after an experimental evolutionary perturbation. Interannual genetic changes in clutch size were directionally consistent with selection (refuting *absolute* stasis) but were smaller than predicted by adaptive evolutionary models (supporting *relative* stasis). Yet accounting for the contribution of post-selective immigration (i.e., explicitly recognising the distinction between adaptation and evolution) greatly improved our forecast accuracy and this tendency toward overprediction. Indeed, attributing interannual genetic change to its contributing demographic processes revealed immigration to be key to explaining periods of both evolutionary stasis and change, with its evolutionary impact varying across time and space in a manner analogous to the dynamism of natural selection. Our multi-decadal study of clutch size evolution thus shows that recognising the impact of gene flow is crucial to explaining contemporary evolutionary change *and* stasis of a key determinant of life history.

## Abstract 2

Why do we not see evolutionary change in heritable, fitness-related traits of free-living populations? This multi-decade study of the evolution of clutch size in populations of great tits before, during and after an experimental perturbation reveals the importance of gene flow in explaining contemporary evolutionary change and stasis.

In a world characterised by unprecedented rates of physical and biotic environmental change [1–3], a predictive and quantitative understanding of contemporary evolution is vital if we are to anticipate whether, which and how populations will persist, and thus prioritise conservation efforts [4]. Yet while it is widely accepted that microevolutionary change is observable on contemporary timescales, predictions of adaptive evolution that combine the strength of selection and the heritable transmission of trait variation [e.g., 5–7] appear to perform poorly when applied to free-living populations. Evolutionary ecologists have struggled to resolve this problem for over three decades [8,9]. Even as analytical advances have enabled a transition from predicting responses at the phenotypic level [10–13] to quantifying responses at a genetic level [14–16], the ‘paradox of stasis’ has withstood numerous attempts at resolution and become emblematic of our struggle to understand contemporary adaptive evolution in natural populations. Yet while it is the subject of much resolutory discussion [9,17–25], the paradox of stasis is seldom subject to objective assessment; rather, it is generally invoked *a posteriori*, which risks exaggeration of its prevalence through observation selection bias.

A potential explanation that is often overlooked is that adaptation—a population’s *in situ* response to selection (i.e., the response if the population was a closed system)—is not the only process contributing to local evolutionary change. Immigrants are likely to be genetically distinct from their host population, such that gene flow will also shape a population’s genetic composition. Indeed, even very occasional arrivals can be highly influential on the host population’s evolutionary trajectory [26,27]. Furthermore, immigration rates often covary with local mortality [28–30], highlighting possible interdependencies of the contributions of local adaptation and gene flow. While debate over the relative importance of adaptive versus nonadaptive processes in shaping evolution of fitness and life history traits has a lengthy history in evolutionary biology [31–33], quantitative genetic studies of trait evolution rarely consider the relative contributions of both gene flow and adaptation.

As a fundamental measure of an individual’s reproductive rate, the evolutionary dynamics of clutch size have been studied in detail [34–38], including its failure to evolve as expected based on observed selection [19,35,39–42]. We here use long-term observations of clutch size in a nestbox-breeding great tit (*Parus major*) population on the Dutch Wadden Sea island of Vlieland to disentangle and quantify the demographic drivers of evolutionary change. Within the island, there is a clear spatial distinction with respect to clutch size, with nests in the smaller wood plots of the West (Fig 1) historically having larger clutches than those of the East [43], and both genotyping [44] and quantitative genetic modelling [43] have shown that the western and eastern breeding populations within the island are genetically distinct. In the late 1990s, an 8-year artificial selection experiment reversed the genetic difference in clutch size [39], after which we continuously and comprehensively monitored the West and East breeding populations for a further two decades to quantify natural selection on clutch size in both populations and to track ongoing evolution of reproductive rate. Using these data for two neighbouring breeding populations, we here quantify annual selection on clutch size, its genetic change across and within a 44-year timespan (1980–2023) that incorporates the periods preceding and following the selection experiment, and quantify and compare the contributions of local adaptive processes and immigration to interannual genetic change within each.

**Fig 1:**
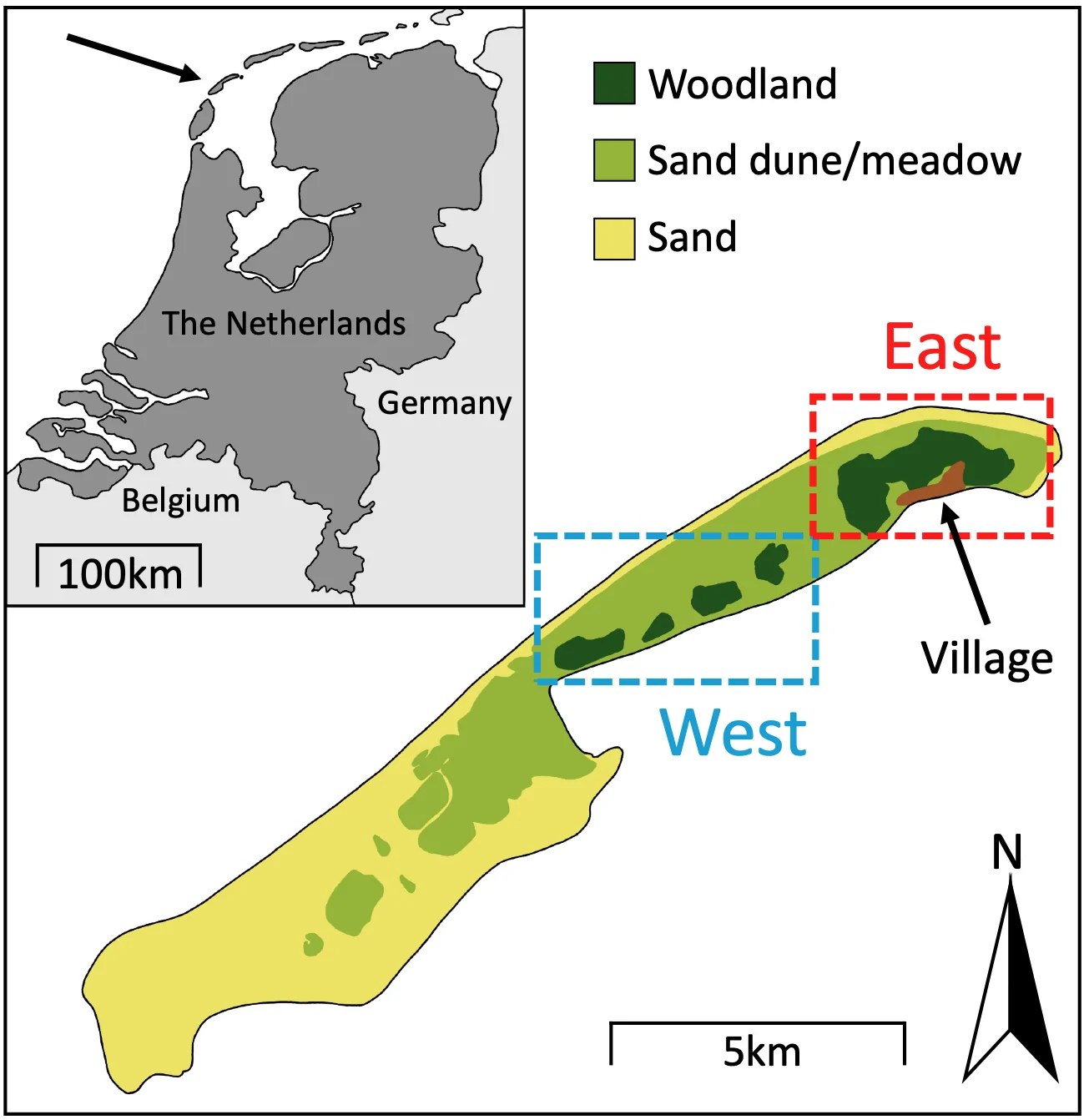
Map showing the five distinct woodlands on the Dutch island of Vlieland and their attribution to either West or East, and the location of Vlieland within the Netherlands (*inset*). Also shown is the island’s only village, Oost Vlieland (shown in brown), within which citizens have installed their own nestboxes. Total woodland cover is ~9% of the island’s area [45]. Illustration by Amy Berry based on Google Earth data.

## Results

Prior to the artificial selection experiment (pre-selection period: 1980−1995), mean clutch size in the West was more than one egg larger than in the East (mean annual W-E difference: 1.1 eggs; Fig 2a). Estimated breeding values from a quantitative genetic ‘animal model’ (S1 Table) indicate a genetic W–E difference of 0.13 [0.01, 0.25] eggs (posterior mean [95% highest probability density interval]), consistent with the phenotypic difference in clutch size observed among birds of different origin within each breeding site (Fig S1) [43]. As described previously [39], an 8-year regime of strong and spatially divergent artificial selection on clutch size (experimental period: 1996−2003; Fig 2b; S2 Table) drove a net evolutionary change of −0.36 [−0.62, −0.10] eggs to the West-East clutch size difference, such that the spatial genetic difference was reversed (Fig 2C; S3 Table).

**Fig 2:**
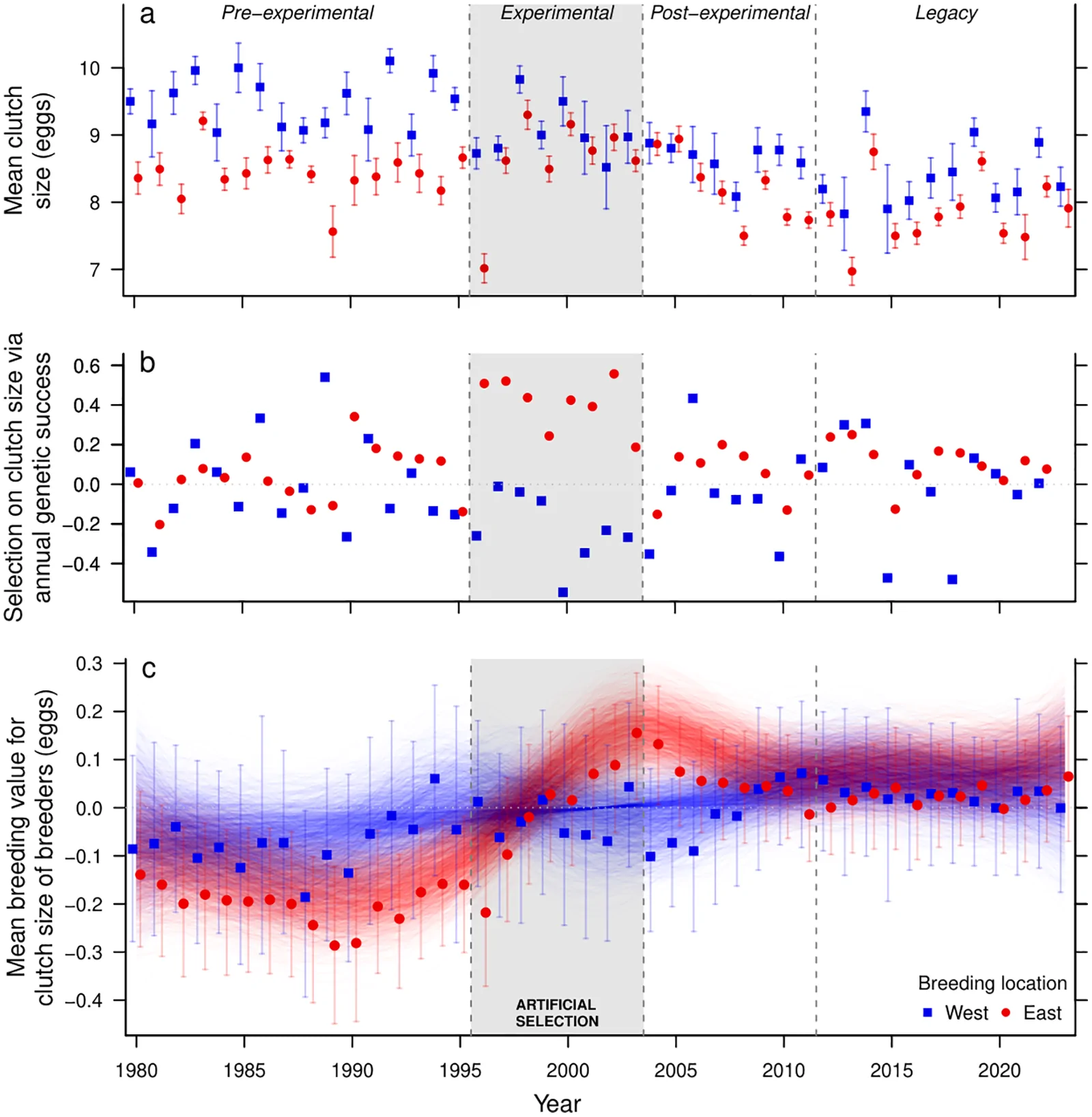
Annual phenotypic expression, selection and breeding value of clutch size in the West and East breeding populations of great tits on Vlieland. (a) Annual mean great tit clutch sizes in West and East between 1980 and 2023. **(b)** Selection differentials detailing annual covariance of clutch size with annual genetic success. **(c)** Posterior annual mean breeding values for clutch size of known breeders over the same period. We divide the 44-year study into four periods, defined by the length of the artificial selection experiment: pre-experimental (1980–1995), experimental (1996–2003), post-experimental (2004–2011) and legacy (2012–2023). In (a), error bars extend one standard error either side of the mean; for (b) there is no sampling error (all breeders are sampled); in (c) vertical bars span the 95% highest probability density interval. For each sampled iteration, best-fit lines from general additive models of mean annual breeding value for clutch size against year are plotted to aid visualisation of the evolutionary change of each breeding population across the study period. Note that for all three plots, the horizontal (i.e., temporal) positions of point estimates are consistently offset according to breeding location. The data and code needed to generate this Figure are archived in the Zenodo online repository [92].

This genetic distinction between West and East was lost during the 8 years following the artificial selection experiment (post-experimental period: 2004−2011; Fig 2c), when natural selection operated without experimental interference (Fig 2b). While at face value this initial evolutionary return toward the pre-experimental state is consistent with the adaptationist expectation that the experimental evolutionary perturbation (which was driven by strong directional artificial selection) is opposed by natural selection, annual selection on clutch size during the post-experimental period was slightly favouring smaller clutches in the West (mean annual selection differential: −0.048) and larger clutches in the East (0.051), and neither was significantly different from zero (one-sample *t*-tests: West: $t_{\text{7}}=-\text{0}.\text{5}\text{3},\;P=\text{0}.\text{6}\text{1}\text{2}$, East: $t_{\text{7}}=\text{1}.\text{1}\text{2},\;P=\text{0}.\text{2}\text{9}\text{9}$). Furthermore, directional selection during the post-experimental period was statistically indistinguishable from the directional selection during the pre-experimental period in the West (mean: 0.005) and the East (0.037) (Welch two-sample *t*-tests comparing pre-experimental and post-experimental periods: West: $t_{\text{1}\text{2}.\text{9}}=\text{0}.\text{4}\text{9},\;P=\text{0}.\text{6}\text{3}\text{2}$, East: $t_{\text{1}\text{5}.\text{3}}=-\text{0}.\text{2}\text{4},\;P=\text{0}.\text{8}\text{1}\text{3}$; Fig 2b; S2 Table), which was characterised by evolutionary stasis of clutch size in both West and East (Fig 2c; S3 Table). This rapid and sustained genetic change in the absence of strong natural selection during the post-experimental period is the opposite of the situation of little or no adaptive evolution despite strong selection described by the paradox of stasis, and might therefore be argued to represent a corresponding ‘paradox of change’. To better determine the explicability of contemporary clutch size evolution—including evolutionary stasis—in our populations, we examined the interannual genetic changes of both breeding populations in greater detail and across a multi-decadal observation period that spans the evolutionary perturbation experiment. Doing so offers an objective assessment of the extent to which genetic change corresponds with expectations based on phenotypic observations.

In a first step, we estimated the phenotypic covariance between clutch size and annual fitness. Commonly referred to as the selection differential *S*, interannual variation in *S* captures both fluctuating selection and the effect of drift, and outside of the experimental years there is no evidence that variation in directional selection among years and/or subpopulations exceeds what is expected under drift alone (*χ*^2^ = 0.90; *P* = 0.49). However, our sampling of the complete breeding populations means that, irrespective of the underlying mechanism underlying variation in *S*, it is expected to explain interannual genetic change in the breeding population (i.e., $\Delta \overset{―}{a}$). In practice, however, we found it to be a highly unreliable predictor of $\Delta \overset{―}{a}$ ($r_{\text{7}\text{0}}=\text{0}.\text{0}\text{8}\;[-\text{0}.\text{2}\text{0},\;\text{0}.\text{3}\text{1}]$), and their relative proportion (i.e., $\Delta \overset{―}{a}$/*S;* quantifiable as the slope of a regression of $\Delta \overset{―}{a}$ against *S* forced through the origin) was close to zero ($\frac{\Delta \overset{―}{a}}{S}=\text{0}.\text{0}\text{3}\;[-\text{0}.\text{0}\text{7},\text{0}.\text{1}\text{4}]$; $P\left(\frac{\Delta \overset{―}{a}}{S}>\text{0}\right)=\text{0}.\text{7}\text{3}\text{3}$; Fig 3a). In other words, on average, 3% of the selection differential was translated into genetic change. To test if this ratio differs from expectation, we generated a quantitative expectation of the expected efficiency of natural selection (in terms of generating genetic change; i.e., our null expectation) using year- and subpopulation-specific heritabilities ($h_{within-year}^{\text{2}}$), halved to account for sex-limited trait expression [46] (S2 Fig). This yields a mean expected conversion rate of selection into genetic change of 0.11 [0.08, 0.13]. While estimated with large uncertainty, the ratio of the realised efficiency of natural selection ($\Delta \overset{―}{a}/S$) to that expected ($\frac{\text{1}}{\text{2}}h_{within-year}^{\text{2}}$) is 0.31 [−0.64, 1.36] and most likely less than unity ($P(\frac{\Delta \overset{―}{a}/S}{\frac{\text{1}}{\text{2}}h_{within-year}^{\text{2}}})<\text{1}=\text{0}.\text{9}\text{1}\text{4}$).

**Fig 3:**
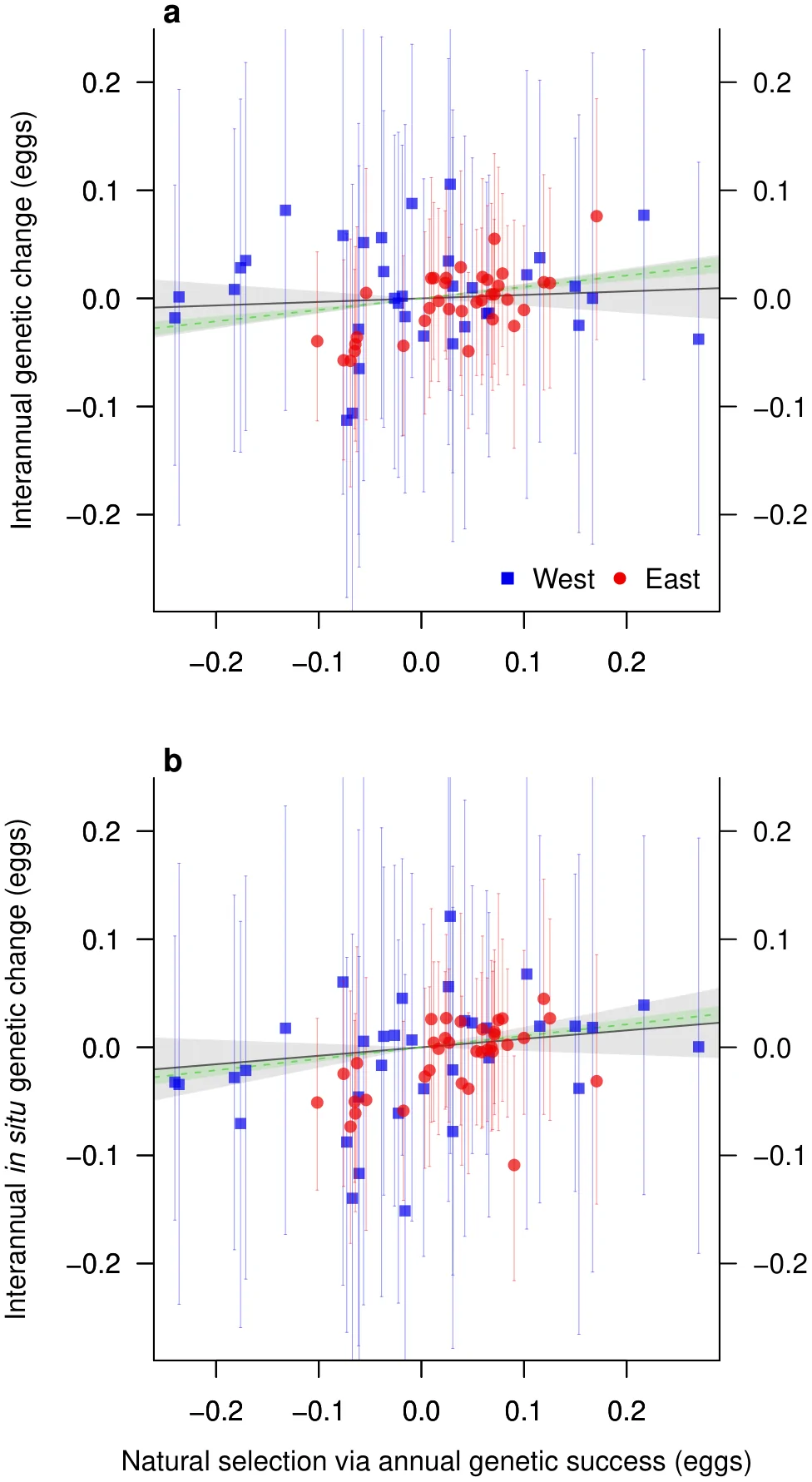
Association between natural selection on clutch size and interannual genetic change, according to whether newly recruited immigrants are (*a*) included or (*b*) excluded. The location of points is defined by the posterior mean, with 95% highest probability density intervals plotted as vertical bars. The posterior mean for the regression (forced through the origin) of natural selection on clutch size (via annual genetic success) against the inferred interannual genetic change is plotted as a solid, grey line with the shaded grey area spanning the 95% highest probability density interval for the slope, which can be interpreted as the half the ‘realised’ heritability [47]. Years when the breeding populations were subjected to artificial selection on clutch size (1996-2003) are excluded. The data and code needed to generate this Figure are archived in the Zenodo online repository [92].

Our finding that interannual genetic change is poorly predicted by estimates of selection might be interpreted as evidence that clutch size is subject to the paradox of stasis in our population, for example due to selection differentials being environmentally biased or the noise produced by stochastic processes overwhelming the signal of adaption. Unless populations are truly closed, however, examining change at the level of the breeding population fails to account for the evolutionary contribution of immigrants. Indeed, when we quantify interannual genetic change as the *in situ* population response ($\Delta {\overset{―}{a}}_{in\;situ}$; i.e., excluding immigrants), we find that predictive reliability increases ($r_{\text{7}\text{0}}=\text{0}.\text{1}\text{9}\;[-\text{0}.\text{0}\text{5},\;\text{0}.\text{4}\text{1}]$) and the apparent response to selection is much increased (slope of a regression forced through the origin, $\Delta {\overset{―}{a}}_{in\;situ}/S=\text{0}.\text{0}\text{8}\;[-\text{0}.\text{0}\text{3},\;\text{0}.\text{1}\text{9}]$; $P\left(\Delta {\overset{―}{a}}_{in\;situ}/S>\text{0}\right)=\;\text{0}.\text{9}\text{1}\text{5}$; Fig 3b). Furthermore, support for a paradox of stasis appears to be reduced ($\frac{\Delta {\overset{―}{a}}_{in\;situ}/S}{\frac{\text{1}}{\text{2}}h_{within-year}^{\text{2}}}=\text{0}.\text{7}\text{4}\;[-\text{0}.\text{3}\text{4},\;\text{1}.\text{7}\text{7}]$; $P(\frac{\Delta {\overset{―}{a}}_{in\;situ}/S}{\frac{\text{1}}{\text{2}}h_{within-year}^{\text{2}}})<\text{1}=\text{0}.\text{6}\text{8}\text{4}$), although the estimates are too imprecise to lend strong statistical support ($P(\Delta {\overset{―}{a}}_{in\;situ}>\Delta \overset{―}{a})$ = 0.885). Empirical studies supporting a ‘paradox of stasis’ rarely discount the contribution of immigrants when comparing predicted evolutionary change and inferred response, let alone quantify the evolutionary impact of immigration and compare its impact to that of the *in situ* genetic change, which depends on the scale of *in situ* population turnover relative to the frequency and the genetic distinctiveness of immigrants (Fig 4).

**Fig 4:**
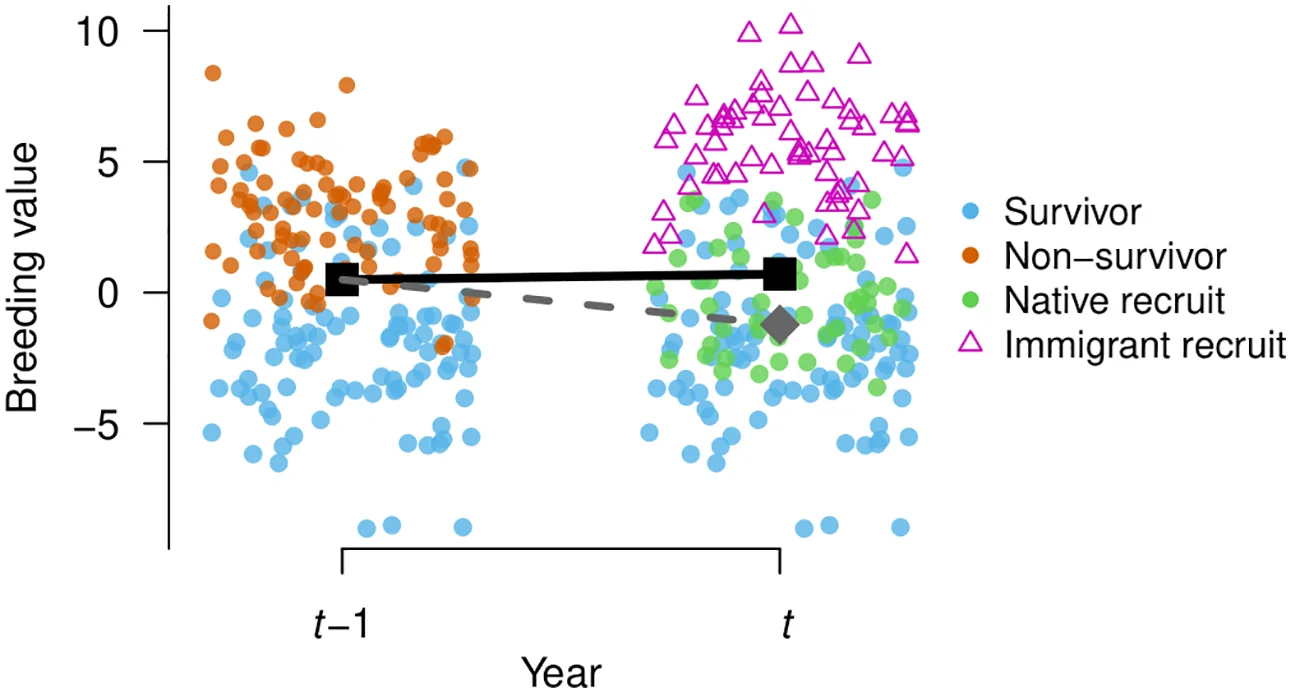
Graphical representation of the distinction between local adaptation and overall genetic change. The breeding values of all individuals in a simulated breeding population are plotted. Individuals plotted in blue bred in both years, those plotted in red did not survive to breed in year *t*, while those plotted in green were new recruits to the breeding population in year *t*. The genetic change attributable to local adaptation (i.e., the net effect of breeder mortality and the recruitment of local-origin individuals) is represented by the grey dashed line, which differs from the total genetic change in the breeding population (solid black line) that additionally includes the contribution of immigrant recruitment. The data and code needed to replicate this Figure are archived in the Zenodo online repository [92].

Can immigration explain how evolutionary trajectories can differ so markedly between time periods characterised by very similar selection regimes (Fig 2)? To quantify the distinct contributions of selection versus immigration, and to elucidate their relative evolutionary importance, we applied a demographic balancing equation approach by decomposing interannual genetic changes among the three demographic processes contributing to turnover within the breeding populations: (*i*) breeder mortality, (*ii*) recruitment of local-origin fledglings, and (*iii*) recruitment of immigrants (where local and immigrant recruitment categories are both defined at the scale of each breeding population). Alongside this switch from a focus on explicitly *evolutionary* processes to the *genetic impact* of specific *demographic* processes, readers should note that while local adaptation is achieved via (*i*) and (*ii*), and the immediate effect of gene flow is quantified by (*iii*), all three are subject to stochasticity (i.e., influenced by genetic drift [48]).

To verify the performance of this approach, we first consider the experimental period. The artificial selection experiment was designed to act via selective recruitment of local-origin fledglings. In the West, the artificial selection for smaller clutches should thus manifest as a negative contribution of native (i.e., local origin) recruitment to the mean breeding value for clutch size following experimental years, while for the East, where artificial selection favoured larger clutches, we expect native recruitment to increase the mean breeding value for clutch size. Our best estimates of the annual genetic change attributable to recruitment of local-origin individuals in the experimental period are consistent with these expectations (West: −0.07 [−0.14, −0.01] eggs/yr; East: 0.03 [−0.00, 0.05] eggs/yr; S3 Fig; S4 Table), with posterior probabilities that our expectations were met of 0.991 and 0.964 for West and East, respectively.

We turn now to the genetic effects of immigrant recruitment during the artificial selection experiment. As the experiment proceeded, the genetic difference in clutch size between West and East was first lost (as the response to artificial selection drives each breeding population’s mean breeding value for clutch size toward the whole-island mean), and then reversed (as the two breeding population’s mean breeding values continue on their divergent evolutionary trajectories: Fig 2c). For each breeding population, we can thus expect the genetic impact of immigrants to change across the experimental period in the opposite direction to the genetic change of the breeding population to which they are recruiting (i.e., to become more positive in the West and more negative in the East). This is because immigrants will inevitably have originated in a population experiencing a very different selection regime, be that artificial [if emigrating from the other nestbox-breeding population, e.g., from West to East] or natural [if originating outside the nestbox study]. We found strong support for this in the East (−0.02 [−0.03, −0.01] eggs/yr^2^) but not the West (0.00 [−0.04, 0.04] eggs/yr^2^), with the posterior probabilities that the direction of inferred change is consistent with our prediction being 0.995 and 0.447 for East and West, respectively. This provides a first indication of the dynamic impact of immigration on evolutionary trajectories of typical study populations.

By summing the genetic impact of native recruitment and the loss of established breeders due to mortality, we can quantify the net impact of these *in situ* processes on the populations’ evolutionary trajectory. This describes the genetic impact of local adaptation, which can be compared to the contribution made to genetic change by immigrant recruitment. Beyond the exceptional circumstances of the experimental period (when the genetic impact of native recruitment in both breeding populations was deliberately manipulated), demographic decomposition shows immigration to strongly influence clutch size evolution (Fig 5; S4 Table). Indeed, the post-experimental evolutionary trajectory of clutch size would have been very different without the effects of immigration: near-static in the East (−0.00 [−0.03, 0.02] eggs/ yr^2^) and proceeding in the opposite direction in the West (−0.03 [−0.11, 0.04] eggs/ yr^2^), the latter unexpectedly continuing the evolutionary reduction of clutch size that occurred during the experimental period. However, the breeding population of the West is much smaller than that of the East (S1 Table), so will also be more susceptible to genetic drift [48], which contributes to the observed covariances of trait expression and annual genetic success. Demographic decomposition also demonstrates that evolutionary stasis occurs not only during periods of evolutionary inactivity (e.g., for the legacy period in the West, when each of the three demographic processes has negligible genetic impact on clutch size: S3 Fig, S4 Table), but also during periods of dynamic equilibrium, as seen in the East during the pre-experimental period, when *in situ* evolution of smaller clutches is countered by the positive impact of immigration (Figs 5, S3, S4 Table). Indeed, these data suggest that although on average clutch size appears to be selectively neutral in the East during the 16-year pre-experimental period (Fig 2b, S2 Table), this conceals negative selection on clutch size at a genetic level, as could be driven by selection on traits genetically correlated with clutch size.

**Fig 5:**
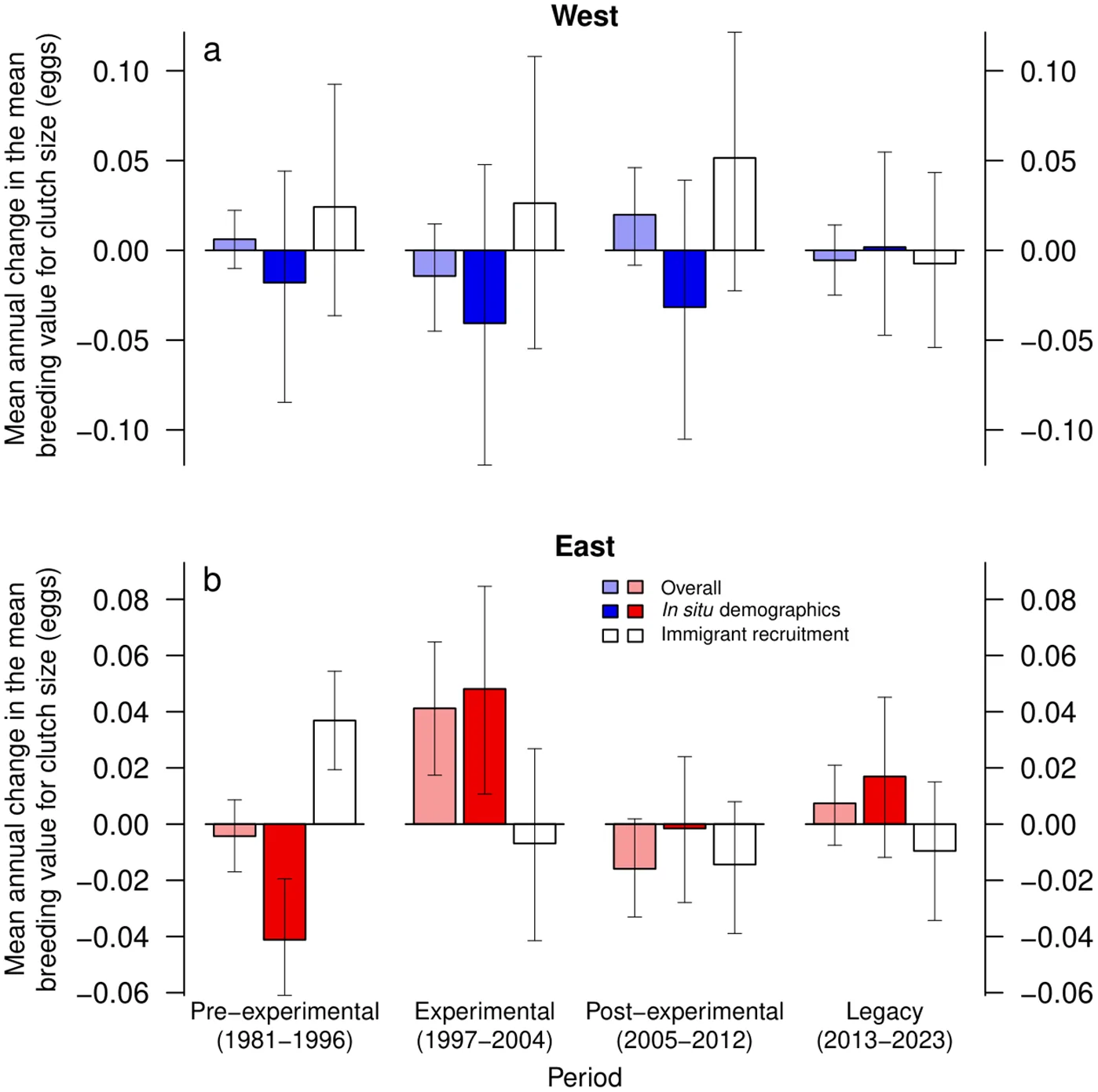
Mean interannual changes in the estimated mean breeding value for clutch size of breeders in (a) the West and (b) the East for each of four time periods, and their demographic decomposition into the effects of in situ population turnover (the sum effect of breeder mortality and recruitment of local-origin individuals to the breeding populations: S2 Fig) and immigrant recruitment. Bar heights represent the posterior mean from our animal model of clutch size, error bars span the 95% highest probability density interval. The data and code needed to generate this Figure are archived in the Zenodo online repository [92].

Having accounted for the inferred genetic changes to clutch size in both breeding populations, there remains a further unresolved puzzle: despite the pace at which the experimentally-induced genetic difference in clutch size was lost (S3 Table), the pre-experimental distinction between the two breeding populations had still not been restored two decades later (Fig 2c). At least in part, this is attributable to a diminishing demographic distinction between West and East, with respect to both the rate of immigration from the mainland and the fate of these mainland immigrants’ descendants. Between 1980 and 2023, the frequency of unknown-origin recruits doubled in the East yet declined in the West (S4 Fig). The survival selection against the descendants of immigrants that was a feature of the pre-experimental period in the East (but not the West) had also ceased by the legacy period (Fig 6c). While the mean breeding value of unknown-origin immigrants has not changed across the study period (slope of linear regression on year: West: 0.001 [−0.003, 0.005] eggs/yr, East: 0.000 [−0.004, 0.004] eggs/yr; S5 Fig), changes to both the origin of immigrant cohorts and gene flow mean that the breeding populations of West and East are much more demographically and genealogically alike now than they were nearly half a century earlier (Figs 6a, S4). Although the environmental drivers for this remain to be identified, recent decades have seen a shift in the composition of Dutch forests [49], including on Wadden Sea islands such as Vlieland [50], with coniferous monoculture giving way to mixed deciduous woodland, possibly making it more similar to the habitat experienced by mainland birds, from which most unknown-origin immigrants descend [44]. Yet whatever the driving forces responsible, a key conclusion is that this multi-decadal process of demographic and genealogical homogenisation is not explicable if considering only the genetic differences between source populations, which have remained static across a near-half-centurial study period.

**Fig 6:**
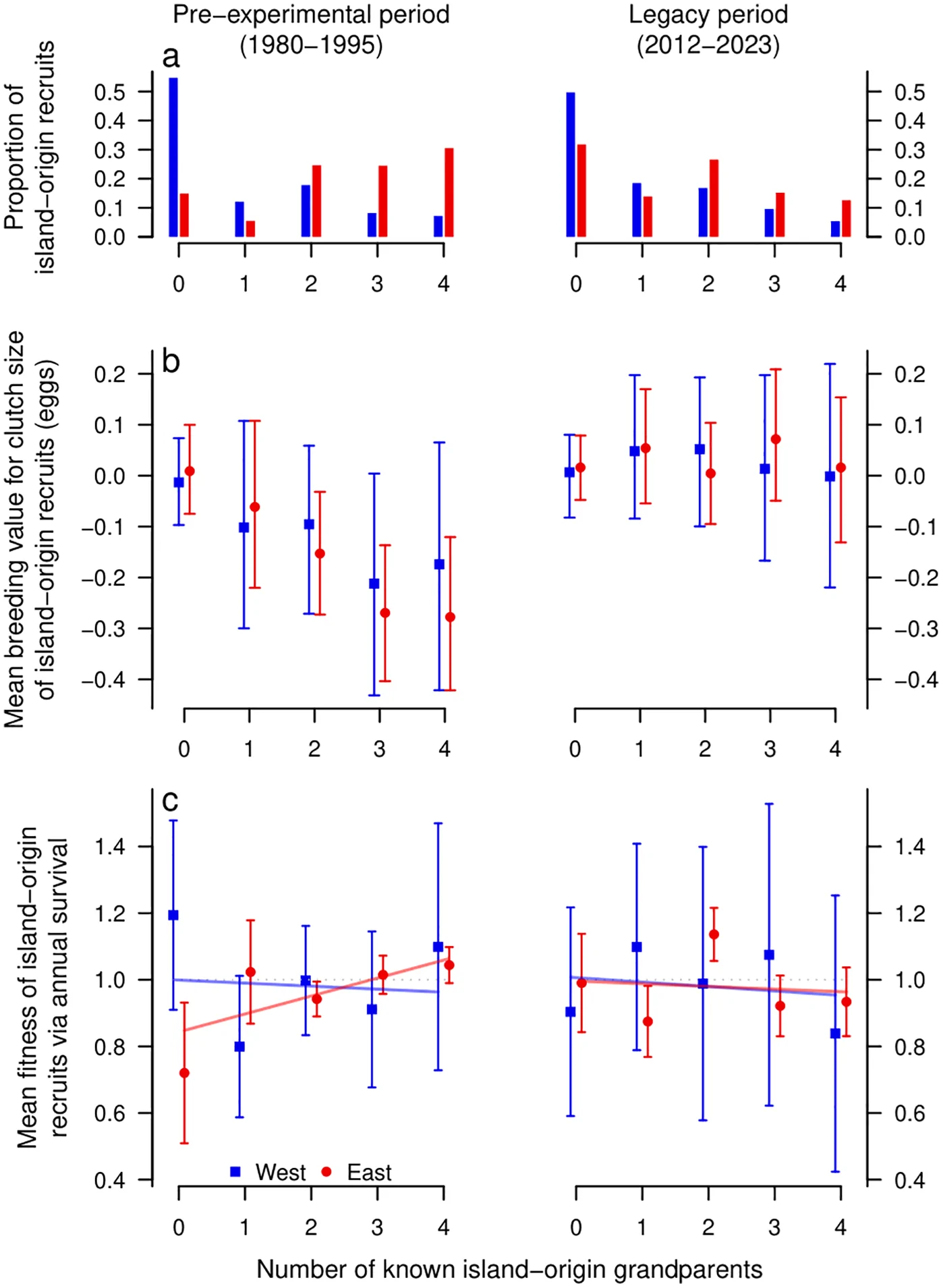
Demographic frequency, mean breeding value for clutch size, and fitness via interannual survival of Vlieland-origin great tits breeding in West and East parts of the island (see Fig 1) during the pre-experimental (*left*) and legacy periods (*right*), according to their immigrant ancestry status. **(a)** Mean annual proportion. **(b)** Breeding value for clutch size (mean and 95% HPDI). **(c)** Mean fitness via interannual survival, with the best-fits from site- and period-specific linear regressions plotted. Since immigration status is not sex-limited, males are included. The data and code needed to generate this Figure are archived in the Zenodo online repository [92].

## Discussion

While responses to observed selection on clutch size were directionally consistent with adaptationist expectations, they were, overall, smaller than predicted. Even so, observed selection could not account for sustained periods of evolutionary flux and stasis. On this basis, we might be tempted to consider our results not only supportive of the ‘paradox of stasis’ but also indicative of a ‘paradox of change’. However, attributing contemporary evolution to its demographic sources shows immigration to be a major influence, such that contemporary life history evolution is explicable only by accounting for the movement of individuals into the population. That the impact of immigration frequently exceeded that of local adaptation in shaping contemporary trait evolution in the wild—even in the aftermath of the trait’s deliberate evolutionary perturbation—demonstrates that predictions of how populations will evolve in response to environmental change must consider the impact of gene flow, with consequences for the plausibility of, e.g., assisted evolution [51].

The evolutionary impact of immigration will depend not only on the immigration rate, but also on the genetic distinction between natives and immigrants [31,45,52]. We here present a novel analytical framework that establishes the evolutionary contribution of each demographic process. By applying this demographic decomposition to the evolution of clutch size in two, neighbouring breeding populations of great tits, we see that periods of evolutionary stasis occur not only in the absence of evolutionary influential demographic change (as seen in the West during the legacy period) but also when all three demographic processes are impactful (as in the East during the pre-experimental period, when immigration counteracted the evolutionary decline in clutch size that would otherwise have occurred). Similarly, the genetic trends in clutch size following cessation of the artificial selection regime are explicable only in light of the impact of immigration. Ignoring the impact of immigration when testing the predictability of local adaptation (i.e., conflating adaptation and evolution) inevitably hampers the apparent fidelity of a predictive evolutionary framework that is proven in other contexts [53].

While insular systems such as ours may seem atypical, they have been at the forefront of efforts to understand why evolutionary forecasts perform so poorly when applied to free-living populations [10,39], based largely on their relatively closed nature, which allows local adaptation to be better quantified. In avian study populations more broadly, immigrants of unknown origin usually account for the majority of recruits [42,54]. Nevertheless, a recent poll of evolutionary ecologists ranked gene flow as the least important of the candidate factors limiting evolutionary responses to climate change [33]. However, as we show, (*i*) relative isolation does not nullify the evolutionary impact of immigration on traits’ evolutionary trajectories [26,27], (*ii*) at evolutionary stasis—the apparent commonality of which is so problematic to evolutionary ecologists [9,17]—the impact of immigration must balance the sum effect of in situ adaptive processes, and (*iii*) immigration and gene flow are both shaped by environmental factors [29,55], and hence are likely to vary over time in an evolutionarily influential way in response to local or global environmental change. Thus, the evolutionary impact of immigration and gene flow—which should not be assumed to be constant through time and space—must be accommodated into evolutionary predictions if we are to achieve the reliable forecasting framework that would allow us to predict evolutionary change with confidence.

## Materials and methods

### Ethics statement

The research was carried out with permission of the State Forestry Service, and under licence AVD801002017831 (and earlier versions of this permit) of the Dutch Centrale Commissie Dierexperimenten (CCD).

### Study system

The breeding attempts of great tits on the Dutch island of Vlieland (53.17°N, 5.03°E) have been monitored every year since 1955, when nestboxes were first installed. Since then, additional nestboxes have been installed as necessary to ensure that availability exceeds demand. When first captured (as nestlings or breeding adults), all individuals are marked with a uniquely numbered aluminium leg-ring, and adults with an additional unique combination of coloured, plastic leg-rings. From the start of each breeding season, all nestboxes are visited regularly to measure clutch size (the number of eggs in the completed clutch) and record parent identities. While some females produce multiple clutches per year, we here consider each female’s first observed clutch of the season to represent her clutch size phenotype for that year.

Extensive egg and nestling removals were carried out in 1960–63 and 1967–68, reducing annual fledgling production by ~60% [56,57]. In 1970–74, there followed experimental removal of adults after the natural end of their first breeding attempt of the season [58]. Owing to these interventions, we focus on the period from 1980 to 2023, giving a 16-year ‘pre-experimental period’ (1980–1995), an 8-year ‘experimental period’ (1996–2003) and a 20-year period after the experiment’s end. After initial results indicated that some degree of evolutionary recovery of the pre-experimental status quo had occurred, we divided this latter period in two: an initial ‘post-experimental period’ (2004–2011) of equal length as the experiment, and a 12-year ‘legacy period’ (2012–2023). Since first clutch sizes were unaffected by the earlier removal experiments [57,58], breeding records for 1955–79 were included in the animal model, and contributed to construction of the population pedigree it relies on. Experiments manipulating the workload of male breeders were carried out 2011–2014 but did not influence the male’s own survival, nor the survival and later reproductive success of his mate, nor the recruitment success of their offspring [59]. Thus, this experiment does not appear to have influenced selection on clutch size so we did not exclude the nests of experimental or control individuals.

### Artificial selection experiment

Breeding attempts in East and West were subject to contrasting regimes of artificial selection on clutch size throughout the 8 years from 1996 to 2003, either favouring smaller clutches (in the West) or larger clutches (in the East). In brief, mean clutch sizes of East and West were calculated once most of the first breeding attempts had reached the incubation stage (such that clutch size was observed for the majority of breeding females) and used as selection thresholds in an experimental regime that aimed to reduce or reverse the established distinction between East and West. For nests in the East, artificial selection thus favoured larger-than-average clutches: females laying a small first clutch had all eggs removed from this and any replacement clutches, such that none of their offspring fledged that year. Conversely, in the West, females with a first clutch larger than that year’s mean had all their eggs removed. For a detailed description of the selection experiment, see [39].

### Quantifying individual breeding values and trait evolution

We used an animal model [60,61] to attribute phenotypic variation in clutch size to additive genetic (i.e., heritable) and environmental components. The animal model is a form of linear mixed-effects model in which a familial relatedness pedigree is used to infer the extent to which heritable factors account for the observed phenotypic resemblance of relatives. We included all clutch size observations from 1955–2023, including 890 first clutch size observations from nestboxes in the gardens of the island’s only town. A single observation from a nestbox sited elsewhere on the island was excluded. The informative pedigree (i.e., the minimal pedigree that includes all known familial relationships between observed breeders [both sexes]) included 8,793 individuals, of which 2,706 are founders, with a maximum pedigree depth of 52 (6,085 maternities and paternities, mean maternal sibship size of 2.3, mean paternal sibship size of 2.2). Age-class (1 year-old versus older [62]) and woodlot identity (which of six nestbox areas the nest is located within; Fig 1) were included as categorical fixed effects.

To control for the potential influence of environmentally-driven trends in clutch size across each focal period, we fitted period-and-woodlot-specific temporal trends as fixed effects (modelling temporal trends for the pre-experimental (1980–1995), experimental (1996–2003), post-experimental (2004–2011) and legacy (2012–2023) periods, as well as a fifth timespan (1955–1979) that preceded our study period but for which clutch size observations were included in our animal model. We estimated the specific contributions of additive genetic, permanent environment and year-level temporal change to the phenotypic variance in clutch size (conditional on the specified fixed effects [63]). We also included maternal identity as a random environmental effect to limit the potential for shared early-life environmental effects to upwardly bias the estimated additive genetic effect on clutch size. For every female with an unknown dam (*n* = 1,218), we created a unique dummy maternal identity.

As they are expectations, breeding values are unobservable and thus their values are predicted with some uncertainty [64,65]. A Bayesian implementation of the animal model relying on Markov chain Monte Carlo methods allows this uncertainty to be propagated to downstream statistical computation [65]: quantitative genetic statistics are calculated for each sampled iteration and the distributions across all sampled iterations taken to represent posterior distributions. We used the MCMCglmm package (v.2.34) [66], specifying a weakly informative parameter-expanded prior that closely approximates the scaled noncentral *F*-distribution with numerator and denominator degrees of freedom equal to one and a scale parameter of 1,000 [66,67]. A model with inverse-Wishart priors specifying a degree-of-belief parameter of 0.002 gave quantitatively similar results (S5 Table). After a burn-in period of 20,000 iterations, we retained 2,000 samples with which to construct posterior distributions. A thinning interval of 1,000 iterations yielded effective sample sizes exceeding 1,700 and correlations among successively sampled iterations of <0.01 for all random effects. We use posterior means as point estimates and provide the 95% highest probability density interval alongside these.

Within each of the four periods, the pace of evolutionary change in clutch size was estimated using linear regressions of the annual mean breeding value of known breeders on year (as a numerical variable) for each sampled iteration; the distribution of regression coefficients was taken to represent the posterior distribution of the rate of evolutionary change [65]. We used neutral sampling simulations to generate the posterior probability that the degree of evolutionary change can be explained by drift alone [65].

### Assessing predictions of genetic change

Since we focus on a trait that is expressed on an annual cycle, we approximate the relevant target of selection as ‘annual genetic success’: a measure of a female’s genetic contribution to the following year’s breeding population via both her own survival and the recruitment of offspring produced in the focal breeding year (including from any later nests). Annual genetic success was calculated as the sum of (*i*) a binary representation of her own survival (i.e., 0 or 1) and (*ii*) half the number of her offspring that successfully recruit [68–70]. Since the sampled breeders define our focal population, sampling uncertainty does not contribute uncertainty to our selection estimates [71].

Reliance on this measure of success allows us to quantify the combined effect of viability and fecundity selection, and genetically influential changes on an annual scale will compound to create the intergenerational genetic change that is evolution *sensu* *stricto* [72]. However, it should be recognised that the selection interval is subgenerational: if they survive to the following breeding season, individuals contribute to their own annual genetic success score. The reproductive component of the score is also flawed, being a ‘mixed fitness’ measure (i.e., combining the fecundity of females and early-life survival of their offspring [73]).

In the period prior to the selection experiment, second-order year- and site-specific polynomial models of annual fitness on clutch size find statistical support (at an α-level of 0.05) for nonlinear (i.e., quadratic) phenotypic selection differentials on clutch size for three of the 16 years in the West (one negative, two positive), and for 1 year (positive) in the East. Regarding the directionality of quadratic selection coefficients across all years, regardless of statistical significance, negative values occurred more frequently in both West (10 versus 6 years) and East (9 versus 7 years), but for neither patch is this a significant departure from expectations when a coefficient of either sign is equally likely, as described by a one-tailed binomial test: (West: *P* = 0.455; East: *P* = 0.804). Given that nonlinear selection on clutch size appears from these analyses to be weak, we focus on directional selection hereafter, defining it as the covariance between clutch size and the relative (to the year- and site-specific mean) annual genetic success.

We acknowledge that drift will cause annual selection differentials to vary among years in the absence of interannual variation in the strength or direction of selection [74]. Because we sample the complete population, the role of estimation error is negligible and heterogeneity in selection differentials is expected to generate evolutionary change. Nevertheless, we explicitly test if selection gradients vary more among years and/or subpopulations than expected under drift alone following [14,75]. In short, we fitted a generalised linear mixed model with a Poisson error family and a log link function with annual genetic contribution (multiplied by two to ensure all values are integers) as the response variable. Predictors were clutch size (centred on the annual mean clutch size for each subpopulation) and subpopulation (i.e., breeding population; West or East). The interaction of year and subpopulation was fitted as a random effect, in addition to an observation-level random effect to model overdispersion [76]. To test for variation in the slope of the regression of annual genetic contribution on clutch size among years and/or subpopulations, we compared a random slopes model in which the slope was allowed to vary among subpopulations and years to a random intercept-only model using a likelihood ratio test in which twice the difference in log-likelihood was assumed to follow a mixture of $\chi_{\text{1}}^{\text{2}}$ and $\chi_{\text{2}}^{\text{2}}$ distributions. Models were fitted using the glmer() function from the lme4 package [v.2.0.6; 77].

The breeder’s equation predicts the *phenotypic* change from one *generation* to the next as the product of heritability and selection differential [5]. Here, however, we need to predict the *genetic* change from 1 *year* to the next, and the breeding population in the next year is partially made up of survivors from the previous year (mean inter-annual breeder survival for a nonexperimental year: West 0.28, East 0.44). We therefore instead use the Price equation [71,78] to derive the expected annual change in mean breeding value from year *t* to year *t* + 1 from the strength of selection in year *t*.

The change in mean breeding value across all female *and* male breeders from year *t* to year *t* + 1 is:


$$\begin{array}{c} {\Delta \mu_{a_{t}}=\mu }_{a_{t+\text{1}}}-\mu_{a_{t}} \end{array}$$

For brevity, we refer to this quantity as $\Delta \overset{―}{a}$ in the main text but since our population is defined by our sample, $\Delta \overset{―}{a}=\Delta {\mu_{a}}_{t}$. Following Price [71,78], $\Delta \mu_{a_{t}}=\sigma_{a_{t},w_{t}}$, where $a_{t}$ is the breeding value for clutch size of male and female breeders in year *t*, and $w_{t}$ their annual genetic success. Note that here we are assuming selective neutrality of variation in the breeding value for clutch size in males. Hence, we calculate the covariance between $a_{t}$ and $w_{t}$ for female breeders only, and then halve this to obtain $\Delta \mu_{a_{t}},$ which is the change in mean breeding value for males and females combined [46].

Selection on clutch size in year *t* is estimated as $\sigma_{z_{t},w_{t}}$, where $z_{t}$ is the clutch size of female breeders in year *t*. In the absence of a third variable mediating the covariance between clutch size and annual fitness (i.e., the relationship between clutch size and annual fitness is causal), the *expected* ratio of $\sigma_{a_{t},w_{t}}$ to $\sigma_{z_{t},w_{t}}$ (i.e., the conversion efficiency of phenotypic selection to genetic response) is equal to $\sigma_{a_{t}}^{\text{2}}/\sigma_{z_{t}}^{\text{2}}$ (the within-year heritability $h_{within-year}^{\text{2}}$) divided by two. This within-year heritability is influenced by among-year variation in $\sigma_{a_{t}}^{\text{2}}$ and $\sigma_{e_{t}}^{\text{2}}$ (where $e_{t}$ represents the environmental deviation for female clutch size in year *t*), and will thus differ from the heritability as estimated by the animal model, which applies to the base population. Since we assume


$$\begin{array}{c} \sigma_{a_{t},e_{t}}=\text{0}, \end{array}$$

it follows that


$$\begin{array}{c} \sigma_{a_{t}}^{\text{2}}=\sigma_{a_{t},z_{t}} \end{array}$$

and hence,


$$\begin{array}{c} h_{within-year}^{\text{2}}=\frac{\sigma_{a_{t}}^{\text{2}}}{\sigma_{z_{t}}^{\text{2}}}=\frac{\sigma_{a_{t},z_{t}}}{\sigma_{z_{t}}^{\text{2}}} \end{array}$$

From this it follows that the expected proportion of the selection differential that is transmitted to the next year via additive genetic effects is estimated as the slope of the regression of $a_{t}$ on $z_{t}$ ($b_{z_{t},a_{t}}$) divided by two.

In addition to quantifying the interannual change in the mean breeding value for clutch size, we also quantified its *in situ* component, referred to as $\Delta {\overset{―}{a}}_{in\;situ}$ in the main text, as the interannual change in the mean breeding value when excluding the influence of post-selection immigration into the breeding population,


$$\begin{array}{c} \Delta {\overset{―}{a}}_{{in\;situ}_{t}}=\mu_{a_{{non-immigrant\;breeder}_{t+\text{1}}}}-\mu_{a_{b{reeder}_{t}}} \end{array}$$

where $\mu_{a_{{non-immigrant\;breeder}_{t+\text{1}}}}$ represents the mean breeding value of breeders in year *t* + 1 that are *not* newly recruited immigrants (i.e., the overall mean breeding value of surviving breeders from year *t* and their recruited offspring [39,79]).

### Decomposing evolutionary change

Since the animal model quantifies individual breeding values, it is possible to attribute temporal change in the mean breeding value of the breeding population to contributing demographic processes according to a mass balance equation, as algebraically formalised by Kerr and Godfrey-Smith [80] and empirically applied to demographic decomposition of phenotypic ageing [81–83], plant community change [80,84] and uptake of accounting innovations [85]. In our animal model, clutch size breeding values were fixed with respect to age, so inferred change in the mean breeding value of the breeding population between consecutive years (*G*) is attributable to the appearance and disappearance of individuals from that population. If we ignore the possibility of reproductive skipping and post-recruitment dispersal, then


$$\begin{array}{c} G\;=\;m+r \end{array}$$

where *m* represents the contribution of mortality and *r* represents that of recruitment to the breeding population. We are now considering the genetic change *before* a focal year *t* (i.e., the change in the mean breeding value of the breeding population from year *t*-1 to year *t*):


$$\begin{array}{c} G_{t}=\nabla_{a_{t}}=\mu_{a_{b{reeder}_{t}}}-\mu_{a_{b{reeder}_{t-\text{1}}}} \end{array}$$

For the purposes of decomposing the interannual genetic change into the contributing effects of mortality and recruitment, we recognise two demographic classes: recruits and survivors. If $\mu_{a_{survivor_{t}}}$ is the mean breeding value of established breeders in year *t* (i.e., those also observed breeding in year *t*-1), we can calculate the interannual genetic change in the breeding population attributable to mortality as


$$\begin{array}{c} m_{x}=\mu_{a_{survivor_{t}}}-\mu_{a_{b{reeder}_{t-\text{1}}}\;} \end{array}$$

Similarly, the impact of new recruits to the breeding population can be quantified as


$$\begin{array}{c} r_{t}=\;\mu_{a_{b{reeder}_{t}}}-\mu_{a_{survivor_{t}}} \end{array}$$

Since we are interested in contrasting immigration with the influence of in situ demographic change, we subdivided recruits according to their natal origin: (*i*) native recruits originating within the focal area, and (*ii*) immigrants that originate elsewhere, such that:


$$\begin{array}{c} =r_{native}+r_{immigrant} \end{array}$$

However, calculating the contributions of native versus immigrant recruits requires that the order of recruitment be known (i.e., whether or not, e.g., native recruits are contributing to interannual genetic change of the breeding population before or after immigrants have themselves been recruited), since this influences both the size and mean breeding value of the host breeding population they are joining at the point of recruitment. While some have speculated that earlier recruitment of native fledgling great tits explains their superior breeding performance over immigrants [45], conspicuously little is known about the life-history period between fledging and the initial breeding attempt that confirms an individual’s successful recruitment [86]. In the absence of clear evidence indicating otherwise, we therefore adopted a compromise model that assumes equal recruitment phenology. For this, the genetic impact of each class of recruits is calculated as the mean of the two extreme scenarios with respect to the temporal sequence of recruitment events, i.e., first that recruitment of local-fledged individuals occurs before that of immigrant individuals, and secondly the converse arrangement:


$$\begin{array}{c} {𝑟}_{{native}_{t}}=\frac{\mu_{{𝑎}_{{breeder}_{t}}}-\mu_{{𝑎}_{{immigrant}_{t}}}}{2}+\frac{\mu_{{𝑎}_{\neg {native}_{t}}}-\mu_{{𝑎}_{{survivor}_{t}}}}{2} \end{array}$$

$$\begin{array}{c} {𝑟}_{{immigrant}_{t}}=\frac{\mu_{{𝑎}_{{breeder}_{t}}}-\mu_{{𝑎}_{{native}_{t}}}}{2}+\frac{\mu_{{𝑎}_{\neg {immigrant}_{t}}}-\mu_{{𝑎}_{{survivor}_{t}}}}{2} \end{array}$$

for which, e.g., $\mu_{{𝑎}_{\neg {native}_{t}}}$ represents the mean breeding value of all breeders except native recruits. Uncertainty in individual breeding values is propagated throughout the analysis by conducting these demographic decomposition calculations on each sampled iteration, taking the resulting distribution for each statistic to represent its posterior.

As for many study populations of wild animals that follow an annual breeding cycle, reproductive phenotypes are missing for some survivors that were observed breeding again in the same patch in future years (observation probabilities of 87.6% and 87.7% for female breeders in the West and East, respectively). This could result from various processes, including early breeding failure [87], nesting in a natural cavity [88], or reproductive skipping [89]. While we cannot be sure of the relative importance of these possible explanations, 5.0% of all known breeders in our focal patches appeared to ‘skip’ reproduction in at least 1 year in this way (38 of 2,167 identified breeders in the West [1.8%]; 312 of 4,850 [6.4%] identified breeders in the East). To simplify the decomposition of genetic change in the breeding populations, we exclude individuals with ‘missing’ breeding-years, thus assuming that they are a random subset of the breeding population with respect to their breeding values for clutch size. In total, 0.1% (10 of 7,007) of observed breeders bred in both West and East (i.e., dispersed post-reproductively), but because of its rarity we ignore this process as a driver of within-patch genetic change and pool the movement of these individuals with mortality and recruitment from the perspective of the patches they moved from and to, respectively (as would be our assumption if we had breeding observations for only one breeding population). Note, also, that since we are concerned with changes in the mean breeding value of clutch size, we do not restrict consideration to females: males have an estimable breeding value, informed by the observed clutch size of their female relatives.

### Ancestry of local-origin breeders

Following earlier work [43], we used data on grandparental origins as a proxy for quantifying selection on mainland ancestry among the two breeding populations. For each breeder of *local* origin (i.e., excluding all immigrant recruits), we calculated how many of their grandparents were of *known* origin (i.e., fledged from a nestbox on the island). We use this genealogy-based count as an index of Vlielandic ancestry, thus assuming unknown-origin birds (and their parents) are derived from a mainland population.

### Modelling multiple sources of founders

Our animal model assumes that all founders are derived from a single source population. Given our focus on the genetic impact of immigration, we explored the model-dependency of our results using alternative models that explicitly recognise the potential for unknown-origin breeders (S6 Fig) to have genetically distinct ancestors from island-origin founders [90,91].

We defined island-level founders as those individuals of unknown origin that were known or estimated (on the basis of minimum possible age, if exact age was unknown) to have fledged before 1963, which is the first year that the proportion of unknown-origin breeders drops below the long-term mean (1955–2023; S6 Fig). All other unknown parents were initially assigned to one of four immigrant group identities on the basis of their offspring’s year-of-origin, allowing this genetic distinction between island-origin and immigrant individuals to be temporally dynamic. Temporal divisions corresponded with the periods defined above: pre-experimental immigrants (offspring cohorts: 1963–1994); experiment-era immigrants (1995–2002); post-experimental immigrants (2003–2010); and legacy-era immigrants (2010–2022). This modified pedigree was the basis of an augmented inverse relatedness matrix (A* [90]), constructed using the makeAinv() function of the nadiv package (v.2.18.0 [92]), with the five explicit genetic groups listed first. A* recognises individuals’ immigrant ancestry by, in effect, treating these genetic groups as (hermaphroditic and selfing) parents. Following Wolak and Reid [90], we added a constant of 0.1 to the diagonal elements of the first five rows of A* (corresponding to the genetic group identities; we also ran models with alternative values of 1 and 10 added to these diagonal elements of A* to confirm that our choice of constant was not having a strong influence on results: S6 Table).

This five-group animal model gave quantitatively consistent effect estimates (S6 Table), and yielded broadly overlapping breeding value posteriors for the four immigrant groups (S8 Fig). Accordingly, we pooled our recognised groups into a single population from which all individuals of unknown origin that fledged more recently than 1962 were assumed to be sourced. This revised genetic groups animal model (S7 Table) indicated that this immigrant source population was genetically distinctive to the ‘Vlieland’ lineage, having a larger mean breeding value for clutch size (Δ=0.39 [0.01,0.78] eggs; S9*b* Fig). However, because of the limited informativeness of this ancestry division (S10 Fig), the mean breeding value for both genetic groups was estimated with large uncertainty (S9 Fig). Consequently, the absolute values of individual breeding values were estimated with very low precision by this genetic groups animal model, and this uncertainty propagated to population-level statistics (S11*a* Fig). Importantly, however, analyses based on genetic differences are near-identical to those from a group-naïve animal model (S11*b* Fig). As such, we rely on the group-naïve animal model for our main results.

## Supporting information

S1 Fig Mean clutch size of newly recruited female members of the breeding populations of West and East matched by their site of natal origin, for each of the four periods within the focal timespan. Period boundaries reflect the delay between fledging and breeding in the first year of age. The data and code needed to generate this Figure are archived in the Zenodo online repository [93].
(PDF)

S2 Fig Posterior distributions of the regression of female breeders’ breeding value on their observed clutch sizes, as an alternative to reliance on the trait heritability. These posterior distributions were multiplied by the year- and subpopulation-specific selection differentials (and the resulting product halved, to account for sex-limited trait expression) to generate posterior distributions for the null expectation for the evolutionary response to selection. The data and code needed to generate this Figure are archived in the Zenodo online repository [93].
(PDF)

S3 Fig Mean interannual changes in the estimated mean breeding value of breeders in (a) the West and (b) the East for each of four time periods, and their decomposition into contributions via mortality, local recruitment and recruitment of immigrants. Bar heights represent the posterior mean from our animal model of clutch size, error bars span the 95% highest probability density interval. The data and code needed to generate this Figure are archived in the Zenodo online repository [93].
(PDF)

S4 Fig Annual proportions of newly recruited breeders that are of unknown origin (i.e., were first caught post-fledging). Best-fit lines from a linear regression are plotted (slope estimates: West: −0.30 ± 0.17 percent/yr, *t*_42_ = −1.77, *P* = 0.083; East: 0.32 ± 0.08 percent/yr, *t*_42_ = 3.92, *P* < 0.001). The data and code needed to generate this Figure are archived in the Zenodo online repository [93].
(PDF)

S5 Fig Annual posterior mean breeding values for clutch size of unknown-origin recruits to the breeding populations between 1980 and 2023. Error bars depict the 95% highest probability density interval. The data and code needed to generate this Figure are archived in the Zenodo online repository [93].
(PDF)

S6 Fig Composition of the annual breeding populations with respect to the origins of newly recruited breeders. Those individuals who were recorded breeding in previous years are classed as *established breeders. Unknown-origin recruits* are individuals in their first observed year of breeding that hatched outside the nestbox study. Scaling is identical across the two plots. The data and code needed to generate this Figure are archived in the Zenodo online repository [93].
(PDF)

S7 Fig Proportion of the combined breeding populations of West and East that is of unknown natal origin, with the mean (1955–2023) plotted as a dashed line. The data and code needed to generate this Figure are archived in the Zenodo online repository [93].
(PDF)

S8 Fig Posterior probability distributions of the mean breeding values for each of five groups to which unknown-origin breeders were assigned based on their known (or estimated) hatching year. The data and code needed to generate this Figure are archived in the Zenodo online repository [93].
(PDF)

S9 Fig Posterior probability distributions of (*a*) the mean breeding value for the two genetic groups to which unknown-origin breeders were assigned on the basis of their known or estimated hatching year, and (*b*) the difference in the breeding value for clutch size between island-level founders and immigrant founders. The data and code needed to generate this Figure are archived in the Zenodo online repository [93].
(PDF)

S10 Fig The immigrant ancestry status of observed breeders for each year of the population study from 1955 to 2023 in (*a*) the West and (*b*) the East. Lines link the annual means. The data and code needed to generate this Figure are archived in the Zenodo online repository [93].
(PDF)

S11 Fig Annual mean breeding value for clutch size (*top*) and interannual genetic change in clutch size attributable to immigrant recruitment (*bottom*) for the West (*left*) and East (*right*) breeding populations, as estimated by alternative animal models making different assumptions about the number of source groups contributing to the ancestral ‘base population’. The data and code needed to generate this Figure are archived in the Zenodo online repository [93].
(PDF)

S1 TableVariance decomposition of clutch size in Vlieland’s nestbox-breeding great tits obtained from the animal model used to generate the predicted breeding for all subsequent analyses.We provide effect estimates as proportions of both (*a*) observed phenotypic variance across the study period, and (*b*) within-year phenotypic variance: since we consider the evolutionary dynamics of these populations from an annual perspective, we apply within-year heritability to our predictions of annual adaptive change in clutch size. Female age (1 year-old versus older) and nestbox area (see Fig 1, but note that two such areas are recognised within the largest [Easternmost] wood) were fitted as fixed effects. $n_{observations}=\text{7}\text{1}\text{9}\text{8}$ ($n_{{observations}_{West}}=\text{1}\text{5}\text{9}\text{7};$ $n_{{observations}_{East}}=\text{4}\text{7}\text{1}\text{1}$, $n_{{observations}_{Village}}=\text{8}\text{9}\text{0}$), $n_{females}=\text{4}\text{2}\text{5}\text{7}$ ($n_{{females}_{West}}=\text{1}\text{1}\text{8}\text{4};$ $n_{{females}_{East}}=\text{2}\text{5}\text{9}\text{2}$; $n_{{females}_{Village}}=\text{5}\text{7}\text{0}$), range of years: 1955–2023. The data and code needed to generate this Table are archived in the Zenodo online repository [93].(DOCX)

S2 TableMean annual selection for clutch size during each of four periods within the study.Linear selection differentials, S, are the mean annual covariance of clutch size and annual fitness via annual genetic success (combining own survival and the recruitment of offspring from all clutches laid that year). To aid comparisons with selection on other traits and populations, we also provide mean-standardised linear selection gradients, $\beta_{\mu }$ [94,95]. The data and code needed to generate this Table are archived in the Zenodo online repository [93].(DOCX)

S3 Table Mean annual genetic change in clutch size within each of the four periods, and the posterior probabilities that changes of such magnitudes occurred in the absence of selection. The data and code needed to generate this Table are archived in the Zenodo online repository [93].
(DOCX)

S4 Table Decomposition of evolutionary changes in clutch size of the breeding populations of West and East before, during and after an artificial selection experiment, as inferred by an animal model. Values are the posterior means and 95% highest probability density interval for the slope of a linear regression of mean breeding value for clutch size on time. Overall genetic change is decomposed into change attributable to mortality, native (i.e., local origin) recruitment and immigrant recruitment. Note that there is a one-year lag between the clutch removal of the selection experiment and the observable response on the genetic composition of the breeding population, which is reflected in the stated boundaries of the four periods. Note also that individuals unobserved as breeders for at least 1 year in which they are confirmed as having been alive (by dint of being observed breeding in a later year) were excluded (see Materials and methods), which accounts for the minor quantitative discrepancies in the overall rates of genetic change in S3 Table. The data and code needed to generate this Table are archived in the Zenodo online repository [93].
(DOCX)

S5 Table Variance decomposition of clutch size in Vlieland’s nestbox-breeding great tits obtained from an animal model with an alternative prior structure. As for the animal model used as the basis for the main results, female age (1 year-old versus older), nestbox area and period-specific temporal trends were fitted as fixed effects. The data and code needed to generate this Table are archived in the Zenodo online repository [93].
(DOCX)

S6 Table Variance decomposition of clutch size in Vlieland’s nestbox-breeding great tits obtained from an animal model that explicitly recognised five genetically distinct source populations. As for the animal model used as the basis for the main results, female age (1 year-old versus older), nestbox area, and period-specific temporal trends were fitted as fixed effects. The data and code needed to generate this Table are archived in the Zenodo online repository [93].
(DOCX)

S7 Table Variance decomposition of clutch size in Vlieland’s nestbox-breeding great tits obtained from an animal model that explicitly recognised two genetically distinct source populations. As for the animal model used as the basis for the main results, female age (1 year-old versus older), nestbox area, and period-specific temporal trends were fitted as fixed effects. The data and code needed to generate this Table are archived in the Zenodo online repository [93].
(DOCX)
