# Supplementary material for: Gene flow drives periods of both evolutionary stasis and change in a wild bird population
Source: PLoS Biol. 2026 Sep 21;24(9):e3004004. doi: 10.1371/journal.pbio.3004004 (PMC13630318; doi:10.1371/journal.pbio.3004004)
Supplement: Supplementary file 1 [file pbio.3004004.s001.pdf]

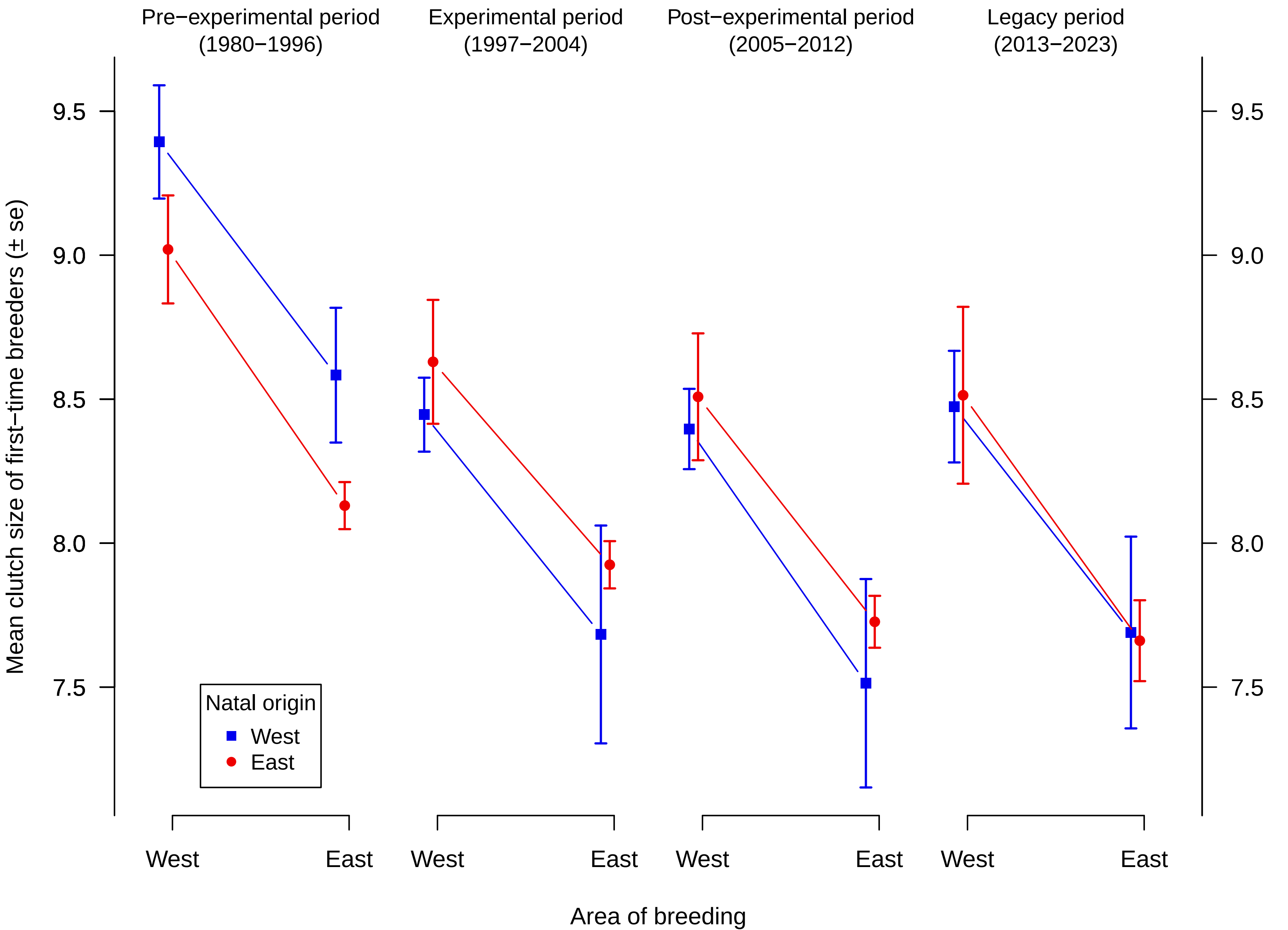
Pre−experimental period
(1980−1996)
Experimental period
(1997−2004)
Post−experimental period
(2005−2012)
Legacy period
(2013−2023)
Mean clutch size of first−time breeders (± se)
9.5
9.0
8.5
8.0
7.5
Natal origin
West
East
West
East
West
East
West
East
West
East
Area of breeding
