# Supplementary material for: Gene flow drives periods of both evolutionary stasis and change in a wild bird population
Source: PLoS Biol. 2026 Sep 21;24(9):e3004004. doi: 10.1371/journal.pbio.3004004 (PMC13630318; doi:10.1371/journal.pbio.3004004)
Supplement: Supplementary file 3 [file pbio.3004004.s003.pdf]

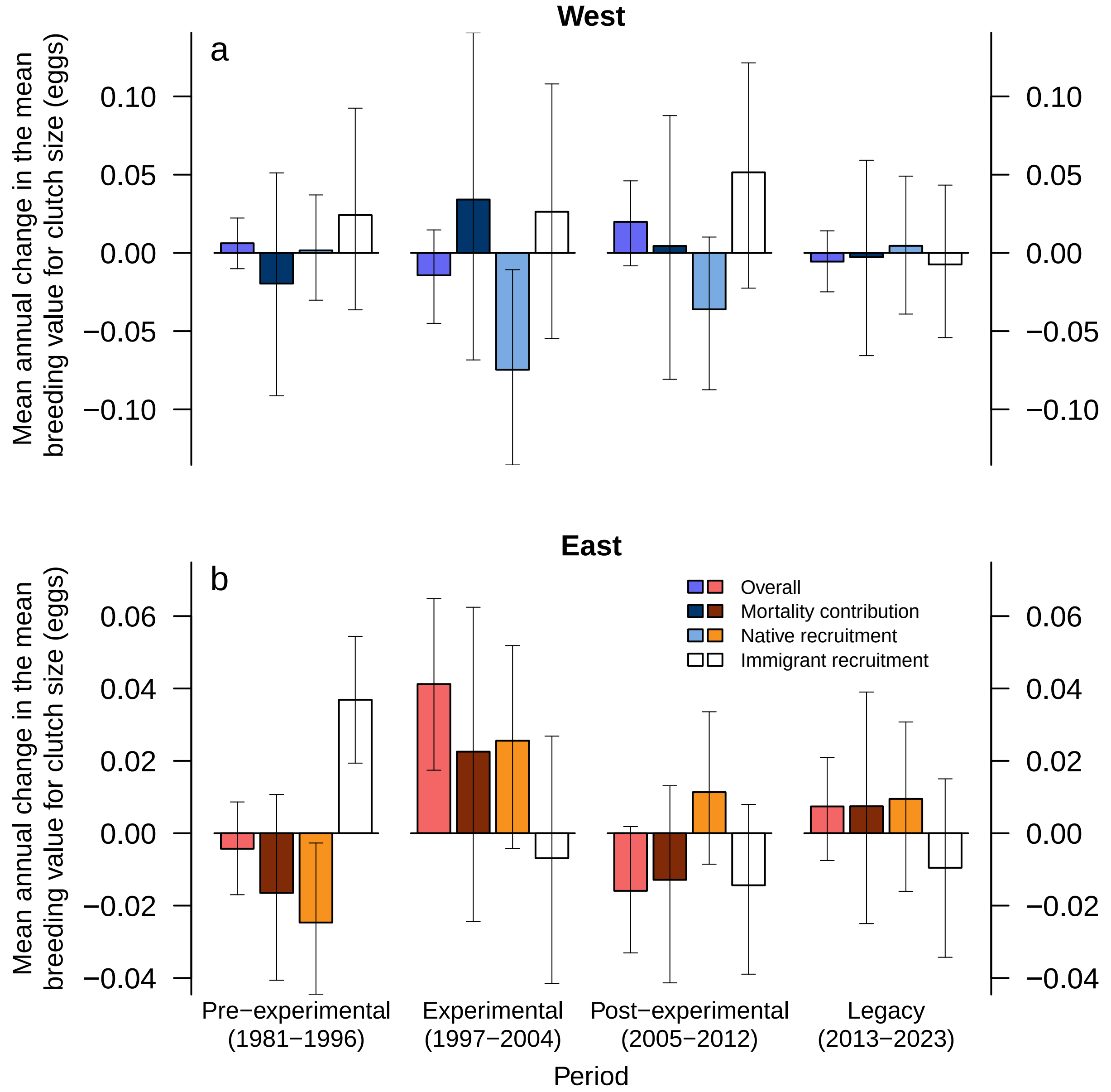
West
a
0.10
0.05
0.00
−0.05
−0.10
Mean annual change in the mean breeding value for clutch size (eggs)
East
b
Overall
Mortality contribution
Native recruitment
Immigrant recruitment
0.06
0.04
0.02
0.00
−0.02
−0.04
Pre−experimental (1981−1996)
Experimental (1997−2004)
Post−experimental (2005−2012)
Legacy (2013−2023)
Period
