# Supplementary material for: Gene flow drives periods of both evolutionary stasis and change in a wild bird population
Source: PLoS Biol. 2026 Sep 21;24(9):e3004004. doi: 10.1371/journal.pbio.3004004 (PMC13630318; doi:10.1371/journal.pbio.3004004)
Supplement: Supplementary file 4 [file pbio.3004004.s004.pdf]

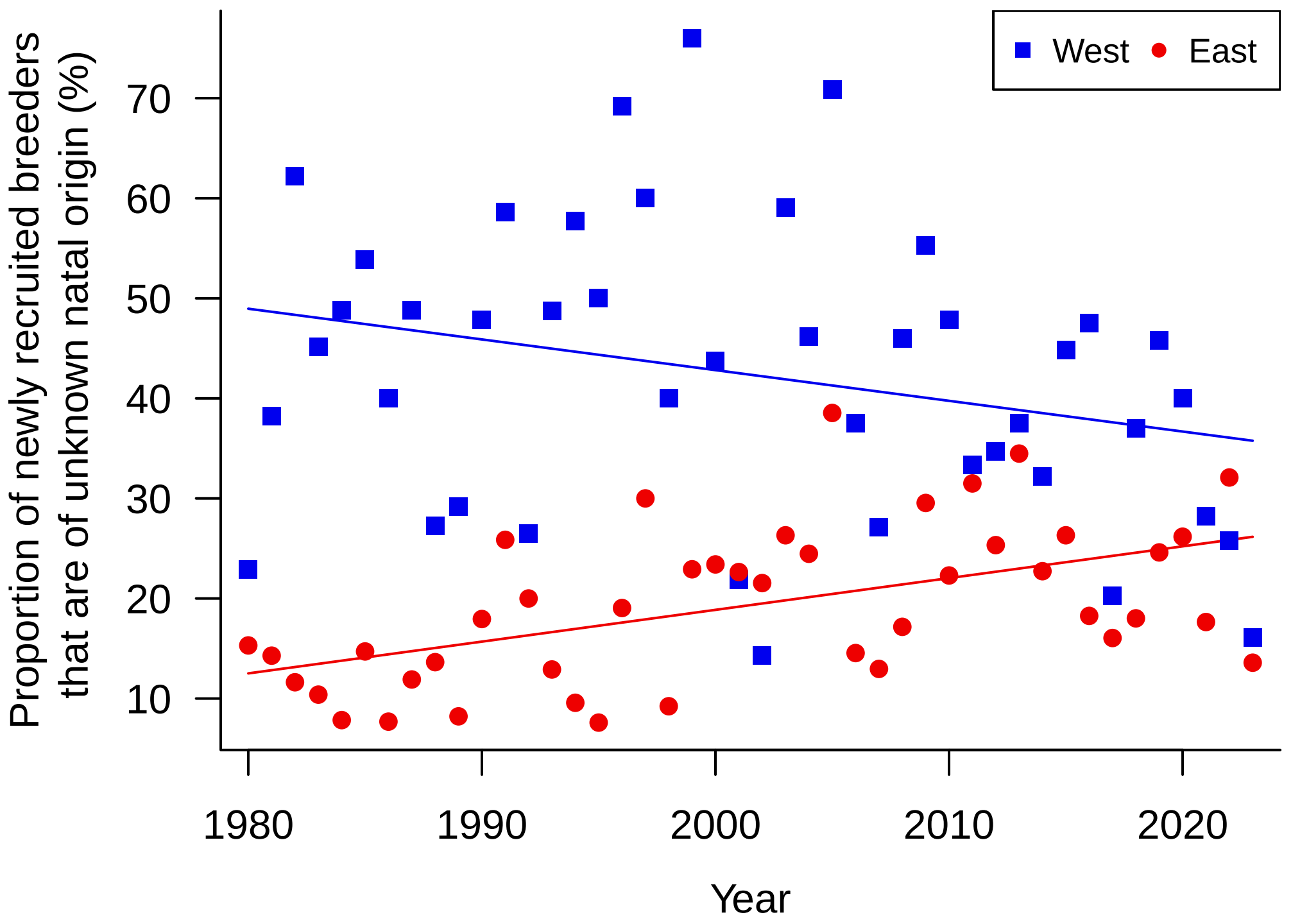
West
East
Proportion of newly recruited breeders
that are of unknown natal origin (%)
10
20
30
40
50
60
70
1980
1990
2000
2010
2020
Year
