# Supplementary material for: Gene flow drives periods of both evolutionary stasis and change in a wild bird population
Source: PLoS Biol. 2026 Sep 21;24(9):e3004004. doi: 10.1371/journal.pbio.3004004 (PMC13630318; doi:10.1371/journal.pbio.3004004)
Supplement: Supplementary file 5 [file pbio.3004004.s005.pdf]

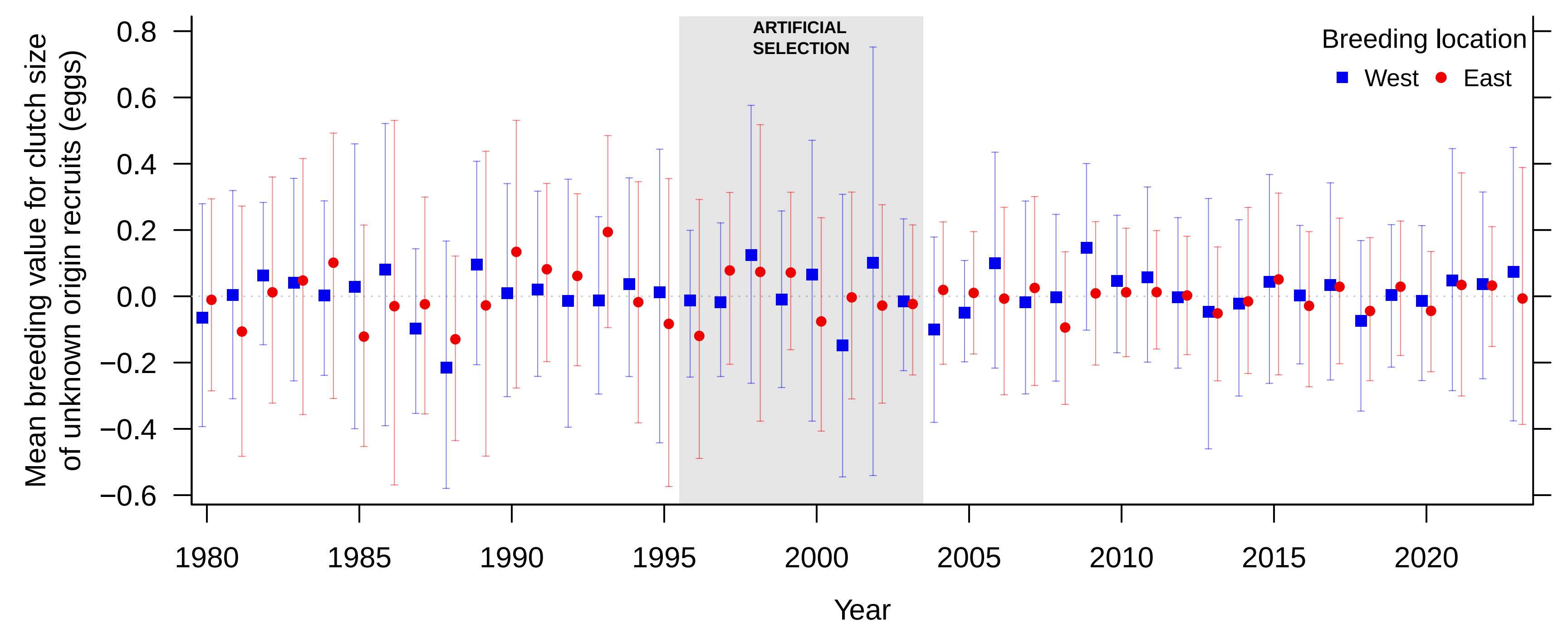

ARTIFICIAL SELECTION
Breeding location
West
East
Mean breeding value for clutch size of unknown origin recruits (eggs)
0.8
0.6
0.4
0.2
0.0
−0.2
−0.4
−0.6
1980
1985
1990
1995
2000
2005
2010
2015
2020
Year
