# Supplementary material for: Gene flow drives periods of both evolutionary stasis and change in a wild bird population
Source: PLoS Biol. 2026 Sep 21;24(9):e3004004. doi: 10.1371/journal.pbio.3004004 (PMC13630318; doi:10.1371/journal.pbio.3004004)
Supplement: Supplementary file 6 [file pbio.3004004.s006.pdf]

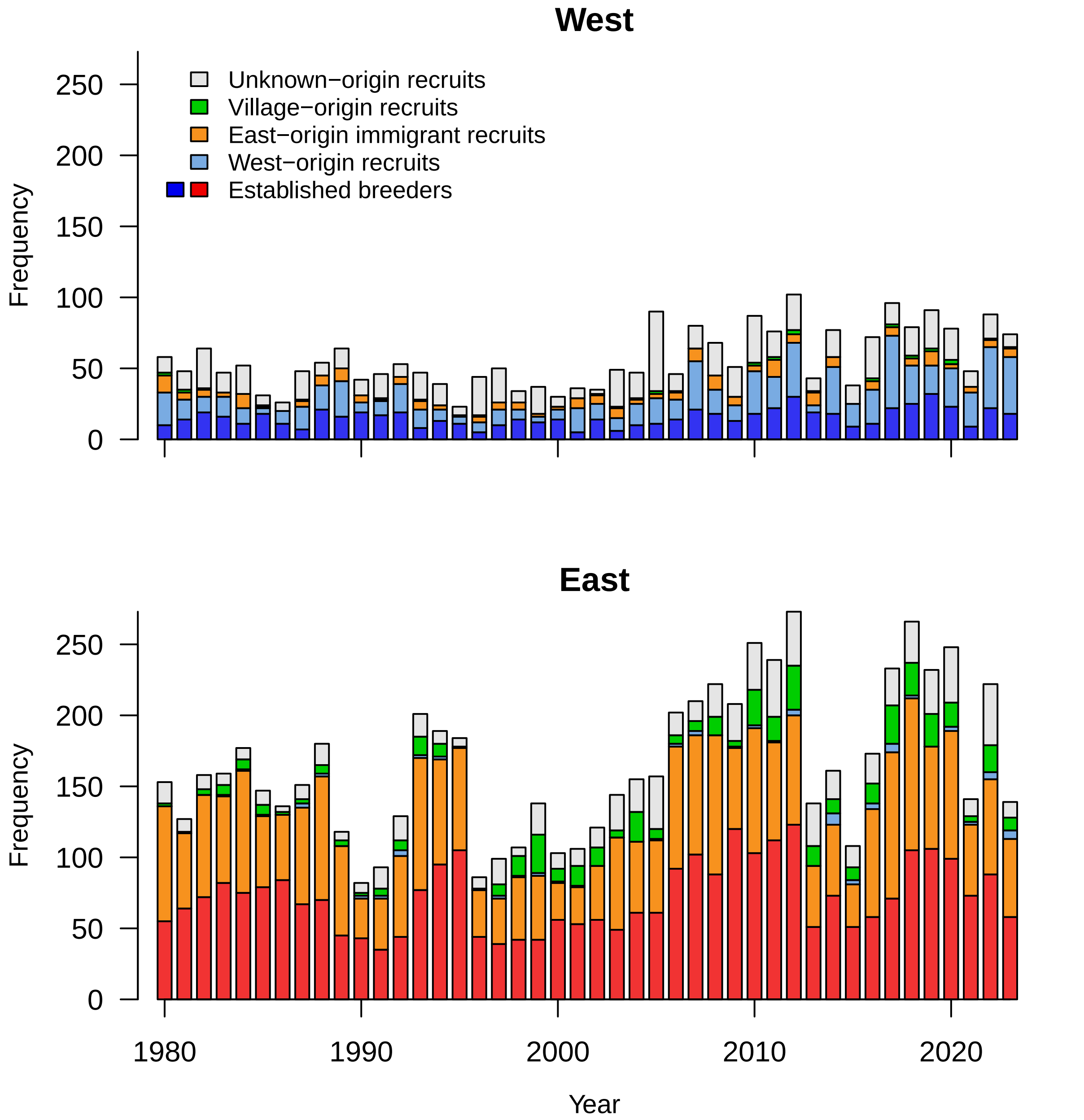
West
Unknown−origin recruits
Village−origin recruits
East−origin immigrant recruits
West−origin recruits
Established breeders
Frequency
0
50
100
150
200
250
East
Frequency
0
50
100
150
200
250
1980
1990
2000
2010
2020
Year
