# Supplementary material for: Gene flow drives periods of both evolutionary stasis and change in a wild bird population
Source: PLoS Biol. 2026 Sep 21;24(9):e3004004. doi: 10.1371/journal.pbio.3004004 (PMC13630318; doi:10.1371/journal.pbio.3004004)
Supplement: Supplementary file 7 [file pbio.3004004.s007.pdf]

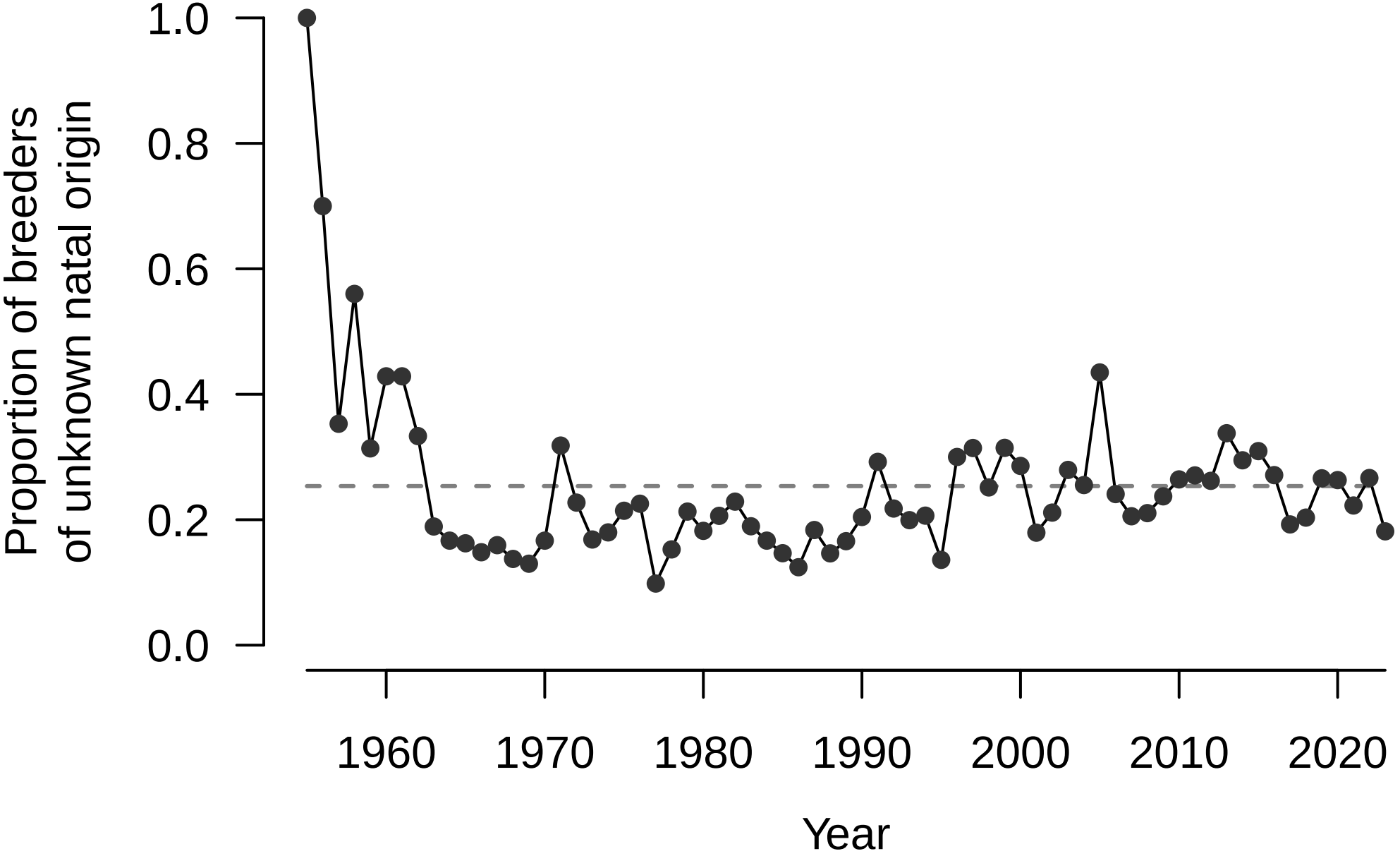

Proportion of breeders
of unknown natal origin
1.0
0.8
0.6
0.4
0.2
0.0
1960
1970
1980
1990
2000
2010
2020
Year
