# Supplementary material for: Gene flow drives periods of both evolutionary stasis and change in a wild bird population
Source: PLoS Biol. 2026 Sep 21;24(9):e3004004. doi: 10.1371/journal.pbio.3004004 (PMC13630318; doi:10.1371/journal.pbio.3004004)
Supplement: Supplementary file 8 [file pbio.3004004.s008.pdf]

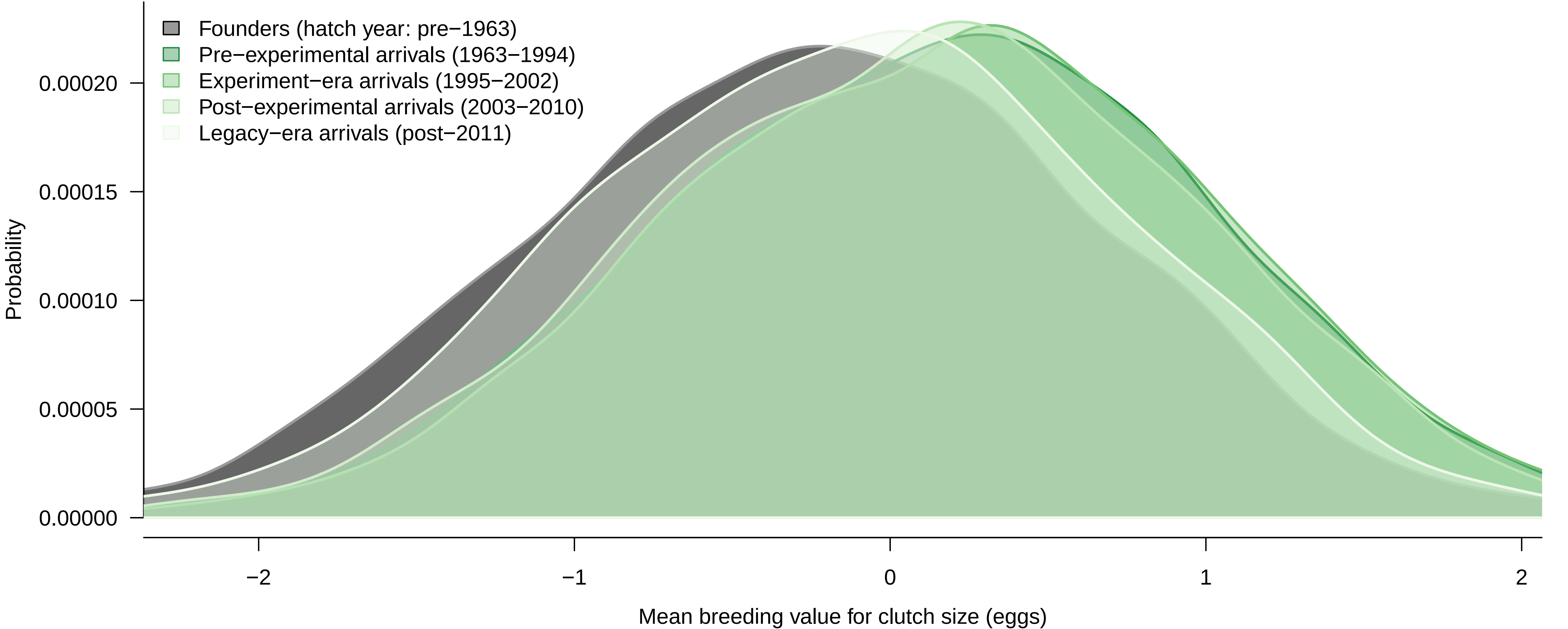
Founders (hatch year: pre−1963)
Pre−experimental arrivals (1963−1994)
Experiment−era arrivals (1995−2002)
Post−experimental arrivals (2003−2010)
Legacy−era arrivals (post−2011)
Probability
0.00000
0.00005
0.00010
0.00015
0.00020
−2
−1
0
1
2
Mean breeding value for clutch size (eggs)
