# Supplementary material for: Gene flow drives periods of both evolutionary stasis and change in a wild bird population
Source: PLoS Biol. 2026 Sep 21;24(9):e3004004. doi: 10.1371/journal.pbio.3004004 (PMC13630318; doi:10.1371/journal.pbio.3004004)
Supplement: Supplementary file 9 [file pbio.3004004.s009.pdf]

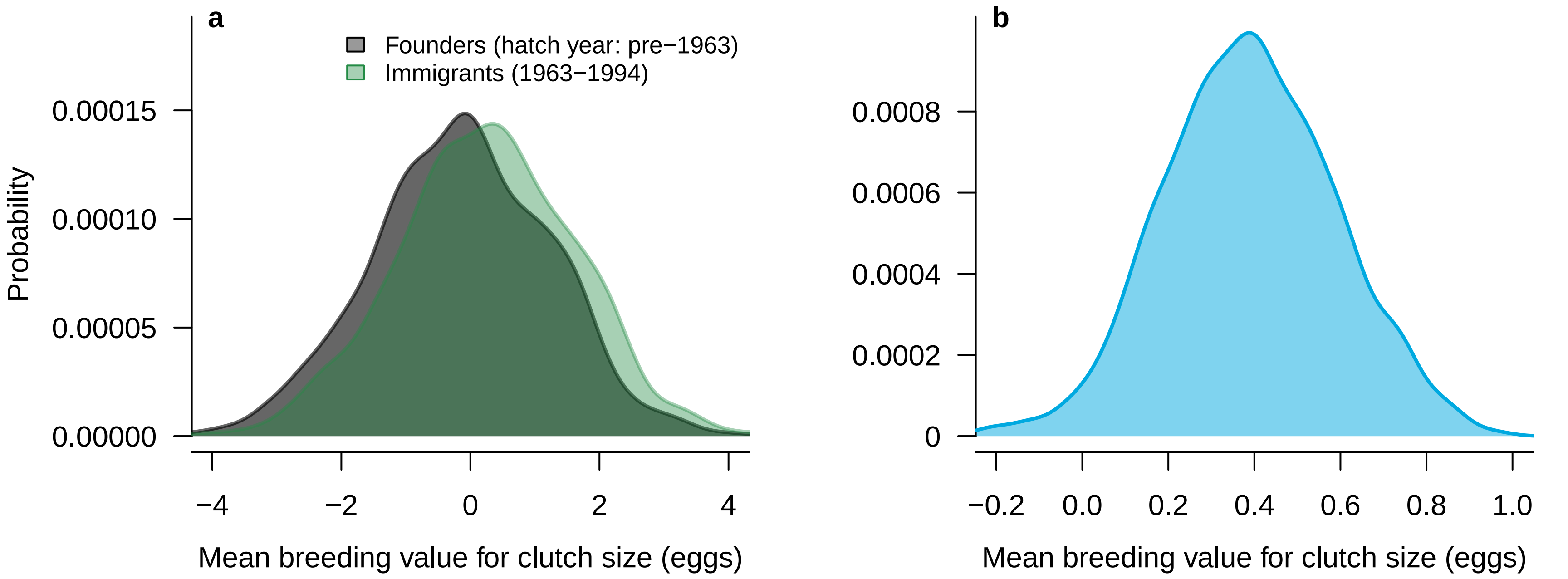
a
Founders (hatch year: pre−1963)
Immigrants (1963−1994)
Probability
0.00015
0.00010
0.00005
0.00000
−4
−2
0
2
4
Mean breeding value for clutch size (eggs)
b
0.0008
0.0006
0.0004
0.0002
0
−0.2
0.0
0.2
0.4
0.6
0.8
1.0
Mean breeding value for clutch size (eggs)
