# Supplementary material for: Gene flow drives periods of both evolutionary stasis and change in a wild bird population
Source: PLoS Biol. 2026 Sep 21;24(9):e3004004. doi: 10.1371/journal.pbio.3004004 (PMC13630318; doi:10.1371/journal.pbio.3004004)
Supplement: Supplementary file 11 [file pbio.3004004.s011.pdf]

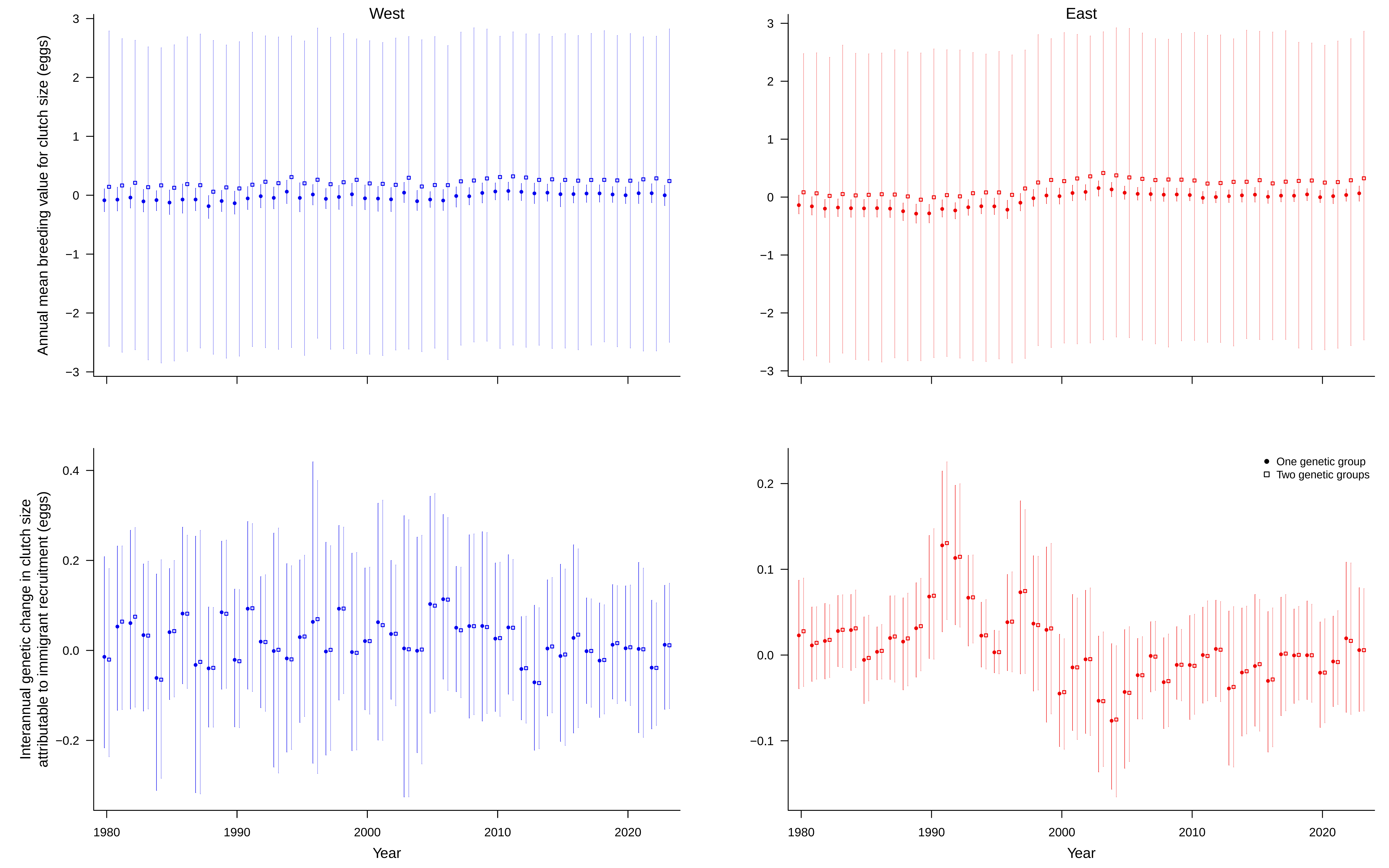

West
East
Annual mean breeding value for clutch size (eggs)
Interannual genetic change in clutch size attributable to immigrant recruitment (eggs)
One genetic group
Two genetic groups
Year
Year
