# Supplementary material for: Gene flow drives periods of both evolutionary stasis and change in a wild bird population
Source: PLoS Biol. 2026 Sep 21;24(9):e3004004. doi: 10.1371/journal.pbio.3004004 (PMC13630318; doi:10.1371/journal.pbio.3004004)
Supplement: Supplementary file 12 [file pbio.3004004.s020.pdf]

Review of ‘Paradox lost? Gene flow determines trait evolution in a wild bird population’

Joel Pick

2025-08-09

This study looks at a 44 year dataset of two island populations of great tits, that initially differed in clutch size, a key life history trait. The study charts the changes in breeding values for clutch size through 15 years pre-experiment, then an 8 year artificial selection experiment to reverse this difference, and then for 20 years post artificial selection. This study is done in the context of earlier work on the initial differences between the populations, including the roles of local adaptation and immigration (Postma and Van Noordwijk 2005, Postma et al 2009), and the selection experiment itself (Postma et al 2007). In this study, the authors seek to attribute the differences and changes in breeding values between populations across these time periods to the relative influences of selection and immigration. This study uses a remarkable dataset, to address an interesting, timely, and largely ignored question in wild quantitative genetics.

Generally the writing style is very clear and easy to follow, although in several places I felt I needed more information, and particular parts of the method need clarification. I also felt it could have been put more firmly in the context of the previous work, which already established the important role of immigration in this population. I don’t think that this previous work diminishes the current contribution, but not knowing the previous work well meant that part of the results were hard to contextualise.

I have some major comments that are maybe due to my own ignorance or misunderstanding, but could at least do with some additional discussion in the manuscript. In particular, as there is a lot of complex analysis, which often using estimates from several different models, there is opportunity for patterns to arise due to statistical artefacts - it would be nice to be reassured that this wasn’t the case, or what the potential for these artefacts to arise was. I apologise for the length of the review, but this is the most interesting manuscript I have

reviewed in a long while.

Major Comments

1. Overall framing

   This is clearly up to the authors, but I find the overall framing a little confusing. I have a bit of a stumbling block over how the paradox of stasis fits in here. It seems like the starting point/framing of the manuscript is selection/local adaptation, and why we don't find change in the presence of strong, persistent directional selection (paradox of stasis). Whilst I would a priori think that clutch size was under strong, persistent directional selection as it is a component of fitness (although various previous studies have suggested that it is under stabilising selection in tit populations), it doesn't appear to be under clear selection in these populations at all (see also Postma and Van Noordwijk 2005). To my mind, for there to be local adaptation in two close populations, there needs to be evidence of differing optima, or consistently different directional selection regimes in the two populations. Based on figure two and table S2 (and Postma and Van Noordwijk 2005), there doesn't seem to be clear evidence of directional selection on clutch size in either the same or opposite directions in the pre-experimental period. From the limited description on l318-328, it also seems like there is no clear stabilising selection, and no mention of differing optima between populations.

   The previous work on this population seems to show that local adaptation isn't the reason for the pre-experimental difference between populations, rather it is due to differential immigration from the mainland into the two island populations, with the mainland immigrants having larger eggs than the island birds, resulting in a larger egg size in the West population with greater immigration (Postma and Van Noordwijk 2005). This information seems lacking from the introduction, results and discussion, although a key bit of context for the framing of the study.

   If we are then not looking at a trait that is under strong, persistent directional selection, or with clear evidence of divergent optima between populations, it seems strange to phrase this so heavily in the context of the paradox of stasis. I'm not sure that this study, although a valuable demonstration of the role of migration and gene flow in microevolution on small spatial scales, tells us anything about resolving the paradox of stasis, as it seems in this case we would predict stasis through the lack of selection. Or at least, if it does, I think it needs far better signposting to these results.

The question may then be what happens when we place a trait under strong selection in the wild, and how do different processes (such as immigration) affect selection response. During the period of artificial selection, there was a response in the breeding values (Postma et al 2007, and this study), which shows that strong directional selection in these populations would have a response. When the selection stops the response is then swamped by immigration. This is phrased as a 'paradox of flux' (l 98), but I am not clear as to why there is a paradox. Given the generally high immigration rates in tit populations (and in this population, shown Figure S3), is it surprising that immigration is a major driver of return to a previous state after selection ends? I'm just thinking here that, theoretically, if I took two open subpopulations, and performed an artificial selection experiment on a selectively neutral trait in opposing directions, then given immigration rates of 20-50% (figure S3), I would expect a rapid return to mean trait value when selection stopped. If the point is that this is what we would expect, but this is not typically considered by researchers (with which I totally agree), then I think that the introduction should lean on this much more and articulate this prediction, rather than this result being surprising ('paradox of flux').

I am not saying that an experimental demonstration of this effect is not very important, interesting and useful, I'm just wondering whether, giving the existing knowledge of the system, would anything else have been expected. The impression I get from the manuscript is that this is somehow unexpected. The paradox of stasis, for my perspective, relates to traits under strong, persistent directional selection, for example juvenile body size.

For me then, it is not super clear how this study shows that immigration is working against selection in this population. Upon reading this several times, I think that a key paragraph is l145-157, but I don't think that the importance of this results is clearly signposted, or put well into the context of the previous work on the system. Here the demographic decomposition shows a reasonable impact of immigration in the western population, but not the eastern, during the experimental period. I think this would be expected, given the previous work on immigrations into the west and not the east, but this is not clearly articulated. There is then the effect of immigration in the legacy period in the east population, but these effects seem relatively small and uncertain, and it is difficult to separate the effects of drift (although there does seem to be phenotypic selection for larger clutches here, but I don't think that this is statistically demonstrated anywhere).

Again the framing is up to the authors (and I am happy to be shown wrong), but my

suggestion would be to put this much more firmly into the context of the previous work, and make predictions on how you would expect immigration and selection to balance each other in the two populations, given what is already known about immigration and clutch size in the system.

2. Results presented in Figure 3

   There are flagged as a major results in the abstract and discussion. However, there is not enough information in the methods to access how this was done, and whether and how the uncertainty was propagated, and so how much weight can be put into these results.

   First, it seems that the lack strong correspondence between the annual prediction from the breeders equation (hard to estimate because of large uncertainty in both heritability and selection differential) and the estimated genetic response (from estimates derived from an animal model with high uncertainty), in non-experimental years where selection seems to be stochastically fluctuating around 0 (and appears not to have been shown to be different from zero), leads to the conclusion that the breeders equation doesn't work. From eyeballing the graph, most of the credible intervals seems to overlap the 1:1 line. For me, this figure provides pretty compelling evidence that the breeders equation does a pretty good job!

   Second, something about the results in Figure 3b screams of regression to the mean, although I am happy to be convinced otherwise. The results here do not appear to explained anywhere in the methods, so I do not know whether uncertainty is taken into account. What worries me here is that there is higher uncertainty in the western populations (presumably due to smaller population sizes), and so more spread at the negative quarter of figure 3b than in the positive quarter (more dominated by eastern populations). This sampling variance creates the appearance of heteroscedasticity, which would lead to two different slopes being estimated when splitting the data by whether it is above or below the 1:1 line.

   Finally, it is worth noting that, although I understand the reason for this approach, the fitness metric used goes from recruit to recruits, and so represents a 'mixed' fitness measure (see Thomson & Hadfield 2017 MEE), which can be problematic. There is less risk using this as clutch size is is a sex limited trait, but it would be worth discussing the potential issues with using this measure (as explain in Thomson & Hadfield 2017 MEE). The fact that there is still a good correspondence between predicted and observed response despite using a potentially problematic fitness measure therefore seems even

more in the favour of the breeders equation doing an OK job, rather than a bad one.

3. In neither this, or the earlier paper (Postma et al 2007), is there a mention of how the selection experiment affected population density. Perhaps I am wrong, but it seems likely that by allowing only around half the birds to breed, you would affect population size and so lower the breeding density of the birds quite fast, and this might additionally prompt more immigration. Whilst I realise that there is a lot of stochasticity in this, it is interesting that in Figure S2 the particularly high years for immigrants in the West occur during the experimental period. Given that the most interesting results in this paper seem to come from a shift in immigration across time, I feel this needs to be addressed. It would useful to at least include some figures showing the *numbers* of locally recruited breeders, island breeders, and mainland breeders in both populations over the time period.

4. In an animal model, unknown immigrants (or founders), are sampled with a mean breeding value of 0 and variance V_A. If they don't have multiple female recruits in the population (i.e. phenotyped descents), then there is no (or very little if they have only one female offspring, or female grand-offspring etc) information about their deviation from 0. As I imagine many breeders have no female recruits, this would mean that there are a large bunch of immigrants that by definition have a mean breeding value of 0. Although it is not clear what kind of proportion that might be, it seems significant for many of the results. For example, Figure 5 shows that breeders with no known grandparents have a mean breeding value of 0, and Figure S3 shows that the mean immigrant breeding value fluctuates closely around 0 and doesn't change over time - I think this is likely simply a consequence of the assumptions of an animal model.

   It would be helpful if there was some exploration of this, and some assurance that the impact of immigrants on the mean breeding value of a population isn't just because a load of individual with no information about the breeding value join the population, and essentially cause regression to the mean. In a similar vein, I think it would be very useful to distinguish between immigrants from the other island population (with parentage information), and unknown immigrants (mainly from the mainland?). For example, is the return from selected clutch sizes during the post-experimental phase due to immigration from the mainland, or immigration between island populations?

   This might be due to a lack of knowledge about modelling multiple populations in the same animal model, but I am also slightly uneasy that the breeding values from both populations converge upon zero in the last decade, where it seems like there would

be the most information. From discussing this with a colleague, I think it would be appropriate to use genetic group models here, with groups for west, east and immigrant populations, and allow heterogeneous variances across them. This would seem prudent given that, in the early years at least, the east and west populations seem quite distinct, and there is clearly an impact of immigrants arriving with potentially different bedding values.

Within this context, it would also be useful to have more information about how the pedigrees form east and west overlap, in other words how much migration is there between populations, and so how linked are the two pedigrees? I feel a little out of my depth here, but I would worry if they don't overlap at all, in terms of making inferences from the same model. Having looked at the earlier papers, its seems that this is a reasonable amount (around 10%), but it would be helpful to clarify here.

Specific Comments:

Abstract

5. l 15 - 'To resolve this' - perhaps 'address' would be more suitable, I don't think this study can claim to single handedly resolve the paradox.

Intro

6. l 28-38 Very nicely written paragraph!

7. l 42 'Besides the stochastic element introduced by genetic drift, immigrants are likely to be genetically distinct from their host population, such that gene flow will also shape a population's genetic characteristics.'

   This seems a bold claim, so I think this needs a reference. Whilst I think it is completely plausible that immigrants are genetically distinct, it really depends on the structure of the wider population. In mainland tit populations for example, I find this relatively hard to believe, a priori.

8. l 43 'Indeed, even very occasional arrivals can be highly influential on the host population's evolutionary trajectory [17,18]'

   True in these examples, but these are very small island populations.

9. l 45 - 'immigration rates often covary' - perhaps 'can covary', I'm not sure enough work has been done on this to say its common?

10. l 58 ‘Despite ongoing gene flow, both genotyping [36] and quantitative genetic modelling [35] has shown that breeders in western and eastern areas are genetically distinct.’

    The introduction needs more of a summary of previous papers on this system. For example, it has previously been shown that differential immigration into the west from the mainland has seemingly led to this difference.

11. l 67 ‘assess the predictability of pre- and post-perturbation adaptation’ and ‘local adaptive processes’

    I don’t think there is any evidence of adaptation here?

Results

12. l 71-81 It would seem prudent when discussing the pre period, to discuss the selection regimes. From Figure 2, it seems as thought there is little overall selection in either east of west, with it fluctuating around 0. Figure 2 doesn’t show any error on these estimates, so it is difficult to assess this. I think a random regression approach, showing whether there is any overall selection, and whether there is variation in selection across years in the two populations would be helpful. It would also be interesting to know whether there was stabilising selection acting on these two populations. If the difference was due to local adaptation, I would expect that either there would be fluctuation in selection with no overall directional selection (indicative of being at an optima - which could equally be estimated with a quadratic function) or directional selection in opposite directions.

13. l 74 I think it is interesting and worth noting that around 80% of the difference between populations appears to be environmental. The wording makes it sounds like the genetic difference was the major driver.

14. l 79-81 This could be more clearly explained, it took me a couple of goes to get it.

15. l 84 ‘when natural selection operated without experimental interference’

    There doesn’t seem clear evidence that there was any natural selection!

16. In l 86, the ‘adaptationist expectation’ is mentioned. However, at this point from looking at figure 2 (and based on Postma & van Noordwijk 2005), I have no expectation that this is adaptation, as there generally appears to be no differences in selection in either population.

17. l 113 $P(b_{fast} > b_{slow})$

In the first paragraph of the results there is just posteriors and credible intervals, then in the second paragraph there is frequentist p values, and then here there is posterior probabilities. These are essentially 1-pvalue, so I think it would be worth explaining this, as I was caught of guard, and thought this was wrong, and I think so would other readers.

18. l 105 As outlined in a comment above, I am sceptical of this, perhaps mainly because of the lack of explanation of the methods used. It feels like stats on stats on stats, and I hope the uncertainty has been appropriately propagated all the way through.

19. l 131 I think the distinction between unknown immigrants and 'local' immigrants from the other island population would be helpful, and might reveal interesting patterns.

20. l 142-143 The posterior probabilities are presumably calculated as the proportion of iterations. I wonder if it would be worth presenting quantile based credible intervals instead of HPD intervals to make them comparable? I imagine it doesn't make much difference, just maybe slightly more consistent.

21. l 149-153 This is a very hard sentence, I would suggest rephrasing.

22. l 156 'first indication' - apart from two papers showing differential immigration into the two areas, in a pattern that would predict the observed difference!

23. l 176 - this is the first mention in the manuscript of the fact that immigration comes form the mainland population, and in fact the only mention of this in the whole manuscript. I think this shift in the pattern of immigration is a really key point in this study, with the potential to be driving all of the results.

24. l 179 - As said above, I think this has to be true due to how the animal model works.

Discussion

25. l 189 'In an attempt to resolve the paradox of stasis [7,9], we assess how consistently the breeder's equation— which has proven very reliable for guiding artificially selected evolution of captive [41] and free-living [30,42] populations—fails to predict adaptive trait evolution across a nearly half-centurial study of two, neighbouring, breeding populations.'

    I disagree that this study shows that the breeders equation doesn't work. In the period of strong artificial selection, the is strong concordance between predicted and observed response, but in other years, where there is no demonstrable selection, it doesn't do

well at picking up response. Personally, I don't think this sheds light on the utility of the breeders equation in the wild.

26. l 196 'Thus, if efforts to quantify adaptive responses are made only when traits are a priori known to be under strong selection, then the apparent ubiquity of the paradox of stasis is attributable to observational selection bias'

    I'm not sure I get this. So the argument here is that in a trait under no clear directional selection, where immigration dominates, we can find evidence for what resolves the paradox of stasis over/as much as in populations/traits where there clear strong, persistent directional selection but no response?

27. l 211 'we show that the genetic impact of immigration is central to understanding contemporary evolution of a key life-history trait, being at least as impactful as natural selection'

    In this example! Whilst this might well be true in other cases, I think care should be taken with generalisation

28. l 227 'relative isolation does not nullify the evolutionary impact of immigration on traits' evolutionary trajectories'

    I would argue that relative isolation was likely to massively amplify the evolutionary impact of immigrants, as the two populations are more likely to be genetically distinct. In a very panmitic situation, I would expect immigration to have much lower impacts, as the genetic differentiation between populations was lower.

29. l 231 - I completely agree

Methods

Generally I think a lot more information if needed throughout the methods to make this easier to follow.

Study system

30. What proportion of the island population is caught every year? Do the nest boxes in the woodland cover the majority of the population? What I am really getting at here is that are 'unknown' individuals assumed to be all from the mainland, or is there a substantial unringed island population.

31. I think there needs to be more discussion of the second clutches. What proportion

produce second clutches? How much are these contributing to recruitment (and so fitness as measured here)? Are the recruits in the analysis just from first clutches? Does the likelihood of second clutches differ between populations? Did the selection regimes affect second clutches?

32. l 257 - I agree that it is unlikely, but presumably you could check whether this had carry over effects on the following years reproduction?

Quantifying Va

33. l 283 - in reference to an comment above - about a third of individuals are founders. It would be good to know how many of the phenotyped ‘founder’ females had females recruits.
34. l 288 there is a rogue brackets towards the end of the line.
35. l 300 Thank you for including some kind of sensitivity analysis
36. l 301 ‘A thinning interval of 10,000 correlations among successive samples of <0.03 for all random effects’ I think there is a missing word somewhere

Quantifying evolutionary change

37. Should the means be weighed by SE or 1/N within each regression? I don’t know how much population size varies across years?

Quantifying selection

38. As mentioned in another comment above, this is a recruit-recruit measure of fitness which mixes fitness of parent and offspring. It would be good to acknowledge the potential issues this can cause in terms of estimation of selection (see Thomson and Hadfield 2017 MEE). For example, maternal clutch size might well indirectly affect offspring survival as it affects juvenile size.
39. l 318-328 - This could be much clearer, and I think these results and a much clearer methodology should be presented in the supplements. It is not clear what models were run. If individual linear regressions on relative fitness (relativised by dividing by the mean), then I am not surprised that sometime disruptive selection was found, as there will be clear non-linearity in the fitness function, due to the limit at 0, which will appear like disruptive selection. This could also cloud stabilising selection. Something like a Poisson GLM seems much better suited for this, as it accounts for this inherent

non-linearity. It would also be very informative to do a random regression analysis here, looking for the overall direction of selection in each population (possibly per period) and whether it differs between populations (as an interaction), and whether there is detectable variation in selection across years. There is an implicit assumption in the breeders equation analysis that directional selection varies across years, but this never seems to be tested. This cannot be assessed from Figure 2, as there is no uncertainty plotted, and if they are estimates from individual models, then the variance among them will be inflated.

Predicting and inferring local adaptation and Figure 3

40. There is unfortunately not enough information presented here to assess the methodology. It is not clear a) whether uncertainty in selection was quantified and incorporated into the uncertainty on the annual predictions of evolutionary change b) whether the uncertainty in both the annual predictions and the inferred responses was incorporated into the regression (e.g. in a bivariate meta-analysis), c) how the analysis in in Figure 3b was done, as it is not even mentioned in the methods - was this a single model, testing the interaction with the uncertainty incorporated? If not, my concern is that this result is due to sampling variance.

41. It is worth noting that the sensitivity analysis with varying h2 per year doesn't allow the Va to change per year - that is fixed in the animal model - it is essentially allowing the environmental component to vary. So whilst this is a useful additional, it might also be that there is GxE, which this doesn't test.

42. Very minor point, but the 'CS' subscript on the S seems unnecessary sine there is only selection on clutch size. When I first read it I thought it was an index.

Decomposing evolutionary change

43. l 348 ' democratic' - presumably demographic?

44. I find this part hard to follow, and still can't work out whether the logic of l 375-381 works, or whether the resulting sum of components will give back the correct change. The contribution to a current years mean can also be thought of as the weighted averages of the different groups. It would seem that this much much easier to calculate and explain:

$$\bar{a} = p_{survivors}\bar{a}_{survivors} + p_{recruits}\bar{a}_{recruits} + p_{immigrants}\bar{a}_{immigrants}$$

where p is the proportion. The contribution of group x can then just be $p_x\bar{a}_x$. Perhaps there is a reason why this wouldn't work and the other method would, but as it stands I don't understand the current method!

45. It would be interesting to also separate to within island from mainland immigration.

46. l 369-373 The relevance of this information for this section is not clear to me.

47. Figure 5 is never described in the methods, and only referred to in passing in the results.

Data Availability

48. Data and code doesn't seem to be available for review (at least the provided DOI didn't work for me). I think this is a shame, as it would have helped me work out what was done in several places where the methods were unclear (especially in the selection analyses), which would save a bit of back and forth in review.
