# Supplementary material for: Gene flow drives periods of both evolutionary stasis and change in a wild bird population
Source: PLoS Biol. 2026 Sep 21;24(9):e3004004. doi: 10.1371/journal.pbio.3004004 (PMC13630318; doi:10.1371/journal.pbio.3004004)
Supplement: Supplementary file 13 [file pbio.3004004.s022.pdf]

Thank you for your patience while your manuscript "Paradox lost? Gene flow determines trait evolution in a wild bird population" was peer-reviewed at PLOS Biology. Your manuscript has been evaluated by the PLOS Biology editors, an Academic Editor with relevant expertise, and by three independent reviewers.

You'll see that Reviewer #1 is impressed by the dataset and the analyses but finds the paper to be rather confusing and is ultimately not convinced that the two central claims are fully supported by the data. These concerns centre around definition of fast and slow evolution, assigning early survival to maternal fitness, and evidence for the importance of immigration. Reviewer #2 is again broadly positive, but raises similar concerns about lack of clarity, the framing, and the potential for statistical artefact. In his long review he raises a list of more specific concerns. Reviewer #3 is also positive, but raises several concerns about some inconsistencies between aspects of your analytical framework, some assumptions underlying breeding value prediction, biases arising from immigration, and lack of tests for drift.

IMPORTANT: I discussed these reviews with the Academic Editor, and the following cherry-picked comments might prove helpful when you're revising the paper: "It is clear that all three reviewers think this is a really interesting data set and well worthy of publication by PLoS Biol. I agree. I also have the impression that the long list of suggestions for analytic changes are sensible. Maybe it won't be possible to simultaneously satisfy all three reviewers in every instance - especially when there is a choice of approaches... I'd 'reassure' the authors that in cases where the suggestions of the reviewers conflict it is OK to pick one option, but they should be sure to justify why they did not pursue the alternate approach."

As you will see in the reviewer reports, which can be found at the end of this email, although the reviewers find the work potentially interesting, they have also raised a substantial number of important concerns. Based on their specific comments and following discussion with the Academic Editor, it is clear that a substantial amount of work would be required to meet the criteria for publication in PLOS Biology. However, given our and the reviewer interest in your study, we would be open to inviting a comprehensive revision of the study that thoroughly addresses all the reviewers' comments. Given the extent of revision that would be needed, we cannot make a decision about publication until we have seen the revised manuscript and your response to the reviewers' comments. Your revised manuscript would need to be seen by the reviewers again, but please note that we would not engage them unless their main concerns have been addressed.

In addition to these revisions, you will need to complete some formatting changes, which you will receive in a follow up email. A member of our team will be in touch with a set of requests shortly.

We appreciate that these requests represent a great deal of extra work, and we are willing to relax our standard revision time to allow you 6 months to revise your study. Please email us (plosbiology@plos.org) if you have any questions or concerns, or envision needing a (short) extension.
At this stage, your manuscript remains formally under active consideration at our journal; please notify us by email if you do not intend to submit a revision so that we may withdraw it.

Thank you again for your submission to our journal. We hope that our editorial process has been constructive thus far, and we welcome your feedback at any time. Please don't hesitate to contact us if you have any questions or comments.

Sincerely,

Roli Roberts

Firstly, we thank the editors and reviewers for taking the time to consider our manuscript, and for giving us the opportunity to revise it in light of this feedback. As we hope is clear from the detailed responses below, we have incorporated this feedback into both our analyses and the manuscript, and we believe the outcome is a more robust analysis and a clearer report. Throughout our responses, line numbers refer to the version of the revision without tracked changes.

To give an overview of the most significant changes:

• The foremost change is how the motivation for the study is presented. It was clear from the reviewers' comments that the aims of our study were unclear and the analyses were not as logically connected as they could be. We have attempted to be more explicit about why we feel the study is necessary, for example highlighting that the paradox of stasis, whilst widely discussed, has rarely been subject to objective quantitative assessment (lines 15-16, 41-43). We proceed from this to highlight the near-

universal conflation of adaptation and evolution when comparing expected and inferred genetic change (lines 20-22, 45-55, 151-165, 269-294), which in turn makes quantification of the genetic impact of immigration (which accounts for the disparity between local adaptation and total genetic change) a logical next step. We hope that reviewers will agree that the motivation for our study is now clear, our choice of analysis likewise clear and that the results follow a logical sequence that delivers on the take-home message of our title.

• We conducted a series of alternative analyses relying on the genetic groups animal modelling approach recommended by the reviewers. These and our reasons for ultimately rejecting this approach for our main results are detailed in the Methods (lines 539-572; with supporting data in the Supplementary Materials: Figs. S7-S10) but, in short, we interpret the outcome of these analyses as indicative of a population that lacks a distinctive 'native' ancestry, as we might expect of an island for which breeding habitat is of relatively recent and anthropic origin.

• We have revised our examination of the correspondence of expected and inferred interannual genetic change, having recognised the merit of the criticisms raised by reviewers. We no longer rely on the Breeder's Equation to generate our expectation of interannual genetic change but instead generate year-and-population-specific estimates based on the Price equation (lines 124-165, 426-467).

• We offer greater explanation of how the demographic decomposition is calculated, and simplified our reliance on it by merging the two processes underlying local adaptation so that their net effect can be better contrasted with that of immigrant recruitment (Fig. 5), while keeping the full decomposition in the Supplementary Materials (Table S4, Fig. S3). This change also serves to better unify the two halves of our analyses in the manuscript.

Reviewer #1:

This multi-decadal study of clutch size evolution in nest box populations of great tits addresses two fundamental questions in evolutionary biology. 1) Why do we often see an absence of an evolutionary response to measured natural selection on heritable traits (paradox of stasis)? 2) How important is gene flow relative to other evolutionary processes to overall rates of evolution. There are two foundational, timely and important questions in the field and the latter question in particular is very understudied. The dataset used to address the question is terrific and the analyses are sophisticated. The manuscript and writing are generally clear, but there were a few places where I was confused - either by the complexity of the material being presented or the way in which the material was presented.

Considering all reviewers' feedback, we can see that our manuscript lacked a clear narrative and that the purpose of the analyses was not explained sufficiently. We believe these shortcomings are now rectified.

Overall the study makes two overall claims. First, that an overall correspondence between evolutionary predictions and observations can result from episodes of under- and over-prediction. Second, that gene flow is important to evolutionary dynamics in this system. Unfortunately, I was not convinced by the data supported these two important conclusions.

We have undertaken considerable revision of our analyses and the report in response to these criticisms. This has strengthened our conviction that our conclusions are not only important but also correct, and we hope that there is now sufficient clarity for the reviewers to feel comfortable in supporting us in this.

It seems a certainty that the slope for 'slow' years will be lower when 'slow' defines the range on the y-axis to be more constrained. I think the observed patterns in 3b will always be the case - or at least I would like to see some sort of null model or simulation to asses the degree to which these patterns are pre-determined. It is possible here that I have misunderstood how period of fast and slow evolution were defined. If so, then more clarity is needed here. I also worry that there is a potential confound between the strength of selection associated with the degree of certainty that the appropriate target of selection has been identified. Selection was measured as a selection differential, which can be confounded by indirect selection on other traits. This is more likely in years of 'natural selection' and less likely in years with 'artificial selection'. The latter also appear to have stronger selection differentials. So the strength of selection is likely confounded by the certainty with which clutch size is the target of selection.

We respond to the concerns in this paragraph below.

Overall, the selection differential measures are also likely problematic because selection on the maternal trait is based on a fitness measure that includes offspring early survival. Thomson and Hadfield discuss the risks associated with assigning early survival to the fitness of their mother, particularly for the prediction of rates of evolutionary change. There are circumstances under which these risks are lower, but this needs to be carefully assessed and reported. One of the conditions is that the parental trait does not affect parental survival, but this does not seem to be the case here since breeder mortality seems to be an important driver of changes in clutch size breeding values in Figure 4.

Firstly, we agree with the criticism of our inclusion of experimental years, and these are excluded from our revised analyses (lines 124-165, Fig. 3). Secondly, we agree that our division of observations was difficult to understand, and this has been dropped entirely.

An advantage of quantifying selection as the phenotypic covariance between clutch size and phenotype is that there is a clearly defined expectation for how this will translate to selection on breeding values if selection is unbiased (see Table 2 of Postma 2006, *J. Evol. Biol.*). This provides us with the null model that Reviewer 1 argues for (lines 129-141, 445-458).

I was also unconvinced of the importance of immigration to evolutionary dynamics as shown in Figure 4 and Table S5. I tried very hard, but could not figure out what metrics or items in these depictions led to the inference that immigration is the driver of evolutionary dynamics.

Regarding the reviewer's uncertainty over how we reach our conclusions about the impact of immigration, this is based on assessing the relative sizes (and directions) of the contributions to annual genetic change made by mortality, native recruitment and immigrant recruitment. These contributions sum to the overall genetic change. To better highlight the relative importance of immigration, we have replaced the main-text figure, in which the demographic processes generating local adaptation are combined into a single net effect that is plotted alongside the mean impact of immigration (Fig. 5). We have also revised our description in the text, to better explain the results to the reader (lines 210-230).

We hope that this simplified illustration and the revisions to the text make it clearer how we arrived at our conclusion that immigrant recruitment is a major determinant of our breeding populations' genetic trajectory for clutch size. A figure plotting the distinct contributions of the three demographic processes is retained in the Supplementary Materials (Fig. S3).

I also have worries about the ability of standard animal models to assess the importance of immigration to evolutionary dynamics when they are based on a homogenous base population. Reid and Wolak (and others) have done some elegant work to demonstrate the value of animal models with heterogeneous genetic groups as a way to explicitly model the impacts of gene flow through immigration (one citation in this manuscript; #18). I am not convinced that the current approach, which assumes that immigrants and residents come from a common base population adequately reflects the biology that the authors are attempting to study. I think to explicitly test the importance of gene flow, genetic group models seem necessary to allow immigrants to come from a different base population. This issue ought to be explicitly addressed.

The possibility that the estimated breeding values of immigrants come from a different distribution was a concern common to all reviewers, and we therefore followed their advice in accommodating genetic groups effects in our revised analyses (lines 539-572, Figs. S7-S10). However, we also highlight here that many immigrants into both East and West are of known origin, since immigration is defined at the level of the breeding populations (i.e., East and West), such that dispersal between these—as well as immigration of Village-origin birds—represents immigration. This is illustrated in an additional figure (Fig. S6) but we have also sought to make this clearer throughout the text, including explicit statement (lines 179-180).

Specific Comments

- line 17. I struggled with the statement that evolutionary responses to selection were smaller than predicted in years of evolutionary stasis. Was the relationship between prediction and response not used to define which years involved stasis? Do you simply mean that in some years the rate exceeded predictions and in some years it was lower than predictions? Stasis is perhaps not the right word here because this implies (to me at least) a lack correspondence between observed and predicted and not just the magnitude of either.

This aspect of our study is not included in the revised manuscript.

- lines 301-302 regarding thinning interval did not make sense to me. I wondered whether some words were missing.

Yes, words were missing from this sentence. This is corrected in the revised text (line 386-388).

- how are 'breeders' defined with respect to males? All males in the population at that time?

We have revised the text to make this less ambiguous: references to "breeders" in the main text have now been changed to alternative wording, and when this phrase is used in the Materials and Methods we include clarification that it does indeed refer to both females and males (line 528-530; also legend of Fig. 6).

- How were years of 'fast' versus 'slow' annual responses defined? Were these defined for predicted or observed rates? Were these based on absolute values of rates, or were years of negative evolutionary rates defined to be 'slow' if if it were a rapid evolutionary reduction in clutch size? I assume the former (absolute values), but this was not clear.

We have removed these analyses from our revised manuscript.

- lines 334-338. In terms of notation, I don't think the subscript SC adds clarity since selection was only measured on one trait. The subscript k was included in line 334 but lost in the equation on line 338. Since selection was measured each year, but heritability was measured overall it would be helpful to add subscripts of t and k to R and S to clarify that these were year- and population-specific.

Thank you. We have revised the notation used throughout the manuscript.

- line 121. I don't think this conclusion that flawed measures of selection are to blame for the stasis is supported. There are many possible explanations for the patterns but the absence of year-specific heritabilities does not imply that any of the other remaining explanations are more likely than others.

Thank you for this criticism. This claim has been removed during revision of our manuscript.

-I was unable to understand the section on the calculation of recruits through natives and immigrants (lines 375 - 381).

It's clear that this section of the Methods was insufficient. We have added more detail, including a figure to illustrate the general approach (lines 490-509, Fig. 4).

Reviewer #2

[identifies himself as Joel Pick. IMPORTANT: Please see attachment for fully formatted version]

This study looks at a 44 year dataset of two island populations of great tits, that initially differed in clutch size, a key life history trait. The study charts the changes in breeding values for clutch size through 15 years pre-experiment, then an 8 year artificial selection experiment to reverse this difference, and then for 20 years post artificial selection. This study is done in the context of earlier work on the initial differences between the populations, including the roles of local adaptation and immigration (Postma and Van Noordwijk 2005, Postma et al 2009), and the selection experiment itself (Postma et al 2007). In this study, the authors seek to attribute the differences and changes in breeding values between populations across these time periods to the relative influences of selection and immigration. This study uses a remarkable dataset, to address an interesting, timely, and largely ignored question in wild quantitative genetics.

Generally the writing style is very clear and easy to follow, although in several places I felt I needed more information, and particular parts of the method need clarification. I also felt it could have been put more firmly in the context of the previous work, which already established the important role of immigration in this population. I don't think that this previous work diminishes the current contribution, but not knowing the previous work well meant that part of the results were hard to contextualise.

I have some major comments that are maybe due to my own ignorance or misunderstanding, but could at least do with some additional discussion in the manuscript. In particular, as there is a lot of complex analysis, which often using estimates from several different models, there is opportunity for patterns to arise due to statistical artefacts - it would be nice to be reassured that this wasn't the case, or what the potential for these artefacts to arise was. I apologise for the length of the review, but this is the most interesting manuscript I have reviewed in a long while.

Major Comments

1. Overall framing

This is a very lengthy comment, so we offer responses to sections, rather than attempting to respond to the entire comment.

This is clearly up to the authors, but I find the overall framing a little confusing. I have a bit of a stumbling block over how the paradox of stasis fits in here. It seems like the starting point/framing of the manuscript is selection/local adaptation, and why we don't find change in the presence of strong, persistent directional selection (paradox of stasis). Whilst I would a priori think that clutch size was under strong, persistent directional selection as it is a component of fitness (although various previous studies have suggested that it is under stabilising selection in tit populations), it doesn't appear to be under clear selection in these populations at all (see also Postma and Van Noordwijk 2005). To my mind, for there to be local adaptation in two close populations, there needs to be evidence of differing optima, or consistently different directional selection regimes in the two populations. Based on figure two and table S2 (and Postma and Van Noordwijk 2005), there doesn't seem to be clear evidence of directional selection on clutch size in either the same or opposite directions in the pre-experimental period. From the limited description on l318-328, it also seems like there is no clear stabilising selection, and no mention of differing optima between populations.

Considering all the reviews, we recognise that our manuscript's ambition was unclear. We believe that the revised manuscript addresses this, offering a clearer explanation of the need for this study. Note that the opening paragraph of the Main Text, which sets out its broad context, makes no allusion to strong or persistent selection (lines 31-43). Rather, it highlights that expectations are seldom met, compelling evolutionary ecologists to characterise this situation as a paradox.

We fully agree with the reviewer's expectation that clutch size would be subject to strong selection. However, we note that our demographic decomposition suggests that, at least during the pre-perturbation period, it may have been, at least in the East and at a genetic level. As detailed in John Endler's iconic book, *Natural Selection in the Wild* (pp. 75-81), evolutionary perturbation is a way of forcing natural selection to show its hand. We also note that weak selection via interannual genetic success may be disguising the presence of strong selection via components of annual fitness. This is conjectural here and would not invalidate our analyses, but is the subject of a separate manuscript.

However, the reviewer conflates *within*- and *between*-population differences. As the reviewer later highlights, the genetic difference in clutch size between West and East is attributed to both a difference in the rate of immigration from outside the nestbox study and the reproductive success of immigrants' descendants. This distinction can be sustained for a range of selective scenarios with respect to the two breeding populations, with the extent to which it is attributable to immigration patterns dependent on the relative strength of local adaptation and the relative frequency of immigration. This is a central focus of our manuscript, which concerns *within*-population evolution (with the exception that it addresses the conspicuous failure to return to the pre-experimental genetic distinction).

The previous work on this population seems to show that local adaptation isn't the reason for the pre-experimental difference between populations, rather it is due to differential immigration from the mainland into the two island populations, with the mainland immigrants having larger eggs than the island birds, resulting in a larger egg size in the West population with greater immigration (Postma and Van Noordwijk 2005). This information seems lacking from the introduction, results and discussion, although a key bit of context for the framing of the study.

If we are then not looking at a trait that is under strong, persistent directional selection, or with clear evidence of divergent optima between populations, it seems strange to phrase this so heavily in the context of the paradox of stasis. I'm not sure that this study, although a valuable demonstration of the role of migration and gene flow in microevolution on small spatial scales, tells us anything about resolving the paradox of stasis, as it seems in this case we would predict stasis through the lack of selection. Or at least, if it does, I think it needs far better signposting to these results.

We hope that both our revised manuscript and this document clarify the need for an objective assessment of the paradox of stasis, rather than reliance on *a posteriori* evocation to determine its prevalence and severity. Our revised analyses and our reporting of them offer a clearer, simpler and quantitative assessment of the whether adaptation is consistently less than expected.

The question may then be what happens when we place a trait under strong selection in the wild, and how do different processes (such as immigration) affect selection response. During the period of artificial selection, there was a response in the breeding values (Postma et al 2007, and this study), which shows that strong directional selection in these populations would have a response. When the selection stops the response is then swamped by immigration. This is phrased as a 'paradox of flux' (l 98), but I am not clear as to why there is a paradox. Given the generally high immigration rates in tit populations (and in this population, shown Figure S3), is it surprising that immigration is a major driver of return to a previous state after selection ends? I'm just thinking here that, theoretically, if I took two open subpopulations, and performed an artificial selection experiment on a selectively neutral trait in opposing directions, then given immigration rates of 20-50% (figure S3), I would expect a rapid return to mean trait value when selection stopped. If the point is that this is what we would expect, but this is not typically considered by researchers (with which I totally agree), then I think that the introduction should lean on this much more and articulate this prediction, rather than this result being surprising ('paradox of flux').

We believe our revised manuscript better clarifies that we can examine the response to selection exclusive of immigration (e.g., lines 124-157); indeed, that we should be doing so (yet seldom do) is perhaps the principal conclusion that our study offers. As we demonstrate, failure to accommodate the distinction between evolution and local adaptation generates an apparent paradox of stasis. Thus, were we to be consistent in applying this flawed approach, we would recognise the clutch size evolution of the post-experimental period as a paradox of flux (characterised as a 'paradox of change' in our revised manuscript, for simplicity: lines 118, 273). The reviewer objects to this characterisation on the basis that it is attributable to immigration yet is content to defend a paradox of stasis that is identified based on this very same flaw. Our manuscript explicitly points out this flaw, using a long-term quantitative genetic study of clutch size to demonstrate that a paradox of stasis is, at minimum, greatly exaggerated by the failure to isolate local adaptation from post-selection immigration.

I am not saying that an experimental demonstration of this effect is not very important, interesting and useful, I'm just wondering whether, giving the existing knowledge of the system, would anything else have been expected. The impression I get from the manuscript is that this is somehow unexpected. The paradox of stasis, for my perspective, relates to traits under strong, persistent directional selection, for example juvenile body size.

For me then, it is not super clear how this study shows that immigration is working against selection in this population. Upon reading this several times, I think that a key paragraph is l145-157, but I don't think that the importance of this results is clearly signposted, or put well into the context of the previous work on the system.

We have revised the main illustration accompanying the demographic decomposition of genetic change (now Figure 5) so that it contrasts the impact of immigrant recruitment with the net impact of the *in situ* processes underlying local adaptation. This shows immigration and local adaption to be frequently acting in opposition, although this can also be inferred from our assessment of the predictability of interannual genetic change, since this shows that failure to account for immigration reduces the apparent response to selection via local adaptation (lines 124-157).

Here the demographic decomposition shows a reasonable impact of immigration in the western population, but not the eastern, during the experimental period. I think this would be expected, given the previous work on immigrations into the west and not the east, but this is not clearly articulated. There is then the effect of immigration in the legacy period in the east population, but these effects seem relatively small and uncertain, and it is difficult to separate the effects of drift (although there does seem to be phenotypic selection for larger clutches here, but I don't think that this is statistically demonstrated anywhere).

The Main Text of our revised manuscript retains the explicit statement that the selection experiment was designed to act via selective recruitment of local-origin individuals (now lines 185-186). It also explicitly notes that the demographic decomposition will capture the effects of drift, since it does not directly quantify *evolutionary* processes but rather the genetic (~evolutionary) impact of *demographic* processes (lines 180-183).

Again the framing is up to the authors (and I am happy to be shown wrong), but my suggestion would be to put this much more firmly into the context of the previous work, and make predictions on how you would expect immigration and selection to balance each other in the two populations, given what is already known about immigration and clutch size in the system.

Through both our responses to their comments and our revised manuscript, we have clarified the need for this study and its value as it was structured, in which we first assess the reliability of evolutionary predictions by conducting an objective assessment, correct the conflation of adaptation and evolution that is almost universal in studies of contemporary evolution in the wild, and then quantify the relative importance of local adaptation versus immigration in driving evolutionary change.

2. Results presented in Figure 3

There are flagged as a major results in the abstract and discussion. However, there is not enough information in the methods to access how this was done, and whether and how the uncertainty was propagated, and so how much weight can be put into these results.

We have revised the analyses that this figure illustrates and clarify how we propagate the applicable errors (lines 377-379, 388-389).

First, it seems that the lack strong correspondence between the annual prediction from the breeders equation (hard to estimate because of large uncertainty in both heritability and selection differential) and the estimated genetic response (from estimates derived from an animal model with high uncertainty), in non-experimental years where selection seems to be stochastically fluctuating around 0 (and appears not to have been shown to be different from zero), leads to the conclusion that the breeders equation doesn't work. From eyeballing the graph, most of the credible intervals seems to overlap the 1:1 line. For me, this figure provides pretty compelling evidence that the breeders equation does a pretty good job!

In revising our analyses and the manuscript, we have not only rectified the weaknesses of our reliance on the breeder's equation but sought to make our approach easier for readers to understand. As before, annual-level estimates of selection are used to generate predictions of adaptive change and our revised analysis more clearly demonstrates how and why they fail.

Second, something about the results in Figure 3b screams of regression to the mean, although I am happy to be convinced otherwise. The results here do not appear to explained anywhere in the methods, so I do not know whether uncertainty is taken into account. What worries me here is that there is higher uncertainty in the western populations (presumably due to smaller population sizes), and so more spread at the negative quarter of figure 3b than in the positive quarter (more dominated by eastern populations). This sampling variance creates the appearance of heteroscedasticity, which would lead to two different slopes being estimated when splitting the data by whether it is above or below the 1:1 line. Finally, it is worth noting that, although I understand the reason for this approach, the fitness metric used goes from recruit to recruits, and so represents a 'mixed' fitness measure (see Thomson & Hadfield 2017 MEE), which can be problematic. There is less risk using this

as clutch size is is a sex limited trait, but it would be worth discussing the potential issues with using this measure (as explain in Thomson & Hadfield 2017 MEE). The fact that there is still a good correspondence between predicted and observed response despite using a potentially problematic fitness measure therefore seems even more in the favour of the breeders equation doing an OK job, rather than a bad one.

We have removed these analyses from our revised manuscript.

3. In neither this, or the earlier paper (Postma et al 2007), is there a mention of how the selection experiment affected population density. Perhaps I am wrong, but it seems likely that by allowing only around half the birds to breed, you would affect population size and so lower the breeding density of the birds quite fast, and this might additionally prompt more immigration. Whilst I realise that there is a lot of stochasticity in this, it is interesting that in Figure S2 the particularly high years for immigrants in the West occur during the experimental period. Given that the most interesting results in this paper seem to come from a shift in immigration across time, I feel this needs to be addressed. It would useful to at least include some figures showing the NUMBERS of locally recruited breeders, island breeders, and mainland breeders in both populations over the time period.

The requested figure is now provided in the Supplementary Materials (Fig. S6). Reviewer 2 is correct to highlight that intense artificial selection will unavoidably influence the population dynamics, as was discussed by Postma et al. (2007, *J. Evol. Biol.*). To some extent, the negative impact on fledgling production was offset by transferring confiscated clutches between the two breeding populations. However, this was only done for ~50% of removed clutches. However, Figure S6 (*a*) supports our assertion that this influence ceased with the end of the selection experiment, and (*b*) suggests that any impact on the sizes of the breeding populations was similarly short-lived, such that the influence on the relative frequency of immigrant recruits during the post-experimental period seems limited.

4. In an animal model, unknown immigrants (or founders), are sampled with a mean breeding value of 0 and variance VA. If they don't have multiple female recruits in the population (i.e. phenotyped descents), then there is no (or very little if they have only one female offspring, or female grand-offspring etc) information about their deviation from 0. As I imagine many breeders have no female recruits, this would mean that there are a large bunch of immigrants that by definition have a mean breeding value of 0. Although it is not clear what kind of proportion that might be, it seems significant for many of the results. For example, Figure 5 shows that breeders with no known grandparents have a mean breeding value of 0, and Figure S3 shows that the mean immigrant breeding value fluctuates closely around 0 and doesn't change over time – I think this is likely simply a consequence of the assumptions of an animal model.

It would be helpful if there was some exploration of this, and some assurance that the impact of immigrants on the mean breeding value of a population isn't just because a load of individual with no information about the breeding value join the population, and essentially cause regression to the mean. In a similar vein, I think it would be very useful to distinguish between immigrants from the other island population (with parentage information), and unknown immigrants (mainly from the mainland?). For example, is the return from selected clutch sizes during the post-experimental phase due to immigration from the mainland, or immigration between island populations?

As discussed in our response above, the Supplementary Materials now includes a figure providing a demographic breakdown of each year's annual breeding population (Fig. S6). While we have extensively explored the value of animal models that explicitly recognise the source population of unknown-origin recruits as being genetically distinct from the founders of the island populations (lines 539-572; Figs. S7-S11), we highlight that such individuals "with no information about [their] breeding value" can make only a very weak contribution via such a regression-to-the-mean phenomenon, precisely by dint of the large uncertainty in their breeding values. In practice, these would have to be males, since recruited unknown-origin females in our sample will have at least one observed clutch size. A potential lack of phenotyped relatives presents potential biases but (*a*) this is not particular to our study, and (*b*) these individuals' breeding values are expected to have a broader posterior probability distribution (uncertainty that is propagated through our analyses).

This might be due to a lack of knowledge about modelling multiple populations in the same animal model, but I am also slightly uneasy that the breeding values from both populations converge upon zero in the last decade, where it seems like there would be the most information. From discussing this with a colleague, I think it would be appropriate to use genetic group models here, with groups for west, east and immigrant populations, and allow heterogeneous variances across them. This would seem prudent given that, in the early years at least, the east and west populations seem quite distinct, and there is clearly an impact of immigrants arriving with potentially different bedding values.

We have conducted extensive modelling using alternatively structured animal models that explicitly recognise out-of-study immigrants as coming from a genetically distinct source population. These are detailed in our revised manuscript (lines 539-572; Figs. S7-S11) and in response to the previous comment, so we simply highlight that our interpretation of the results of these additional analyses is that genetic differences between contemporary island-origin breeders and recruited immigrants are of recent origin, and thus will be captured by the population pedigree.

Within this context, it would also be useful to have more information about how the pedigrees form east and west overlap, in other words how much migration is there between populations, and so how linked are the two pedigrees? I feel a little out of my depth here, but I would worry if they don't overlap at all, in terms of making inferences from the same model. Having looked at the earlier papers, its seems that this is a reasonable amount (around 10%), but it would be helpful to clarify here.

This information is provided in the figure illustrating the demographic composition of each year's breeding population (Fig. S6). As shown, there is frequent and continuous dispersal between the two populations. We reflect this in considering West and East to have a common ancestral population.

Specific Comments:

Abstract

5. l 15 - 'To resolve this' - perhaps 'address' would be more suitable, I don't think this study can claim to single handedly resolve the paradox.

Agreed. We have removed this wording as part of a more substantial revision of the abstract.

Intro

6. l 28-38 Very nicely written paragraph!

Thank you. This paragraph has not been heavily revised, beyond addition of a sentence at its end (lines 41-43).

7. l 42 'Besides the stochastic element introduced by genetic drift, immigrants are likely to be genetically distinct from their host population, such that gene flow will also shape a population's genetic characteristics.'

This seems a bold claim, so I think this needs a reference. Whilst I think it is completely plausible that immigrants are genetically distinct, it really depends on the structure of the wider population. In mainland tit populations for example, I find this relatively hard to believe, a priori.

We disagree: immigrants come from a population that has a distinct demographic history and is subject to distinct environmental conditions. Given that genetic drift will also be acting, there are both stochastic and deterministic processes that will give rise to genetic differences between immigrants' natal and post-dispersal populations. We can, of course, debate the nature and magnitude of these differences, as well as whether they're detectable, but that they should be absent seems extremely unlikely. Furthermore, the reviewer elsewhere argues that genetic groups should be explicitly modelled, which is at odds with their assertion here.

We also remind the reviewer that migration is considered at a within-island level, as our revised manuscript states more clearly (e.g., lines 179-180). As they note elsewhere in this review, prior work has established a genetic distinction between our two focal (sub)populations of nestbox-breeding great tits (as detailed in our Introduction: lines 61-64).

8. l 43 'Indeed, even very occasional arrivals can be highly influential on the host population's evolutionary trajectory [17,18]'

True in these examples, but these are very small island populations.

Yes. But then larger populations will tend to receive greater numbers of immigrants. There are many well-documented examples of introgression playing a significant role in the evolutionary trajectory (and success) of populations.

9. l 45 - ‘immigration rates often covary’ - perhaps ‘can covary’, I’m not sure enough work has been done on this to say its common?

We argue that “often” is already a concession to the—in our view, unlikely—possibility that immigration rates are unrelated to local mortality, so prefer to retain our original text (lines 49-50).

10. l 58 ‘Despite ongoing gene flow, both genotyping [36] and quantitative genetic modelling [35] has shown that breeders in western and eastern areas are genetically distinct.’

The introduction needs more of a summary of previous papers on this system. For example, it has previously been shown that differential immigration into the west from the mainland has seemingly led to this difference.

Previous work examines the difference between the two breeding populations. While this difference is unarguably part of the narrative of our study, its focus is on the evolutionary change occurring *within* the two breeding populations. The paragraph introducing our study system (now lines 57-71) now ends with the statement that we are examining “…*interannual genetic change within each*” (line 71).

11. l 67 ‘assess the predictability of pre- and post-perturbation adaptation’ and ‘local adaptive processes’

I don’t think there is any evidence of adaptation here?

This seems a good place to make the important clarification that we don’t consider adaptation to be a process that is either present or absent. Rather, it will — as with drift — always be operating to some extent, even if this is not detectable.

We hope that by making explicit the lack of objective assessments of the paradox of stasis, we help the reader better understand our motivations: testing the reliability of evolutionary predictions only in situations where natural selection is already known to be strong, as the reviewer advocates, has clear potential to introduce severe observation selection bias.

Results

12. l 71-81 It would seem prudent when discussing the pre period, to discuss the selection regimes. From Figure 2, it seems as thought there is little overall selection in either east of west, with it fluctuating around 0. Figure 2 doesn’t show any error on these estimates, so it is difficult to assess this. I think a random regression approach, showing whether there is any overall selection, and whether there is variation in selection across years in the two populations would be helpful. It would also be interesting to know whether there was stabilising selection acting on these two populations. If the difference was due to local adaptation, I would expect that either there would be fluctuation in selection with no overall directional selection (indicative of being at an optima - which could equally be estimated with a quadratic function) or directional selection in opposite directions.

We understand the reviewer’s inclination to focus on the long-term central tendency of directional selection on clutch size. However, our focus is on the explicability of local adaptation, which requires quantification of selection over the focal time-period (in our case, one year). As other responses note, we reject the implication that evolutionary prediction is invalidated by weak selection. We revised the manuscript, in part, with a view to better communicating this focus to the reader.

13. l 74 I think it is interesting and worth noting that around 80% of the difference between populations appears to be environmental. The wording makes it sounds like the genetic difference was the major driver.

We have reworded this sentence (line 81-83).

14. l 79-81 This could be more clearly explained, it took me a couple of goes to get it.

Agreed. We have simplified this sentence (lines 85-88).

15. l 84 ‘when natural selection operated without experimental interference’

There doesn’t seem clear evidence that there was any natural selection!

As with our response to comment #11, we feel the Reviewer adopts an absolutist stance to characterising natural selection that is flawed in both its realism and its implications for how we assess the performance of evolutionary predictions.

16. In l 86, the 'adaptationist expectation' is mentioned. However, at this point from looking at figure 2 (and based on Postma & van Noordwijk 2005), I have no expectation that this is adaptation, as there generally appears to be no differences in selection in either population.

This sentence (reworded slightly; lines 104-107) presents the adaptationist explanation for the inferred (partial) evolutionary recovery of the *status quo ante*. We simply present an interpretation that is frequently made when—explicitly or, very frequently, implicitly and without awareness—genetic change is quantified.

17. l 113 P (bfast > bslow)

In the first paragraph of the results there is just posteriors and credible intervals, then in the second paragraph there is frequentist p values, and then here there is posterior probabilities. These are essentially 1-pvalue, so I think it would be worth explaining this, as I was caught of guard, and thought this was wrong, and I think so would other readers.

As advised, we have added clarification that quoted value such as these are posterior probabilities (lines 192-193).

18. l 105 As outlined in a comment above, I am sceptical of this, perhaps mainly because of the lack of explanation of the methods used. It feels like stats on stats on stats, and I hope the uncertainty has been appropriately propagated all the way through.

We no longer rely on these analyses but note that the manuscript clarifies that uncertainty in BLUP estimation is propagated through all downstream analyses (lines 377-381, 510-512).

19. l 131 I think the distinction between unknown immigrants and 'local' immigrants from the other island population would be helpful, and might reveal interesting patterns.

We agree. However, as our revised manuscript explains, partitioning the incoming (new recruits) and outgoing (breeders suffering mortality) demographics introduces uncertainty, unless you are confident about the temporal sequence of the sub-groups (lines 494-498). Additionally, we treat the two breeding populations as each being representative of songbird study populations more broadly, for which such distinctions are usually impossible.

20. l 142-143 The posterior probabilities are presumably calculated as the proportion of iterations. I wonder if it would be worth presenting quantile based credible intervals instead of HPD intervals to make them comparable? I imagine it doesn't make much difference, just maybe slightly more consistent.

As it is not clear how the first and the second part of the reviewer's comment relate to each other, and we agree that it will not make a difference and at best makes the reporting slightly more consistent, we have not changed how we report our results. We also note that the posterior probabilities (for example the proportion of the posterior distribution that is above 0) and the HPD intervals (for example for variance component estimates) aim to convey different information.

21. l 149-153 This is a very hard sentence, I would suggest rephrasing.

Thank you, we have revised this sentence (lines 199-204).

22. l 156 'first indication' - apart from two papers showing differential immigration into the two areas, in a pattern that would predict the observed difference!

We had meant "first indication" within the context of our results. We hope the revised sentence is acceptable (lines 207-208).

23. l 176 - this is the first mention in the manuscript of the fact that immigration comes form the mainland population, and in fact the only mention of this in the whole manuscript. I think this shift in the pattern of

immigration is a really key point in this study, with the potential to be driving all of the results.

We have revised this paragraph, expanding our explanation of the changes that have occurred over the study period (lines 239-268). We have also attempted to better communicate that—until this final section of analysis—immigration is considered at the level of breeding population (i.e., West or East).

It seems to us an exaggeration to attribute all results to changes in the pattern of immigration. The lack of correspondence between expected and inferred interannual genetic change that is the focus of the first part of the Results is not dependent on immigrants from the mainland *per se*, but rather can be attributed to a failure to consider immigration overall. As shown by figures S4 & S6, unknown-origin immigrants are typically in the minority.

24. l 179 - As said above, I think this has to be true due to how the animal model works.

This is incorrect. The *mean* breeding value of the base population is fixed to zero, but that does not eliminate the possibility of structure within the population of breeding values, such as a trend in breeding values of founders across time. Nevertheless, our extended analyses permitted immigrants to the Vlieland system (i.e., presumed mainland-origin recruits) to be assigned period-specific distributions of breeding values, distinct also from that of the "native" base population (Fig. S8; described lines 539-572). This had negligible influence on our results (Fig. S11), which we hope ameliorates the reviewer's concern.

Discussion

25. l 189 'In an attempt to resolve the paradox of stasis [7,9], we assess how consistently the breeder's equation— which has proven very reliable for guiding artificially selected evolution of captive [41] and free-living [30,42] populations—fails to predict adaptive trait evolution across a nearly half-centurial study of two, neighbouring, breeding populations.'

I disagree that this study shows that the breeders equation doesn't work. In the period of strong artificial selection, the is strong concordance between predicted and observed response, but in other years, where there is no demonstrable selection, it doesn't do well at picking up response. Personally, I don't think this sheds light on the utility of the breeders equation in the wild.

We have revised the manuscript with a view to addressing these criticisms and no longer apply the breeder's equation, which we agree was flawed.

Yet perhaps a remaining point of contention here is that the reviewer asserts that the breeder's equation is inapplicable when selection is weak (or they might prefer to say 'absent'). However, if selection was null then the breeder's equation does offer a sensible, quantitative prediction of the rate of adaptative evolution: also null. As such, we maintain that quantitative assessment of the paradox of stasis is possible across the full range of selection intensities, including when trait variation appears selectively neutral.

26. l 196 'Thus, if efforts to quantify adaptive responses are made only when traits are a priori known to be under strong selection, then the apparent ubiquity of the paradox of stasis is attributable to observational selection bias'

I'm not sure I get this. So the argument here is that in a trait under no clear directional selection, where immigration dominates, we can find evidence for what resolves the paradox of stasis over/as much as in populations/traits where there clear strong, persistent directional selection but no response?

The argument we were making is that conducting statistical comparison of predicted and inferred responses to selection only when selection is strong is an example of observational selection bias: the test sample is selective because years when selection is weak are deemed unsuitable. The reviewer's implication that the breeder's equation cannot be applied to periods when selection is weak exemplify the preponderance of this fallacy when it comes to this specific issue.

Perhaps it helps to keep in mind that to appropriately quantify the inferred response to selection, we should exclude the immigrants recruited post-selection. In our revised manuscript, these issues are made much more central and discussed more extensively (lines 124-157).

27. l 211 'we show that the genetic impact of immigration is central to understanding contemporary evolution of a key life-history trait, being at least as impactful as natural selection'

In this example! Whilst this might well be true in other cases, I think care should be taken with generalization

Our manuscript does not purport to be a meta-analysis, nor any other form of quantitative review, so we are doubtful that other readers would interpret this sentence in this way. Concise communication will be impossible if "…in this population of great tits on this island over this timeframe" must be appended to every conclusive statement in the manuscript.

28. l 227 'relative isolation does not nullify the evolutionary impact of immigration on traits' evolutionary trajectories'

I would argue that relative isolation was likely to massively amplify the evolutionary impact of immigrants, as the two populations are more likely to be genetically distinct. In a very panmitic situation, I would expect immigration to have much lower impacts, as the genetic differentiation between populations was lower.

Our revised manuscript better clarifies that (*a*) immigrants are identified at the level of the two breeding populations (lines: 70-71, 179-180, 490-492), with Fig. S6 now included to show that migration between the two is the norm; and (*b*) the impact of immigration depends not only on the magnitude of genetic difference between immigrants and natives, but also on their relative frequency (lines 161-163, 282-283), as is now illustrated by Figure 4.

29. l 231 - I completely agree

Methods

Generally I think a lot more information if needed throughout the methods to make this easier to follow.

We hope it's clear that we've recognised the need for considerable need for revision of the manuscript and addressed this. This includes our description of the methods, for which we offer expanded discussion of parts that were causing difficulty for readers.

Study system

30. What proportion of the island population is caught every year? Do the nest boxes in the woodland cover the majority of the population? What I am really getting at here is that are 'unknown' individuals assumed to be all from the mainland, or is there a substantial unringed island population.

There are two distinct issues here. Regarding the first, estimated recapture probabilities for female breeders are 87.6% and 87.7% for West and East, respectively, values that are reported in the revised manuscript (lines 516-517). It is difficult to determine the causes of these observation failures: while one possibility is that they breed in natural cavities, another is that they represent those females that abandoned their breeding attempt before they could be identified.

The second issue concerns the likely source of unknown-origin recruits. As shown in Figure 1, suitable habitat for great tits is limited to the woodlands in which the nestboxes are sited; the rest of the island is scrubland and (~50%) barren sand. As noted in the manuscript (now lines 314-315), the proportion of nestboxes is monitored, with additions made as necessary with a view to maintaining an excess of artificial breeding sites. It is obviously difficult to determine the source of unknown-origin breeders but, as alluded to in the manuscript (lines 265), genetic analysis suggests a mainland origin. (ref. 44).

31. I think there needs to be more discussion of the second clutches. What proportion produce second clutches? How much are these contributing to recruitment (and so fitness as measured here)? Are the recruits in the analysis just from first clutches? Does the likelihood of second clutches differ between populations? Did the selection regimes affect second clutches?

We have added clarification that recruits from any second clutches are included in our measure of annual genetic success (line 401). However, our revised manuscript remains clear that the first observed clutch of each year is our focal trait, so while changes in the frequency of second clutches may be one way by which selection is mediated, it is not pertinent to the questions our study seeks to address, and we feel it will serve to distract the reader when a primary goal during revision has been to improve our manuscript's understandability.

32. l 257 - I agree that it is unlikely, but presumably you could check whether this had carry over effects on the following years reproduction?

Our revised manuscript clarifies that the experiment was restricted to male great tits, and that it did not impact the survival of the experimental individuals, their mates, or their offspring (lines 331-335).

Quantifying Va

33. l 283 - in reference to an comment above - about a third of individuals are founders. It would be good to know how many of the phenotyped 'founder' females had females recruits.

Of 1130 unknown-origin female breeders with an observed clutch size phenotype, 411 have phenotyped daughters.

34. l 288 there is a rogue brackets towards the end of the line.

Thank you. Removed in the revised manuscript (line 373).

35. l 300 Thank you for including some kind of sensitivity analysis

36. l 301 'A thinning interval of 10,000 correlations among successive samples of <0.03 for all random effects' I think there is a missing word somewhere

Yes, this has been corrected in the revised manuscript (lines 386-388).

Quantifying evolutionary change

37. Should the means be weighed by SE or 1/N within each regression? I don't know how much population size varies across years?

All statistical analysis of genetic merits incorporates the statistical uncertainty in individual-level estimates (lines 378-381, 510-512).

Quantifying selection

38. As mentioned in another comment above, this is a recruit-recruit measure of fitness which mixes fitness of parent and offspring. It would be good to acknowledge the potential issues this can cause in terms of estimation of selection (see Thomson and Hadfield 2017 MEE). For example, maternal clutch size might well indirectly affect offspring survival as it affects juvenile size.

This is acknowledged in our revised manuscript (lines 406-412).

39. l 318-328 - This could be much clearer, and I think these results and a much clearer methodology should be presented in the supplements. It is not clear what models were run. If individual linear regressions on relative fitness (relativised by dividing by the mean), then I am not surprised that sometime disruptive selection was found, as there will be clear non-linearity in the fitness function, due to the limit at 0, which will appear like disruptive selection. This could also cloud stabilising selection. Something like a Poisson GLM seems much better suited for this, as it accounts for this inherent non-linearity. It would also be very informative to do a random regression analysis here, looking for the overall direction of selection in each population (possibly per period) and whether it differs between populations (as an interaction), and whether there is detectable variation in selection across years. There is an implicit assumption in the breeders equation analysis that directional selection varies across years, but this never seems to be tested. This cannot be assessed from Figure 2, as there is no uncertainty plotted, and if they are estimates from individual models, then the variance among them will be inflated.

As clarified in our revised manuscript, we have phenotypic and fitness observations for all individuals in our focal populations (lines 403-404, also 437-438). As such, the variation illustrated in Figure 2b demonstrates the across-year variation in directional selection. The legend of Figure 2 also now clarifies this (lines 756-757).

Predicting and inferring local adaptation and Figure 3

40. There is unfortunately not enough information presented here to assess the methodology. It is not clear a) whether uncertainty in selection was quantified and incorporated into the uncertainty on the annual predictions of evolutionary change b) whether the uncertainty in both the annual predictions and the inferred responses was incorporated into the regression (e.g. in a bivariate meta-analysis), c) how the analysis in in Figure 3b was done, as it is not even mentioned in the methods - was this a single model, testing the interaction with the uncertainty incorporated? If not, my concern is that this result is due to sampling variance.

Our revised manuscript explains why uncertainty in selection is not quantified (lines 403-404, also 437-438).

41. It is worth noting that the sensitivity analysis with varying h2 per year doesn't allow the Va to change per year - that is fixed in the animal model - it is essentially allowing the environmental component to vary. So whilst this is a useful additional, it might also be that there is GxE, which this doesn't test.

This is a misunderstanding. By regressing the genetic merits of only those females breeding in a given year on their observed clutch sizes in that year, both phenotypic and heritable variation in clutch size are able to change, as explained in the manuscript (lines 445-449).

42. Very minor point, but the 'CS' subscript on the S seems unnecessary sine there is only selection on clutch size. When I first read it I thought it was an index.

We have removed this subscript from our revised manuscript.

Decomposing evolutionary change

43. l 348 ' democratic' - presumably demographic?

Thank you. Corrected (line 471).

44. I find this part hard to follow, and still can't work out whether the logic of l 375-381 works, or whether the resulting sum of components will give back the correct change. The contribution to a current years mean can also be thought of as the weighted averages of the different groups. It would seem that this much much easier to calculate and explain:

$\bar{a} = p_{survivors}\bar{a}_{survivors} + p_{recruits}\bar{a}_{recruits} + p_{immigrants}\bar{a}_{immigrants}$

where p is the proportion. The contribution of group x can then just be $p_x\bar{a}_x$. Perhaps there is a reason why this wouldn't work and the other method would, but as it stands I don't understand the current method!

The logic of our analysis works, as do the mathematics in practice (we wrote verification into our code to ensure that they summed; see also, e.g., Rebke 2012, *J. Ornithology*). We have expanded the methodological description to aid readers' understanding (lines 470-512), including the addition of a worked example (Fig. 4) to help explain the approach. We have also more strongly highlighted applications of this method elsewhere, including phenotypic change across adulthood (Rebke et al 2010, *PNAS*), community turnover (Fox & Kerr 2012 *Oikos*) and even changes in accounting methods (Waymire & Basu 2011 *Accounting & Business Research*), as well as an algebraic derivation that clarifies this as an extension of the Price equation (Kerr & Godfrey-Smith 2009 *Evolution*) (lines: 470-474).

45. It would be interesting to also separate to within island from mainland immigration.

Yes, it would. However, partitioning each year's incoming and outgoing groups adds additional uncertainty over their quantitative effects unless you are confident of the order in which individuals arrived/departed, as is now explained in the revised manuscript (lines 494-509). This is why we apply the averaging and is also why we limit our decomposition of the recruitment effect to a first-order decomposition of recruits into (patch-level) natives versus immigrants.

46. l 369-373 The relevance of this information for this section is not clear to me.

It relates to answering the comment above (#45). We have revised this section to make this relevance more obvious (lines 494-502).

47. Figure 5 is never described in the methods, and only referred to in passing in the results.

We have added these methodological details (lines 532-537) and here emphasise that it remains cited twice in the Results section (lines 247, 251). It is the basis of our conclusion that the effect of gene flow is unlikely to be constant, and so is an important aspect of our study.

Data Availability

48. Data and code doesn't seem to be available for review (at least the provided DOI didn't work for me). I think this is a shame, as it would have helped me work out what was done in several places where the methods were unclear (especially in the selection analyses), which would save a bit of back and forth in review.

These materials were provided, and other reviewers seemed to have successfully accessed them. We have uploaded the revised code (including saved models) and the primary data to Zenodo, to allow our result to be exactly reproduced. The editorial team have confirmed that they are able to access this repository using this link. https://zenodo.org/records/19203901

Since they have named themselves, we assume that the Reviewer can contact either the editor or ourselves if they remain unable to access these materials and we will be happy to share them directly.

Reviewer #3:

The manuscript "Paradox lost? Gene flow determines trait evolution in a wild bird population" is a timely work that goes beyond the typical approach in a free-living population to explaining evolutionary change based solely on response to natural selection. This manuscript attempts to add gene flow to the evolutionary processes responsible for genetic trends through time by capitalizing on an impressive experimental manipulation conducted within a very long-term dataset on free-living birds.

While incorporating evolutionary change due to gene flow into prediction of phenotpyic evolution from wild populations is an area of great interest currently, there are several aspects to the analysis, approach, and interpretation that need clarification and justification. I elaborate on these main comments and then include some smaller, more specific comments at the end.

Thank you. As we hope is clear, we have extensively revised our analyses and manuscript to offer clearer justification for our study, more detailed explanation of its analyses, a more focussed narrative and a stronger focus on key results and their interpretation.

Main Comments

1. Evolution, demography, and selection

1.1. The authors did not justify the simultaneous use of a framework for prediction from evolutionary quantitative genetics (QG) and several metrics that are not consistent with that framework. Specifically, evolutionary QG theory (e.g., breeder's equation) separates natural selection from inheritance to make predictions about evolutionary change, but the measures of selection and descriptions of demographic changes in genetic value include some individuals across timepoints. This fact that an individual survives and so resembles itself between time periods, conflates selection and inheritance in a way that is not consistent with the QG theory for how inheritance is modeled.

1.1.2. A specific example of point 1.1 comes from contrasting the breeder's equation prediction about change over a generation with the timeline over which the manuscript evaluates changes - namely, each year (in an animal with overlapping generations). The heritability used in the breeder's equation comes from the additive genetic and phenotypic variances estimated in the animal model (Lines 331-344). However, selection is estimated using an individual's own survival as part of its fitness measure. Without year-specific breeding values then the breeder's equation prediction has an inter-generation measure of heritability combined with a measure of selection that uses both intra- and inter-generational change. Consequently, the observed response to selection includes the genetic value of many individuals that occur in both time points for the response to selection (e.g., timepoint x and x+1). This is then not consistent with the heritability measure. Specifically, the breeder's equation will fail to correctly predict the change in genetic merit when the current and future timepoints include a large overlap of individuals.

Thank you for this criticism. As mentioned already, we abandoned reliance on the breeder's equation and now assess how well the relationship between the interannual genetic change corresponds with the within-year heritability, as expected if the observed selection quantifies the causal relationship (see, e.g., Postma 2006).

2. Breeding value prediction
The manuscript relies heavily on the predicted breeding values from the animal model to demonstrate genetic trends through time and evaluate proposed dynamics for how natural selection and immigration will cause these trends in genetic values. Therefore, un-biased breeding value predictions are critical. There are a couple of key points that need to be considered.

2.1. Estimation of mean breeding value for groups (e.g., timepoints) especially need to control for environmental differences in mean phenotype between groups. The authors include woodlot identity as fixed effects in the model, but this does not control for environmental differences across years that affect the mean phenotype (and hence mean breeding value). Environmental differences (Hadfield et al. 2010) or trends (Sorensen et al. 2001) have a great impact on inferences using breeding value predictions derived from quantitative genetic mixed models. Temporal changes in the environment should be removed in order to obtain an environment-independent prediction of breeding values. The solution, then, is to include year-by-woodlot fixed effects in the model.

Sorensen, Fernando, & Gianola. 2001. Inferring the trajectory of genetic variance in the course of artificial selection. Genet. Res. Camb. 77:83-94. DOI: 10.1017/S0016672300004845

Hadfield, Wilson, Garant, Sheldon, & Kruuk. The misuse of BLUP in ecology and evolution. The American Naturalist 175(1):116-125. DOI: 10.1086/648604

Thank you for highlighting these shortcomings in our model. We have addressed these by including fixed effects that model environmental differences for each genetic comparison that we conduct, accomplished by fitting period-and-woodlot-specific temporal trends, as noted in the Methods (lines 366-370).

2.2. Additive genetic variance will be biased when immigrant groups are not specifically recognized in the analysis yet differ on average from the residents (Wolak & Reid 2017). Biased additive genetic variance estimates will cause problems for breeding value prediction since such predictions assume the additive genetic variance is known (see Sorensen et al. 2001 referenced above). This is potentially a problem with the current analyses, given the authors argue that later in the experiment immigrants carry alleles that shift the mean breeding values and there is a substantial proportion of individuals for whom the natal origin is unknown (e.g., Figure S2). Unless specifically treated otherwise, individuals of unknown origin and unknown parentage will be added to the pedigree "founder population". Additionally, there is the further risk that breeding value prediction will be heavily influenced by phenotype particularly for individuals with unknown parents that do not have many offspring with phenotypes. Consequently, an increasing proportion of individuals with unknown origin (e.g., Figure S2) could mean that the trend in breeding values is driven by phenotypic change which is not genetic change. This would support the importance of gene flow, but at the phenotypic and not genetic level. The phenotypic and genetic levels will diverge particularly if there are environmental trends through time (which the authors note).

Wolak, M.E., & J.M. Reid. 2017. Accounting for genetic differences among unknown parents in microevolutionary studies: how to include genetic groups in quantitative genetic animal models. J. Anim. Ecol. 86:7-20. doi: 10.1111/1365-2656.12597

As described in detail in the Materials and Methods (lines 539-572), we applied the genetic groups approach, initially recognising five distinct source populations (Fig. S8) and then consolidating these to two (Fig. S9a). As discussed in our revised manuscript, this approach did not offer the advantages expected by reviewers and came at a cost of introducing huge uncertainty in the absolute values of individual genetic merits (and derived statistics) (Fig. S11). This is explicable based on the ancestral composition of the population (Fig. S10). Thus, while these additional analyses add value to our study, we feel the main results should remain based on a genetic group naïve animal model.

3. No tests for the effect of drift
Though this is recognized (e.g., Line 133), it is surprising that some form of the Hadfield et al. 2010 method of simulating expected changes in breeding values due to drift was not implemented. Without this, the changes in mean genetic merit cannot be attributed to either natural selection or immigration without drift being a plausible null hypothesis.

Our revised manuscript explicitly states that the demographic decomposition does not (and cannot) attribute genetic change to natural selection, since *in situ* genetic change will also be subject to drift (lines 182-183). This same sentence also clarifies that the decomposition approach quantifies the contribution of *demographic* processes to genetic change. As such, we disagree with the reviewer that (*a*) our method is unable to quantify the contribution of immigration. We have, though, accommodated Reviewer 3's desire to see the likelihood of drift alone accounting for genetic change, presented in Table S3.

Detailed Comments

4. Lines 88 to 93: The authors do not consider uncertainty in selection estimates when aggregating over them to test the significance or difference between groups.

Our revised Methods explains for the reader that sampling uncertainty is not applicable to our selection estimates (lines 403-404, 437-438), as is also noted in the legend for Figure 2.

5. Lines 95 to 98: The authors describe the conclusions about the system by contrasting a situation of little to no adaptive evolution despite strong selection to the observation from their results of genetic change/evolution in the absence of strong natural selection. It is unclear whether the statement about no adaptive evolution despite strong selection refers to the general paradox of stasis or whether they are invoking this to summarize the pre-experimental period. Either way, such conclusions seem at odds with the results that there are no differences in the strength of selection when comparing the pre- and post-experiment periods (My comment 4. Lines 88 to 93) - though see my other comment about this result.

As part of our broader revision of the manuscript, we have sought to simplify application of the paradox of stasis.

6. Line 266: Please expand to clarify this sentence.

We have revised this section, now providing the reader with more details (lines 342-351).

7. Line 288: There is a stray ")" after "effect".

Thank you. Corrected (line 373).

8. Lines 297 to 299: I do not think the description of this prior is correct. For example, as the prior was coded with `alpha.nu = diag(1) * 1000` that does not refer to the prior variance but a hyperparameter on the variance. The prior was specified `G1 = list(V = diag(1), nu = 1, alpha.mu = 0, alpha.V = diag(1) * 1000)` so it would be more informative and perhaps more correct to say this prior is a "... parameter expanded prior that is relatively uninformative as it closely approximates the scaled non-central F-distribution with numerator and denominator degrees of freedom equal to one and a scale parameter of 1,000 (Gelman 2006; Hadfield 2010)."

Gelman A. 2006 Prior distributions for variance parameters in hierarchical models
(comment on article by Browne and Draper). Bayesian Anal. 1, 515-534.
(doi:10.1214/06-BA117A)

Hadfield J. 2010 MCMC methods for multi-response generalized linear mixed models:
the MCMCglmm R package. J. Stat. Softw. 33, 1-22.
(doi:10.18637/jss.v033.i02)

Thank you. We have revised this sentence following the Reviewer's suggestion (slightly reworded to accommodate applicable advice that was given to us by a third party) (lines 382-384).

9. Lines 301 to 302: This sentence seems to be missing part of the middle between "10,000" and "correlations". Also, are the auto-correlation values for the random effects of the variance of random effects?

Yes, words were missing, and we were referring to autocorrelation values of the random effects. The revised sentence provides these details (line 386-388).

10. Line 348: "democractic" should be "demographic"

Thank you. Corrected (line 471).
