# Supplementary material for: Gene flow drives periods of both evolutionary stasis and change in a wild bird population
Source: PLoS Biol. 2026 Sep 21;24(9):e3004004. doi: 10.1371/journal.pbio.3004004 (PMC13630318; doi:10.1371/journal.pbio.3004004)
Supplement: Supplementary file 14 [file pbio.3004004.s012.docx]

| Variance source | Posterior mean [95% HDPI] (eggs^2^) | (*a*) As proportion of $V_{P}$ [95% HDPI] | (*b*) As proportion of $V_{P_{within-year}}$ [95% HDPI] |
| --- | --- | --- | --- |
| Year | 0.180 [0.111, 0.263] | 0.091 [0.059, 0.129] | – |
| Additive genetic | 0.409 [0.296, 0.509] | 0.206 [0.157, 0.259] | 0.226 [0.172, 0.284] |
| Permanent environment | 0.420 [0.320, 0.533] | 0.212 [0.158, 0.264] | 0.233 [0.177, 0.292] |
| Maternal | 0.036 [0.000, 0.095] | 0.018 [0.000, 0.048] | 0.020 [0.000, 0.052] |
| Residual | 0.941 [0.896, 0.989] | 0.474 [0.443, 0.504] | 0.521 [0.493, 0.550] |
