# Supplementary material for: Gene flow drives periods of both evolutionary stasis and change in a wild bird population
Source: PLoS Biol. 2026 Sep 21;24(9):e3004004. doi: 10.1371/journal.pbio.3004004 (PMC13630318; doi:10.1371/journal.pbio.3004004)
Supplement: Supplementary file 15 [file pbio.3004004.s013.docx]

| **Period** | **Timespan** | $\boldsymbol{S}$ | | $\boldsymbol{\beta}_{\boldsymbol{\mu}}$ | |
| --- | --- | --- | --- | --- | --- |
|  |  | **West** | **East** | **West** | **East** |
| *Pre-experimental* | 1980-1995 | 0.00 | 0.04 | -0.05 | 0.11 |
| *Experimental* | 1996-2003 | -0.22 | 0.41 | -1.12 | 2.12 |
| *Post-experimental* | 2004-2011 | -0.05 | 0.05 | -0.34 | 0.21 |
| *Legacy* | 2012-2022 | -0.01 | 0.11 | 0.11 | 0.43 |
