# Supplementary material for: Gene flow drives periods of both evolutionary stasis and change in a wild bird population
Source: PLoS Biol. 2026 Sep 21;24(9):e3004004. doi: 10.1371/journal.pbio.3004004 (PMC13630318; doi:10.1371/journal.pbio.3004004)
Supplement: Supplementary file 16 [file pbio.3004004.s014.docx]

| **Period** | **Timespan** | **West** | | **East** | | **West-East contrast** | |
| --- | --- | --- | --- | --- | --- | --- | --- |
|  |  | **Rate** **(eggs/yr)** | ***P*(rate\|drift only)** | **Rate** **(eggs/yr)** | ***P*(rate\|drift only)** | **Rate** **(eggs/yr)** | ***P*(rate\|drift only)** |
| *Pre-experimental* | 1981-1996 | 0.005 [-0.010, 0.020] | 0.472 | -0.002 [-0.016, 0.010] | 0.622 | 0.007 [-0.008, 0.024] | 0.479 |
| *Experimental* | 1997-2004 | -0.005 [-0.031, 0.022] | 0.543 | 0.040 [0.019, 0.064] | 0.022 | -0.045 [-0.078, -0.012] | 0.107 |
| *Post-experimental* | 2005-2012 | 0.024 [-0.001, 0.051] | 0.212 | -0.014 [-0.031, 0.001] | 0.260 | 0.038 [0.010, 0.069] | 0.097 |
| *Legacy* | 2013-2023 | -0.003 [-0.021, 0.017] | 0.561 | 0.002 [-0.011, 0.016] | 0.543 | -0.005 [-0.026, 0.015] | 0.550 |
