# Supplementary material for: Gene flow drives periods of both evolutionary stasis and change in a wild bird population
Source: PLoS Biol. 2026 Sep 21;24(9):e3004004. doi: 10.1371/journal.pbio.3004004 (PMC13630318; doi:10.1371/journal.pbio.3004004)
Supplement: Supplementary file 17 [file pbio.3004004.s015.docx]

| Variance source | Posterior mean [95% HDPI] (eggs^2^) | (*a*) As proportion of $V_{P}$ [95% HDPI] | (*b*) As proportion of $V_{P_{within-year}}$ [95% HDPI] |
| --- | --- | --- | --- |
| Year | 0.432 [0.276, 0.598] | 0.190 [0.132, 0.248] | – |
| Additive genetic | 0.440 [0.329, 0.554] | 0.195 [0.148, 0.243] | 0.240 [0.186, 0.298] |
| Permanent environment | 0.411 [0.309, 0.518] | 0.182 [0.132, 0.228] | 0.225 [0.170, 0.283] |
| Maternal | 0.030 [0.000, 0.088] | 0.013 [0.000, 0.039] | 0.016 [0.000, 0.049] |
| Residual | 0.947 [0.901, 0.996] | 0.420 [0.383, 0.456] | 0.518 [0.489, 0.546] |
