# Supplementary material for: Gene flow drives periods of both evolutionary stasis and change in a wild bird population
Source: PLoS Biol. 2026 Sep 21;24(9):e3004004. doi: 10.1371/journal.pbio.3004004 (PMC13630318; doi:10.1371/journal.pbio.3004004)
Supplement: Supplementary file 18 [file pbio.3004004.s016.docx]

|  | Variance source | Posterior mean [95% HDPI] (eggs^2^) | (*a*) As proportion of $V_{P}$ [95% HDPI] | (*b*) As proportion of $V_{P_{within-year}}$ [95% HDPI] |
| --- | --- | --- | --- | --- |
| *Constant = 0.1* | | | | |
|  | Year | 0.184 [0.111, 0.264] | 0.092 [0.057, 0.127] | – |
|  | Additive genetic | 0.397 [0.290, 0.504] | 0.200 [0.147, 0.250] | 0.220 [0.166, 0.278] |
|  | Permanent environment | 0.425 [0.320, 0.529] | 0.214 [0.158, 0.266] | 0.236 [0.177, 0.294] |
|  | Maternal | 0.039 [0.000, 0.098] | 0.019 [0.000, 0.050] | 0.021 [0.000, 0.055] |
|  | Residual | 0.941 [0.896, 0.990] | 0.474 [0.444, 0.508] | 0.522 [0.494, 0.551] |
|  |  |  |  |  |
| *Constant = 1* | | | | |
|  | Year | 0.184 [0.111, 0.262] | 0.092 [0.060, 0.129] | – |
|  | Additive genetic | 0.399 [0.298, 0.511] | 0.201 [0.153, 0.256] | 0.221 [0.168, 0.281] |
|  | Permanent environment | 0.426 [0.319, 0.538] | 0.215 [0.162, 0.272] | 0.236 [0.179, 0.298] |
|  | Maternal | 0.037 [0.000, 0.096] | 0.018 [0.000, 0.048] | 0.020 [0.000, 0.053] |
|  | Residual | 0.940 [0.895, 0.988] | 0.474 [0.442, 0.505] | 0.522 [0.497, 0.553] |
|  |  |  |  |  |
| *Constant = 10* | | | | |
|  | Year | 0.181 [0.115, 0.261] | 0.091 [0.060, 0.127] | – |
|  | Additive genetic | 0.406 [0.299, 0.511] | 0.204 [0.153, 0.257] | 0.225 [0.168, 0.278] |
|  | Permanent environment | 0.421 [0.319, 0.530] | 0.212 [0.163, 0.271] | 0.234 [0.178, 0.295] |
|  | Maternal | 0.036 [0.000, 0.097] | 0.018 [0.000, 0.049] | 0.020 [0.000, 0.054] |
|  | Residual | 0.941 [0.894, 0.988] | 0.474 [0.443, 0.505] | 0.522 [0.493, 0.550] |
