# Supplementary material for: Gene flow drives periods of both evolutionary stasis and change in a wild bird population
Source: PLoS Biol. 2026 Sep 21;24(9):e3004004. doi: 10.1371/journal.pbio.3004004 (PMC13630318; doi:10.1371/journal.pbio.3004004)
Supplement: Supplementary file 19 [file pbio.3004004.s017.docx]

| Variance source | Posterior mean [95% HDPI] (eggs^2^) | (*a*) As proportion of $V_{P}$ [95% HDPI] | (*b*) As proportion of $V_{P_{within-year}}$ [95% HDPI] |
| --- | --- | --- | --- |
| Year | 0.184 [0.111, 0.264] | 0.092 [0.057, 0.127] | – |
| Additive genetic | 0.397 [0.290, 0.504] | 0.200 [0.147, 0.250] | 0.220 [0.166, 0.278] |
| Permanent environment | 0.425 [0.320, 0.529] | 0.214 [0.158, 0.266] | 0.236 [0.177, 0.294] |
| Maternal | 0.039 [0.000, 0.098] | 0.019 [0.000, 0.050] | 0.021 [0.000, 0.055] |
| Residual | 0.941 [0.896, 0.990] | 0.474 [0.444, 0.508] | 0.522 [0.494, 0.551] |
