# Supplementary material for: Gene flow drives periods of both evolutionary stasis and change in a wild bird population
Source: PLoS Biol. 2026 Sep 21;24(9):e3004004. doi: 10.1371/journal.pbio.3004004 (PMC13630318; doi:10.1371/journal.pbio.3004004)
Supplement: Supplementary file 20 [file pbio.3004004.s018.docx]

| Variance source | Posterior mean [95% HDPI] (eggs^2^) | (*a*) As proportion of $V_{P}$ [95% HDPI] | (*b*) As proportion of $V_{P_{within-year}}$ [95% HDPI] |
| --- | --- | --- | --- |
| Year | 0.189 [0.119, 0.272] | 0.094 [0.061, 0.131] | – |
| Additive genetic | 0.405 [0.297, 0.509] | 0.203 [0.152, 0.252] | 0.224 [0.168, 0.279] |
| Permanent environment | 0.421 [0.316, 0.524] | 0.211 [0.158, 0.263] | 0.233 [0.173, 0.287] |
| Maternal | 0.039 [0.000, 0.099] | 0.019 [0.000, 0.050] | 0.021 [0.000, 0.055] |
| Residual | 0.941 [0.893, 0.989] | 0.472 [0.441, 0.503] | 0.521 [0.492, 0.549] |
