# Supplementary material for: Gene flow drives periods of both evolutionary stasis and change in a wild bird population
Source: PLoS Biol. 2026 Sep 21;24(9):e3004004. doi: 10.1371/journal.pbio.3004004 (PMC13630318; doi:10.1371/journal.pbio.3004004)
Supplement: Supplementary file 21 [file pbio.3004004.s023.docx]

Reviewer comments are formatted using black Helvetica and our replies are in dark blue Cambria. Line numbering refers to the revised version without changes marked.

# Reviewer #1:

I want to commend the authors on a much improved manuscript.  The focus and writing are now much clearer to my eyes and the manuscript is now much more effective at addressing the more limited set of goals.  As currently framed, the manuscript now focuses specifically on how immigration can affect evolutionary changes in addition to in situ changes in that trait in response to natural selection.  As the authors correctly mention in their manuscript, most long-term studies of individually marked animals that are used to perform studies such as this are 'by design' relatively isolated.  This means that immigration is minimized with respect to other evolutionary processes, but this does not mean that it is completely eliminated. This study does a very nice job of showing the importance of this immigration to evolutionary change and the degree to which this might cloud our ability to detect evolutionary responses to natural selection within the population (i.e. apparent paradox of stasis).

Thank you.

There is one more technical issue that I wanted to discuss and there are some ways in which I think the authors could make their case more strongly.  First, I think we all agree that by definition the mean breeding value of base animals in the pedigree is defined to be zero.  Base animals are those at the start of the study and immigrants with unknown parents.  I was, therefore, confused by the sentence on lines 247-249.  I am unclear on how the genetic merit of unknown-origin immigrants could change since their mean breeding values should always be zero. This made me worry I was misunderstanding something here.

The reviewer is correct that the breeding values of the base population (i.e., all individuals with unknown parents) are assumed to come from a normal distribution with a *mean* of zero and a variance that defines the additive genetic variance. However, their individual breeding values are predicted from their own clutch size and the clutch size of their female descendants. In other words, while the breeding value of all immigrants combined are expected to have a mean of zero (ignoring the small contribution of non-immigrants that are also part of the base population; see below), the model does not constrain the mean of subsets of immigrants (for example those immigrating in different years) to be identical.

Although different parts of the base population may thus have different mean breeding values, the model assumes that the distribution of all breeding values of the base population is described by a single unimodal distribution. Genetic group models allow relaxing this assumption by explicitly modelling the difference in the mean breeding value among different groups of individuals making up the base population. In response to previous reviewers’ comments, we have explored this in some detail but concluded that in this case, genetic groups do not provide any inferential benefits. Primarily this is because in this system, the very great majority of the base population is made up of immigrants. Therefore, the difference in mean breeding value between immigrant and individuals assumed to be present on Vlieland in 1955 is estimated with great uncertainty, and this uncertainty is then propagated to the breeding value predictions of non-base population individuals. Importantly, as we show in the supplementary materials, modelling genetic groups does not change any of the patterns in the mean breeding values.

Finally, in addition to the possibility of the absolute mean breeding value of immigrants changing over time, we show how the impact of immigrants depends on their mean breeding value relative to that of the locally-born population. Hence, if the latter changes, the relative mean breeding value of immigrants, and their population-level impact, will change too.

On the whole, this shows immigration to be a highly dynamic evolutionary driver that can create directional evolutionary change that looks like adaptation, as well as mask adaptive evolution. Indeed, we consider this is one of the main messages of our manuscript and we have therefore made various minor edits to the manuscript to better highlight this.

Beyond this specific point, I think there is a more general point that the authors could make more strongly.  What I think is unique and very valuable about this study is that much of the immigration was not by animals of unknown origin but instead was of animals of known origin from another population.  I think this is very different from most comparable studies in which almost all immigration will be by animals of unknown origin.  This the case of other studies this animals are assumed to have a mean breeding value of zero and they will simply dilute any population change in mean breeding value.  In contrast, the breeding values of immigrants in this study can be substantially non-zero because they are often not defined to be base animals.  This leads to the very interesting plot (Fig 5) showing how very non-zero breeding values in immigrants could accentuate or oppose in situ evolution. I know this is the authors' main point but I think they could make their case stronger for the way in which their study has revealed a process that is otherwise very difficult to uncover.  Mean breeding values of unknown immigrants is often very likely different from zero but when we have no other information we must assume the mean is zero.  This study shows how we might be quite misled in this necessary assumption. I would suggest that the authors try and make this case more strongly since I think this is one of the most impactful stories of this study.

See above for a discussion of why unknown-origin immigrants do not necessarily have a mean breeding value of zero. We agree that the impact of gene flow between both subpopulations is potentially different from immigration from outside Vlieland, but here we primarily focus on the impact of immigration from the perspective of the recipient population. In the interest of simplicity, we pool both sources of immigrants when quantifying the impact of immigration. Distinguishing between the two would add further complexity to analyses and interpretation alike (as discussed in our previous Responses to Reviewers document), and distract from our main message.

My other general comment is that I don't think the authors have fully discussed the degree to which immigration explains the observed mismatch between expected and observed responses to selection.  To be transparent the degree of uncertainty around the metric for the degree to which observed change is below expected change is so large that the change observed with immigrants is not statistically different from the in situ change.  I will be honest and say that i am not too hung up on these p-values and uncertainty around these metrics.  I appreciate the authors' quantitative approach. Others might be more concerned about the P-values so I would suggest the authors be more transparent about how this high uncertainty might be interpreted.  Quantitatively though observed changes were only 37% of expected (if I understand the metric correctly), whereas after accounting for immigration the observed in situ changes were 75% of expected.  Quantitatively this story is interesting to
me despite the large uncertainty in these metrics.  Immigration accounted for a large component of the 'stasis', but I think it is worth also discussing how even after accounting for immigration there remained some degree of stasis.  I think this needs to be discussed at the end of the paper.  Some stasis can be explained by immigration in this system but not all stasis.

Thank you for highlighting this. We have slightly revised the title to better reflect our inability to robustly conclude that we resolved the paradox of stasis (line 1). We fully agree with the reviewer that the uncertainty around our inferred and predicted evolutionary change are very large. Indeed, one of our messages is that this uncertainty needs to be considered when invoking the paradox of stasis: it requires a comparison of inferred and expected change, and not of the inferred changed against zero. The latter is the norm, and we posit that in most cases of supposed stasis, the inferred genetic change is not meaningfully different from the expectation (in addition to not being meaningfully different from zero).

Although we have considered removing this analysis from the manuscript, we believe that it allows us to make several important points, including the large uncertainty around our estimate of the difference between inferred and expected evolutionary change also highlighted by the reviewer. Although we agree our results suggest that excluding immigrants improves the predictability of evolution (the inferred change is closer to our expectation), we are reluctant to make any firm statements about whether or not the inferred change is equal to the expectation. Instead, we prefer to leave this up to the reader, who we provide with all estimates and their uncertainty. Having said this, we agree with Reviewer 3 that there still was some room for improvement when it comes to how this section is embedded with the manuscript. See our responses to this below.

Overall, I think there are some important new lessons to be learned from this study.  The authors have a unique study system that has allowed them to use clever and innovative approaches to reveal some interesting patterns that could not have been studied in other systems.  These patterns have the potential to explain some (but not all) of the more general pattern of observed evolutionary changes being weaker than expected based on quantitative genetic predictions.  Together, I think this makes for a compelling and valuable study.

Thank you.

Some Specific Comments:

- text around figures 2 and 3 suggests that there is no sampling error around the estimate of S.  I suppose this is true in the calculation but there is still uncertainty in this measure that can be calculated through resampling techniques. That said, I'm not connived that ignoring uncertainty in S is detrimental to the overall story.

We discuss at lines 426-428, as well as in our previous response to reviewer comments, why we chose not to model uncertainty in S. In short, they are based on sampling the complete breeding population, and we acknowledge that they capture both selection and drift.

- please be clear in the figure legends about exactly what years were analyzed.  I think the authors excluded the experimental period (based on the response letter) but this wasn't clear to me in the text.

This information is included in the legend of Figure 2 (lines 91-94); is it possible that the reviewer overlooked this? For all other main figures, the timespans are provided as annotations of the plots.

- Fig 3 essentially depicts a realized heritability, but I don't think it is described in this way in the legend or the text.

Thank you. We now note in the legend of Figure 3 that the slope of this regression can be interpreted to be (half) the realised heritability (lines 146-147), and we discuss some of the intricacies surrounding the observed versus the expected slope of this line in the Methods.

-  Line 157.  The Paradox of stasis was reduced (not significantly because the uncertainty around the 0.367 and the 0.749 are very large) but not eliminated.  This needs to be more fully discussed.  I don't think the degree to which immigration can explain stasis in this system is currently discussed.

As highlighted by the reviewer and discussed above, inferred and expected slopes (and their ratios) are estimated with large amounts of uncertainty. Our revised manuscript includes the posterior probability that the underestimation of evolutionary change is lessened, and we explicitly caution the reader that strong statistical support is lacking (lines 158-160) This uncertainty is also reflected in our revised title, into which we have introduced a question mark (line 1).

We make various statements regarding the quantitative impact of immigration, including in the abstract, and we have made some minor edits to add further clarity. We hope these address the reviewer’s request.

- Side note regarding above - I think the magnitude of the confidence intervals is so large that it is not informative to measure these ratios (delta a/bo) to three decimal places.  Perhaps only one is sufficient.  Evolutionary change goes from 0.4 of expected to 0.7 of expected.

We agree and have revised the precision to two decimal places, which we hope is an acceptable compromise (lines 131, 137, 138, 158, 159).

# Reviewer #2:

This revision of the paper has addressed many of my previous comments. Overall I really like the study. I think it has a lot of value, and many of the analyses are very insightful. I think the results are well suited to PLOS Biology.

We are glad for the positive overall view of our study. Below we respond in some detail to the further comments and suggestions, and outline how we have incorporated some of Reviewer 2’s suggestions, but also justify why we chose not to incorporate others.

As authors we are advised against selectively responding to reviewers’ comments. Because Reviewer 2 reiterates many of his previous comments but has chosen to ignore our responses, we repeat some of our previous interaction below.

However, I found the manuscript quite hardwork to follow in places. I would suggest that an easier way for a reader would be to clearly set out at the end of the introduction what hypotheses are being tested/ what data is being used to test them, and then include subheading in the results to help guide a reader through, preferably matched to the methods. The intro focuses on immigration, but then the results goes through a performance of setting up the return after selection ends should be due to natural selection, and poses a new paradox. However, as a reader this seems unnecessary, as immigration has already been introduced as a solution. The results also move back and forth between the selection experiment and overall selection/response to selection in the population as a whole. I find this a bit difficult to deal with as a reader. Again feel that a bit of reorganisation, better signposting in the introduction of what is to come, and some subheadings would really help.

We acknowledge that we are telling a complex story, but we believe that, with the help of two rounds of review by three reviewers, we have arrived at a version with a clear narrative that strikes the right balance between clarity and conciseness. Since the other reviewers were positive about our revised manuscript, we have made various minor edits but have not pursued further restructuring.

Main comment

I still am struggling to put this study into the context of the paradox of stasis/general problem of misprediction.
The paradox of stasis, in my opinion (and I think is supported by commonly cited papers in this area e.g. Merila et al 2001, Pujol et al 2018), is typically used to refer to traits under strong persistent direction selection, i.e. the same selection pressure (give or take) over time, and across populations, and often species (an example of this being juvenile body size). In the response, it is stated that the paradox of stasis is more about unmet expectations - whilst I agree that this is a more general problem, into which the paradox of stasis fits, I think the paradox of stasis itself is more specific than this - after all it is a paradox of *stasis*, i.e. why we find stasis when we don't expect to, due to strong persistent directional selection. This is a matter of framing, and ultimately up to the authors, but I suspect other readers may have a similar response.

This argument is effectively repeated two paragraphs later, where we offer a response.

However, my issue is a broader point about misprediction (or however we choose to define the paradox of stasis). I think the second paragraph of the introduction gets to the crux of my problem. I can see that for very isolated populations, like the ones cited, there may be very local selection pressures, and the few immigrants from other populations will erode selection response, if those other populations face different selection pressures. In this case, we may study that isolated population, and then wonder why there is no response to the selection. My question is, how common is this? I agree that generally in wild quant gen studies, we do often ignore gene flow. However, for immigration to erode selection response, there needs to be clear differences in the selection pressures in populations connected by immigration. Yet, there seems reasonable evidence that strong selection is often temporally and spatially consistent, especially in direction (Morrissey and Hadfield 2012, Siepielski et al. 2013 ), suggesting pockets of strong and persistently different selection within a metapopulation would be unlikely, especially in traits that are emblematic of the paradox of stasis.

We discuss the generality of our findings in some detail in the *Discussion* section of our manuscript. Ultimately, the question regarding the importance of immigration and gene flow across species and populations is an empirical one. In our manuscript, we argue that to date their impact has not been assessed in a systematic and quantitative manner, which makes this an open question in need of further research. Our demographic balancing equation approach provides a valuable tool that is readily applicable to other systems and should bring us closer to an answer.

In this population, clutch size appears not to be under persistent directional selection, so the paradox of stasis does not refer to the phenotype as a whole in this population. Rather selection appears to fluctuate around 0. Although it is argued that you do not need to consider this selection with uncertainty (discussed more in comments below), the reality is that differences in fitness among individuals, especially in small populations (as here, especially in the west), will largely reflect stochastic variation, and so there will be a certain amount of apparent selection by chance (i.e. drift), fluctuating around zero. There is no apparent test as to whether this is more variation in selection than would be expected by sampling variance alone. This apparent selection and the subsequent genetic change are matched up and used as a test for stasis. However, in the years outside of the selection experiment, it is difficult to see that this is a test of stasis, given that there is little evidence that this is selection rather than drift. In terms of general misprediction, the initial comparison of selection and response includes immigrants. Given the precise definition of selection, this seems misleading to present first, and indeed when the immigrants are removed the prediction matches much better.

We agree with the reviewer that some of the association between clutch size and fitness may be the result of drift. Indeed, our manuscript acknowledges the role of drift in shaping (variation in) the covariance between clutch size and fitness (lines 181-183, and now also 123-127, 425-426) . We emphasise that in the interest of brevity and in line with the common practise, we refer to this covariance as the selection differential (lines 123-124. As suggested by the reviewer, we have included a quantification of the temporal (among non-experimental years) and spatial (between subpopulations) variation in directional selection (lines 125-127). As expected, drift is the primary source of variation in S. We then go on to explain that because we sample the complete breeding population, our expectation for an evolutionary response that is approximately proportional to the heritability remains valid if the association between clutch size and fitness has come about solely by chance (lines 127-129). Indeed, we show that on average and after accounting for immigration, the association between clutch size and fitness is a reasonably good—albeit imprecise—predictor of evolutionary change (lines 150-160).

We also agree with the reviewer that the immigrants should be excluded from estimates of selection, yet this is not common practise. Indeed, with the interesting exception of three studies that quantify the response to *artificial* selection, studies of wild populations’ responses to selection rely on the overall evolutionary change (or do not clarify whether they isolated the *in situ* genetic change, leading us to assume that they did not). We therefore first present an analysis including immigrants, without which we are unable to quantify their evolutionary impact.

The study also focuses on an experiment inducing a short period of intense selection. However, this selection is geographically isolated (indeed being in opposite direction in two neighbouring populations), in populations with high immigration rates. During the period of selection, there seems to be a clear response to that selection, indicating that while selection is acting, immigration does not prevent this response, i.e. immigration does not drive stasis, or misprediction? When it stops, the response is then swamped by immigration, from surrounding areas. If in an artificial selection experiment in the lab, I selected for clutch size, and then stopped selection and bred the lines with an unselected control line afterwards, it would not seem a surprise that they return to the control phenotype.  Again, I find this very hard to reconcile with the paradox of stasis. Before the experiment, there was no persistent directional selection, and there was no change. During the experiment, there was strong directional selection and there was a change. After the experiment, when there was no persistent directional selection, and high immigration from surrounding unselected, or differentially selected areas, the clutch size then returned to the previous state. In each case I think that this is what we would predict?

I don't say this to try and diminish the ambition or importance of this study. I think examining what is happening in a population with this level of detail is extremely illuminating, especially in the context of this large scale quant gen experiment, which is very rare. I think the decomposition of what is driving change in the system, in terms of immigration and in situ change is very elegant, and overall this is a very valuable study. However, the introduction sets it up as pitching for a mechanism that would provide a general solution to the paradox of stasis. Whilst I love a good pun, the title even hints that we have solved the paradox. I don't think that this study can provide a general solution to this paradox, without providing compelling evidence that this phenomenon (very strong, but temporary and localised selection, in a background of no selection) is common, and represents a large amount of what we see when referring to the paradox of stasis.

I would therefore again encourage the authors to consider a slight reframing, and particularly in the context of the 'paradox lost' part of the title. Although I see this as an important and interesting study, I do not think it takes us nearer to a resolution of the paradox of stasis, especially as there is likely no global solution.

Firstly, the Introduction explains that clutch size is a fundamental measure of reproductive rate (lines 56-57), so it is a reasonable supposition, *a priori*, that this character will be subject to selection. Indeed, as we have argued elsewhere (Evans & Postma 2025 *Evolution*), clutch size is directly related to annual reproductive success. As we immediately go on to note (lines 57-58), clutch size has also been the subject of repeated study to resolve a discrepancy between observed selection and apparent response. Given this background, is it insensible to present our study as we do? Although beyond the scope of this manuscript, future work examines why the association between clutch size and proxies for fitness are not stronger. Nevertheless, we have revised the title in response to Reviewer 2’s concerns about the prematurity of its message (line 1).

Secondly, the artificial selection was focussed on clutch size. That this exceptional selection is expected to result in adaptation is the basis of our verification of the demographic process (lines 185-193). As the opening to this section of the results, it is an integral part of manuscript.

Reviewer 2 formulates several predictions with respect to the effect of gene flow on the evolutionary dynamics of clutch size. Our manuscript provides an empirical test of these predictions. The reviewer implies that the fact that he predicted—in part—the trajectory of trait evolution renders investigation unnecessary. Yet it’s worth highlighting that the impact of immigration could be much smaller than expected by the reviewer. For example, there may be selection against immigrants, for which there is evidence in this population (as discussed in detail in the manuscript), or it might be overruled by the effects of drift (which the reviewer highlights elsewhere).

Finally, it is worth nothing that previously Reviewer 2 referred to the study population as unusually isolated, making it of limited general relevance. In this comment, however, he highlights the high immigration rates. We also point out that Reviewer 1 saw the demographics of our study system as a major strength, encouraging us to emphasise the insights they offer.

Specific comments

1. L39 'the 'paradox of stasis' has withstood attempts at general resolution and become emblematic of our struggle to understand contemporary adaptive evolution in natural populations.'
I agree with the second half of this sentence, but do we expect a general resolution? In the well cited concept papers on this topic (e.g. Merila et al. 2001, Pujol et al. 2018), there has been no suggestion of a global solution - indeed they suggest many, not mutually exclusive reasons. Also some work has found resolution of the problem within single populations (e.g. Mittell et al 2025).

We have revised this sentence (line 39).

2. L42 'the paradox of stasis is seldom subject to objective assessment; rather, it is generally evoked a posteriori.'
I don't think I quite get this. I would say that a lot of studies in recent years have focussed on why there is no change, for example, looking at environmental covariances, missing fraction, phenotypic skew, social interactions and immigration. This makes it sounds as though lots of studies are targeted at finding a change, and then when they don't find one, throw out the paradox of stasis as a general solution. This is not my experience of the literature. Perhaps I have missed the point the authors are trying to make, but then it should perhaps be more obvious!

Its imprecision makes it difficult to respond this comment. Whereas the first half is a reasonable summary of the point we are making in the manuscript, the reviewer than invokes his personal experience of the literature to disagree with this point. Nevertheless, we have added more detail to this sentence to better guide the reader (lines 40-43).

3. L47 'Immigrants are likely to be genetically distinct from their host population'

This seems a bold claim, and I think this needs some context, and perhaps some evidence. The two references given are of isolated island populations, with low migration rates, that are more likely to be genetically distinct. In the context of many studies looking at the paradox of stasis, (including many mainland populations), it seems unlikely this is generally the case. It seems important to point this out, when discussing a 'general resolution' to this problem.

We answered this criticism in our responses to the last set of reviews (see Minor Comment 7 of 48), as well as elsewhere in this response and in the manuscript itself.

4. L 64. Once again, it would be helpful to frame this study in the light of the previous work done on this population, in which differential immigration from the mainland was found to be a major driver of the differences between populations.

We have referenced this study where appropriate (lines 62, 84).

5. Figure 2 - A zero line would be helpful for the selection plot (b). I would also suggest adding errors to these estimates! As I discuss in a comment below, I understand the argument that the selection differential used in the regression of selection and response - the in situ selection here is known from the breeders and recruits. However, in small populations, there is considerable drift, driven by stochastic associations between a phenotype and fitness. The uncertainty on these estimates therefore gives an idea of the level of uncertainty there is in whether and how often these values differ from 0, and so how much of this apparent selection might represent neutral processes.

This figure is already very dense, so we have spent considerable time trying to maximise the information to ink ratio. While we acknowledge that annual variation in the selection differential will at least partly be attributable drift (lines 182, 220-222), it is nevertheless expected to predict evolutionary change. We have revised lines 123-127 to make this clearer.

6. L104'This evolutionary return toward the pre-experimental state is consistent with the adaptationist expectation that the experimental evolutionary perturbation (which was driven by strong directional artificial selection) is opposed by natural selection, with local adaptation returning the difference between the two populations toward the status quo ante.'
I am a little confused by this statement. The pre-experiment state was a difference between the two populations. After the selection, the two populations are the same. I would not interpret this as things returning to the status quo, as they are clearly different post selection. If local adaptation was acting before and after, then they should also be different after. As per a comment above, previous studies of this system have also shown that this difference appeared to be quite substantially driven by immigration, so I think this phrasing is a little misleading.

It is important to recognise the word ‘toward’ in this sentence. Doing so would, we suggest, resolve Reviewer 2’s confusion. Nevertheless, we have revised this sentence to read, “This *initial* evolutionary return…” (line 103).

7. L 107 'However, in contrast to this scenario, annual selection on clutch size after the experiment was negligible (one-sample t-tests: West:   7 = −0.53,    = 0.612, East: 7 = 1.12,    = 0.299)'
It's not clear here what this test is - is it directional testing against 0? It is also not clear in the figure or text what these selection estimates are - selection differentials?

Selection is defined repeatedly (see, e.g., Reviewer 2’s comment #9, *below*, which quotes one of several examples!). Unless specified otherwise, it is reasonable to assume that a t-test tests a mean against zero, but we have now stated this explicitly (lines 107-108).

8. L 112 'which was characterised by evolutionary stasis of clutch size in both West and East'
Although it is technically correct, I think this is a confusing use of this term here given the pitch of the manuscript - it made me think of traits not evolving when there is selection, when in this case there is no evolution and no selection.

The reviewer agrees that what we write is technically correct (stasis is defined as an absence of change, and is agnostic as to the reason), so we have decided not to change this sentence. The confusion of Reviewer 2 stems from their conflation of evolution with adaptation (something we caution against explicitly in this manuscript), and if they avoid this then they may find this sentence more acceptable.

L 116 ' and might therefore might be argued to represent a corresponding 'paradox of change'
Once again  - I see no paradox here. If you were doing this study in a completely isolated population, like with artificial selection in the lab, then the return to pre-experiment would make sense in terms of opposing natural selection. However, doing this experiment in an open population, immigration would be expected to swamp the response after selection stops. So this 'paradox of change' seems like setting up a strawman. This is possibly some narrative device to slowly build up the bigger picture, but given the previous research on this system, I feel this is unwarranted. I think this can much more simply be presented as 'in an open population, we might expect that reversal of evolutionary response after a brief period of selection occurs either due to opposing natural selection or to immigration, and we test between them.'

We point out that elsewhere in their review, Reviewer 2 is fixated on an absence of natural selection on clutch size.

The reviewer sees no evidence for his narrow definition of the paradox of stasis, whereas we argue that we need to take a broader perspective, within depicts his narrow definition as a specific manifestation of a more general phenomenon (a mismatch between inferences of selection and evolution). We will have to agree to disagree on this, but we hope we can all agree that our work is of relevance to those interested in the reviewer’s narrow definition of the paradox of stasis—as supported by the detail of his reviews.

Finally, if it is all so unsurprising to the reviewer, did they predict the fact that the pre-experimental difference did not re-evolve?

This framing also clashes with the introduction, where in the second paragraph, immigration is introduced as a solution to the paradox of stasis, but then there are 3 paragraphs of the results ignoring immigration and focussing on selection. I would suggest a slight re-ordering, so that after considering what happened after the period of artificial selection in terms of natural selection, it is then considered in terms of immigration. As it stands, it then moves to generally looking at general selection on clutch size across the whole period.

As we have explained previously, we take our lead from the general approach in the literature, which continues to ignore the potential contribution of immigration, with very rare exceptions. This still seems to us the strongest way to convince the reader of the merits of our arguments – first following the prevailing approach, then assessing its performance, then assessing a revised methodology.

9. L 125 ' S (i.e. the covariance between clutch size and annual fitness) was an unreliable predictor of interannual genetic change in the breeding population'
This seems like a correlation. As articulated in my last review, both genetic change (and possibly selection) are measured with considerable error. Without taking this into account, the relationship will of course be weak. Measurement error attenuates relationships. One solution is to model this in a meta-analytic framework.

We have already responded to these arguments in the previous round of reviews. We don’t understand what the reviewer means with “This seems like a correlation” and can therefore not respond to this comment. We agree with the reviewer that estimates of genetic change and selection are uncertain, and this is one of the points we make. However, even if the covariance between clutch size and annual fitness provides an imprecise measure of selection *sensu stricto* (as discussed above in some detail), the covariance itself is estimated with minimal error. Finally, uncertainty in estimated breeding values and their derived statistics (i.e., interannual genetic change in a trait) *is* reported.

10. In the previous review, I criticised the lack of error on the selection differential. I can see that the argument is that because all individuals and their reproduction is known in the population, then the within generation change is known exactly. This is similar to when this is applied in an animal breeding context. However, in the context of animal breeding, we also know all the individuals at the next timepoint come from the individuals present (i.e. there is no immigration). I think then comparing this with all the breeding values in the next timepoint is a bit disingenuous. First, some of the new individuals are immigrants, and second there will have been emigrant offspring, so the actual fitness of the breeders is not actually known - only their local fitness (hence selection is actually estimated with error!). In this case, I would argue that you can only apply this to local recruits. This is done in a second step, but as stated above, I think this is part of setting up a strawman argument. I can see that the comparison of the two is useful to demonstrate the impact of immigration to this local change, but this should be presented in a second step.

We argue that global fitness is not relevant to elucidating local evolutionary dynamics. We could perhaps be more concise with how we refer to selection but it is *local* fitness that is relevant to understanding *local* genetic change. That the ‘global’ evolution of clutch size will differ is fundamental to our study (and yet another point that Reviewer #2 has previously argued *against*). Although we therefore don’t model uncertainty in selection (and the reviewer agrees with our rationale for doing so), we *do* model the uncertainty in the breeding values, and the higher uncertainty of immigrant breeding values.

We object to the reviewer referring to this section as ‘disingenuous’ and the characterisation of our proposal of a ‘paradox of change’ as a strawman: it is an extension of the logic that so frequently identifies examples of a ‘paradox of stasis’. Our aim throughout this manuscript has been to provide a clear narrative that takes an interested but not necessarily expert reader through a series of related analyses. This is to some degree a matter of taste—like almost every journal article—but the other reviewers appreciated it. Indeed, earlier versions of this manuscript were criticised for assuming too much prior knowledge of the topic and the study system, and for not explicitly spelling out all steps.

11. The regression of S on R gives the estimated heritability. As said later in the manuscript, selection should be halved (or slope doubled). So the predicted heritability from this method is 0.066 [−0.134,  0.278], including immigrants, and 0.156 [-0.068, 0.378] without. I can't see this being compared against the estimate of h2 presented in supplements 0.226 [0.172, 0.284], which would seem logical to me.

As highlighted by Reviewer 1, the regression of *S* on *R* could be interpreted as the ‘realised heritability’ (divided by two), as our manuscript now notes (lines 144-147). However, as it is important to note this ratio cannot be compared to the heritability estimated in the base population for reasons explained in the manuscript. This was a point made by Reviewer 1 in their previous review, and which led us to develop a new and statistically rigorous approach to comparing observed and predicted evolutionary change.

12. L130 It is correct that the selection could be biased by an environmental covariance, but this then represents a whole other hypothesis for the paradox of stasis, which hasn't been introduced sufficiently. To be perfectly honest, it seems that much of this might be more directly tested with a multivariate mixed model of fitness and clutch size than the roundabout way presented.

As we discuss at lines 150-153, an environmental covariance would result in a mismatch between inferred and predicted response. Because we take an annual approach to selection and evolution, a multivariate model is not an option. Our approach provides an alternative, more flexible but equally valid approach. We designed this study based on the approach typically taken by evolutionary ecologists studying trait evolution in the wild. We feel that in so doing it is more approachable to our audience and that its relevance is made most obvious.

13. L135 - 141. I don't think I really follow this? Is this the same as the year specific heritability calculation in the methods? If so, I would highly recommend labelling it as such, rather than a 'conversion rate'. It's also worth noting there that this assumes that Va is constant, as individuals have a constant breeding value, and so the year specific heritability is being generated by allowing Vp to vary rather than Va.

No. Va is allowed to vary. Individuals *do* have a constant breeding value but the individuals present each year *change*. Dealing with both this and the reviewer’s following comment (#14), we have moved this section to the Methods, where we have expanded our explanation (lines 441-456).

We chose to refer to a ‘conversion rate’ because we feel there is value in reminding readers that this is why heritability is a meaningful quantity to us as evolutionary ecologists.

14. L157 As above, I didn't really understand these statistics, and could link them clearly to a part of the methods.

See response to previous comment.

15. L159 'perhaps owing to prevailing expectations that the evolutionary effect of gene flow is ignorable [33]'
I think this is a misrepresentation. I presume that this refers to part of the questionnaire where researchers were asked 'How important are each of these factors in limiting adaptive responses to climate change', and gene flow was the ranked lowest. Climate change works at much larger spatial scales to what is being described here, and so when we are considering response to climate change, any shift in selection pressure would likely be persistent across populations connected by immigration. Therefore I don't think this can be used to state that researchers generally think that 'the evolutionary effect of gene flow is ignorable'.

We have revised this sentence (lines 160-164).

16. Figure 4 - Given that this is simulated data and not results, it seems like a figure more suited to the introduction, as a demonstration of how immigration can affect observed change. I dont think it adds much to a results section.

It was brought into the main article in response to comments received in the first round of reviews. We included this figure to help the reader understand the decomposition method we are proposing and make the general point that the contribution of immigration is a function of both the size of this demographic and its genetic distinctness. While this reviewer did not find it useful, we know others did. It could be introduced at various places, but we think it works well here, especially since the *Methods* appear at the end of the manuscript.

17. L 212 'This describes the genetic impact of local adaptation'

And drift!!

Yes, and we explicitly highlight this earlier in the manuscript (sentence remains at line 181-183)! We have strengthened this emphasis during revision (lines 123-127, 425-426).

18. L 219 'The latter may be linked to long-term environmental changes in the West creating a post-experimental selection regime that differs from that experienced by earlier generations.'

But is not reflected in the in selection shown in figure 2b

We have deleted this sentence.

19. L 229 'conceals negative selection on clutch size at a genetic level'
Selection is phenotypic, and describes a causal relationship between a phenotype and fitness. There are several forces that may make it seem as though selection is stronger or weaker, e.g. an environmental covariance between a trait and fitness can make it seem as though there is selection when there isn't (as mentioned), or soft selection attenuating selection when hard selection is assumed (although as this is within year selection this wouldn't be an issue here). If there is a genetic relationship but not a phenotypic one, I don't think this provides any evidence for selection. It seems more plausible that there is a genetic correlation with another trait under selection, or this is drift.

The concept of ‘genetic selection’ is widely used and is a useful shorthand for a concept that otherwise requires a lengthy explanation. We must also point out that an environmental covariance between a trait and fitness is expected whenever the heritability is less than unity. The key issue, then, is if the environmental covariance is larger (or smaller) than expected, and not if there is an environmental covariance. We have now clarified that a genetic change that is larger than expected could be the result of selection on a genetically correlated trait (lines 227-229).

20. L269 'While adaptive responses to observed selection on clutch size were directionally consistent with adaptationist expectations, they were, overall, smaller than predicted via application of Robertson's secondary theorem'
I don't think there is any evidence of adaptive response outside of the selection experiment. This is also the first (and only?) mention of the secondary theorem, so I think this either needs to be explained or dropped. I also don't quite see how it comes in here, as the genetic covariance between clutch size and fitness is not estimated (although doing so may simplify some of what is presented).

Although not mentioned explicitly in the Main Text, in the *Methods* we describe in some detail how we derive the expected genetic change from the Price equation (lines 444-456). We have deleted explicit reference to Robertson’s secondary theory in the Discussion (lines 268-269).

21. I haven't reviewed more of the discussion, as I can't see any convincing evidence that there is any natural selection acting on clutch size at any point, but the discussion is written completely in the context of the paradox of stasis.

We explained in our previous revision that we don’t recognise this dichotomous perspective of natural selection (i.e., it is either present or absent). The Discussion’s first paragraph addresses the generality of our findings (lines 268-278), something that the reviewer has questioned, so this paragraph should have been of great interest to them. The second paragraph summarises the potentially important role of gene flow and immigration as a dynamic evolutionary force that goes beyond a source of noise (lines 280-291), thus puncturing Reviewer 2’s belief that they had perfect foresight regarding clutch size evolution.

22. L379 'quantitative genetic statistics are calculated for each sampled iteration and the distributions across all sampled iterations taken to represent posterior distributions.'
I think this needs to be clearer what this refers to. For example, in a comment above, I have written about the correlations between R and S needing to account for error. Now I am not sure from this sentence whether this analysis is included in this, as it is not a quantitative genetic statistic.

The uncertainty that arises from statistical inference of breeding values is included. See further discussion of this topic above.

23. L 391 I'm not sure where this is presented! Whilst this does take into account one source of error, it assumes that the mean in each year within an iteration is also estimated without error, and so some years the sample sizes are not that big.. Each year will also be weighed the same, even though the population sizes in each year may be quite different from each other. I can't fully workout if this is an issue, but a weighted regression for each interaction might help?

The posterior distribution of annual means based on smaller samples size will be wider, which will have the effect of weighting the slope by sample size.

24. L 416 a statistically significant quadratic is not evidence of either stabilising or disruptive selection, rather that there is just non-linearity. There would have to be evidence that the optimum was within the range of the data. I would suggest not referring to stabilising or disruptive selection and rather refer to positive and negative quadratic selection.

Thank you. We have revised this paragraph as suggested (lines 413-423).

25. L 426 'The breeder's equation predicts the phenotypic change from one generation to the next [5]. We cannot use the breeder's equation to predict the genetic change from one year to the next because the breeding population in the next year is partially made up of survivors from the previous year, whose phenotype will be more similar to their phenotype last year than expected based on the heritability of the trait (i.e. due to permanent environment effects). '

This is true for phenotypic change, but not genetic change - the breeders equation with 'annual genetic success' will correctly predict genetic change, as the genetic change doesn't not include the permanent environment effects. If fitness is half fecundity(f) plus survival(s) then:
R = h2S
R = h2 cov(f/2+s,z)
R = h2 cov(f/2,z) +h2 cov(s,z)
In terms of the phenotypic response, this does not work, and the surviving individuals will either have the same phenotype in the case of fixed traits, or a phenotype dictated by the traits repeatability across years, and so scaling the survival component by heritability doesn't work. For the phenotypic change you would want
Delta_p = h2 cov(f/2,z) + cov(s,z)
However it does work for the genetic change, as the heritability also represents the relationship between genotype and phenotype, and so phenotypic selection via survival is scaled by the h2 to get the genetic change:
Delta_a = h2S
Delta_a = h2 cov(f/2+s,z)
Delta_a = h2 cov(f/2,z) +h2 cov(s,z)
As is pointed out, selection would also be halved, as it is only on females.

We agree that we cannot use the breeder’s equation to predict the genetic change to the next year from the phenotypic association between trait and annual fitness, and we provide a method that deals with this. It is not clear what the reviewer wants us to change.

26. L 438 there seems to be a mix of notation with a_bar and mu_a. The mu_a notation has loads of nested subscriptions and becomes hard to follow. Perhaps adopting the a_bar notation throughout would be easier to follow? Given that these are realised values that you are calculating, and not theoretical parameters, I think the a_bar notation is more suitable. Also is there a reason to use x for time, instead of th more traditional t? I think this would also help digest the many subscripts

See our response to comment #29.

27. L 445 is there a couple of 'female breeder's missing from the first covariance?

See our response to comment #29.

28. L446 - I don't understand this - there is an unexplained leap. Unless it is meant to be sigma^2_{a_{female breeder_{x}}} / sigma^2_{*z*_{female breeder_{x}}}, and not  sigma^2_{*w*_{female breeder_{x}}}? I think this might emphasise how difficult it is to keep track of this notation.

See our response to comment #29.

29. L449-L458 This is a nice mathematical demonstration of this, but it is known that heritability is given by the regression of a on z, so it could just be stated, given that there is a fair bit of complexity and notation to follow. It also isn't well linked back to the results, or its relevance made very clear. As noted above, this calculation of year specific heritability assumes a constant va, and so variation in this only comes from changing Vp.

We have switched the algebraic notation from *x* to *t*, as suggested. We also briefly discuss the use of $\bar{a}$ versus $\mu_{a}$(lines 451-452). Furthermore, in response to this comment we substantially shortened and simplified this section.

The final comment about the assumed constancy of V_A_ over time is incorrect, as noted in our answer to Reviewer 2’s minor comment #13.

# Reviewer #3:

The authors have thoroughly and thoughtfully addressed all my previous comments (Reviewer 3) and sufficiently clarified and revised the manuscript. After a careful reading of the manuscript, I do not have any criticisms or comments and think this is an excellent study!

Thank you for taking the time to read our revised manuscript and for your kind words.

# Journal Requirements

Please insert an Ethics Statement at the beginning of your Methods section, under a subheading 'Ethics Statement'. It must include:

i) The name(s) of the Institutional Review Board(s) or Ethics Committee(s)

ii) The approval number(s), or a statement that approval was granted by the named board(s)

We have moved our ethics statement has been moved to the start of the Methods section (lines 311-314).

2) We are unable to open your Supporting Information file [Figure S3]. Please kindly revise as necessary and re-upload.

We have revised Figure 3.
