# Supplementary material for: Gene flow drives periods of both evolutionary stasis and change in a wild bird population
Source: PLoS Biol. 2026 Sep 21;24(9):e3004004. doi: 10.1371/journal.pbio.3004004 (PMC13630318; doi:10.1371/journal.pbio.3004004)
Supplement: Peer review history

# Editor report: Decision Letter 0

**DOI:** 10.1371/journal.pbio.3004004.r001

**Authors:** Roland Roberts

27 Jun 2025

Dear Dr Evans,

Thank you for submitting your manuscript entitled "Paradox lost? Gene flow determines trait evolution in a wild bird population" for consideration as a Research Article by PLOS Biology.

Your manuscript has now been evaluated by the PLOS Biology editorial staff, as well as by an academic editor with relevant expertise, and I'm writing to let you know that we would like to send your submission out for external peer review.

However, before we can send your manuscript to reviewers, we need you to complete your submission by providing the metadata that is required for full assessment. To this end, please login to Editorial Manager where you will find the paper in the 'Submissions Needing Revisions' folder on your homepage. Please click 'Revise Submission' from the Action Links and complete all additional questions in the submission questionnaire.

Once your full submission is complete, your paper will undergo a series of checks in preparation for peer review. After your manuscript has passed the checks it will be sent out for review. To provide the metadata for your submission, please Login to Editorial Manager (https://www.editorialmanager.com/pbiology) within two working days, i.e. by Jul 01 2025 11:59PM.

If your manuscript has been previously peer-reviewed at another journal, PLOS Biology is willing to work with those reviews in order to avoid re-starting the process. Submission of the previous reviews is entirely optional and our ability to use them effectively will depend on the willingness of the previous journal to confirm the content of the reports and share the reviewer identities. Please note that we reserve the right to invite additional reviewers if we consider that additional/independent reviewers are needed, although we aim to avoid this as far as possible. In our experience, working with previous reviews does save time.

If you would like us to consider previous reviewer reports, please edit your cover letter to let us know and include the name of the journal where the work was previously considered and the manuscript ID it was given. In addition, please upload a response to the reviews as a 'Prior Peer Review' file type, which should include the reports in full and a point-by-point reply detailing how you have or plan to address the reviewers' concerns.

During the process of completing your manuscript submission, you will be invited to opt-in to posting your pre-review manuscript as a bioRxiv preprint. Visit http://journals.plos.org/plosbiology/s/preprints for full details. If you consent to posting your current manuscript as a preprint, please upload a single Preprint PDF.

Feel free to email us at plosbiology@plos.org if you have any queries relating to your submission.

Kind regards,

Roli Roberts

Roland Roberts, PhD

Senior Editor

PLOS Biology

rroberts@plos.org

# Peer review: Decision Letter 1

**DOI:** 10.1371/journal.pbio.3004004.r002

**Authors:** Roland Roberts

19 Sep 2025

Dear Dr Evans,

Thank you for your patience while your manuscript "Paradox lost? Gene flow determines trait evolution in a wild bird population" was peer-reviewed at PLOS Biology. Your manuscript has been evaluated by the PLOS Biology editors, an Academic Editor with relevant expertise, and by three independent reviewers.

You'll see that Reviewer #1 is impressed by the dataset and the analyses but finds the paper to be rather confusing and is ultimately not convinced that the two central claims are fully supported by the data. These concerns centre around definition of fast and slow evolution, assigning early survival to maternal fitness, and evidence for the importance of immigration. Reviewer #2 is again broadly positive, but raises similar concerns about lack of clarity, the framing, and the potential for statistical artefact. In his long review he raises a list of more specific concerns. Reviewer #3 is also positive, but raises several concerns about some inconsistencies between aspects of your analytical framework, some assumptions underlying breeding value prediction, biases arising from immigration, and lack of tests for drift.

IMPORTANT: I discussed these reviews with the Academic Editor, and the following cherry-picked comments might prove helpful when you're revising the paper: "It is clear that all three reviewers think this is a really interesting data set and well worthy of publication by PLoS Biol. I agree. I also have the impression that the long list of suggestions for analytic changes are sensible. Maybe it won't be possible to simultaneously satisfy all three reviewers in every instance - especially when there is a choice of approaches... I'd 'reassure' the authors that in cases where the suggestions of the reviewers conflict it is OK to pick one option, but they should be sure to justify why they did not pursue the alternate approach."

As you will see in the reviewer reports, which can be found at the end of this email, although the reviewers find the work potentially interesting, they have also raised a substantial number of important concerns. Based on their specific comments and following discussion with the Academic Editor, it is clear that a substantial amount of work would be required to meet the criteria for publication in PLOS Biology. However, given our and the reviewer interest in your study, we would be open to inviting a comprehensive revision of the study that thoroughly addresses all the reviewers' comments. Given the extent of revision that would be needed, we cannot make a decision about publication until we have seen the revised manuscript and your response to the reviewers' comments. Your revised manuscript would need to be seen by the reviewers again, but please note that we would not engage them unless their main concerns have been addressed.

In addition to these revisions, you will need to complete some formatting changes, which you will receive in a follow up email. A member of our team will be in touch with a set of requests shortly.

We appreciate that these requests represent a great deal of extra work, and we are willing to relax our standard revision time to allow you 6 months to revise your study. Please email us (plosbiology@plos.org) if you have any questions or concerns, or envision needing a (short) extension.

At this stage, your manuscript remains formally under active consideration at our journal; please notify us by email if you do not intend to submit a revision so that we may withdraw it.

**IMPORTANT - SUBMITTING YOUR REVISION**

Your revisions should address the specific points made by each reviewer. Please submit the following files along with your revised manuscript:

1. A 'Response to Reviewers' file - this should detail your responses to the editorial requests, present a point-by-point response to all of the reviewers' comments, and indicate the changes made to the manuscript.

*NOTE: In your point by point response to the reviewers, please provide the full context of each review. Do not selectively quote paragraphs or sentences to reply to. The entire set of reviewer comments should be present in full and each specific point should be responded to individually, point by point.

You should also cite any additional relevant literature that has been published since the original submission and mention any additional citations in your response.

2. In addition to a clean copy of the manuscript, please also upload a 'track-changes' version of your manuscript that specifies the edits made. This should be uploaded as a "Revised Article with Changes Highlighted " file type.

*Resubmission Checklist*

When you are ready to resubmit your revised manuscript, please refer to this resubmission checklist: https://plos.io/Biology_Checklist

To submit a revised version of your manuscript, please go to https://www.editorialmanager.com/pbiology/ and log in as an Author. Click the link labelled 'Submissions Needing Revision' where you will find your submission record.

Please make sure to read the following important policies and guidelines while preparing your revision:

*Published Peer Review*

Please note while forming your response, if your article is accepted, you may have the opportunity to make the peer review history publicly available. The record will include editor decision letters (with reviews) and your responses to reviewer comments. If eligible, we will contact you to opt in or out. Please see here for more details:

https://blogs.plos.org/plos/2019/05/plos-journals-now-open-for-published-peer-review/

*PLOS Data Policy*

Please note that as a condition of publication PLOS' data policy (http://journals.plos.org/plosbiology/s/data-availability) requires that you make available all data used to draw the conclusions arrived at in your manuscript. If you have not already done so, you must include any data used in your manuscript either in appropriate repositories, within the body of the manuscript, or as supporting information (N.B. this includes any numerical values that were used to generate graphs, histograms etc.). For an example see here: http://www.plosbiology.org/article/info%3Adoi%2F10.1371%2Fjournal.pbio.1001908#s5

*Protocols deposition*

To enhance the reproducibility of your results, we recommend that if applicable you deposit your laboratory protocols in protocols.io, where a protocol can be assigned its own identifier (DOI) such that it can be cited independently in the future. Additionally, PLOS ONE offers an option for publishing peer-reviewed Lab Protocol articles, which describe protocols hosted on protocols.io. Read more information on sharing protocols at https://plos.org/protocols?utm_medium=editorial-email&utm_source=authorletters&utm_campaign=protocols

Thank you again for your submission to our journal. We hope that our editorial process has been constructive thus far, and we welcome your feedback at any time. Please don't hesitate to contact us if you have any questions or comments.

Sincerely,

Roli Roberts

Roland Roberts, PhD

Senior Editor

PLOS Biology

rroberts@plos.org

-----------------------------------------

REVIEWERS' COMMENTS:

Reviewer #1:

This multi-decadal study of clutch size evolution in nest box populations of great tits addresses two fundamental questions in evolutionary biology. 1) Why do we often see an absence of an evolutionary response to measured natural selection on heritable traits (paradox of stasis)? 2) How important is gene flow relative to other evolutionary processes to overall rates of evolution. There are two foundational, timely and important questions in the field and the latter question in particular is very understudied. The dataset used to address the question is terrific and the analyses are sophisticated. The manuscript and writing are generally clear, but there were a few places where I was confused - either by the complexity of the material being presented or the way in which the material was presented.

Overall the study makes two overall claims. First, that an overall correspondence between evolutionary predictions and observations can result from episodes of under- and over-prediction. Second, that gene flow is important to evolutionary dynamics in this system. Unfortunately, I was not convinced by the data supported these two important conclusions.

It seems a certainty that the slope for 'slow' years will be lower when 'slow' defines the range on the y-axis to be more constrained. I think the observed patterns in 3b will always be the case - or at least I would like to see some sort of null model or simulation to asses the degree to which these patterns are pre-determined. It is possible here that I have misunderstood how period of fast and slow evolution were defined. If so, then more clarity is needed here. I also worry that there is a potential confound between the strength of selection associated with the degree of certainty that the appropriate target of selection has been identified. Selection was measured as a selection differential, which can be confounded by indirect selection on other traits. This is more likely in years of 'natural selection' and less likely in years with 'artificial selection'. The latter also appear to have stronger selection differentials. So the strength of selection is likely confounded by the certainty with which clutch size is the target of selection.

Overall, the selection differential measures are also likely problematic because selection on the maternal trait is based on a fitness measure that includes offspring early survival. Thomson and Hadfield discuss the risks associated with assigning early survival to the fitness of their mother, particularly for the prediction of rates of evolutionary change. There are circumstances under which these risks are lower, but this needs to be carefully assessed and reported. One of the conditions is that the parental trait does not affect parental survival, but this does not seem to be the case here since breeder mortality seems to be an important driver of changes in clutch size breeding values in Figure 4.

I was also unconvinced of the importance of immigration to evolutionary dynamics as shown in Figure 4 and Table S5. I tried very hard, but could not figure out what metrics or items in these depictions led to the inference that immigration is the driver of evolutionary dynamics. I also have worries about the ability of standard animal models to assess the importance of immigration to evolutionary dynamics when they are based on a homogenous base population. Reid and Wolak (and others) have done some elegant work to demonstrate the value of animal models with heterogeneous genetic groups as a way to explicitly model the impacts of gene flow through immigration (one citation in this manuscript; #18). I am not convinced that the current approach, which assumes that immigrants and residents come from a common base population adequately reflects the biology that the authors are attempting to study. I think to explicitly test the importance of gene flow, genetic group models seem necessary to allow immigrants to come from a different base population. This issue ought to be explicitly addressed.

Specific Comments

- line 17. I struggled with the statement that evolutionary responses to selection were smaller than predicted in years of evolutionary stasis. Was the relationship between prediction and response not used to define which years involved stasis? Do you simply mean that in some years the rate exceeded predictions and in some years it was lower than predictions? Stasis is perhaps not the right word here because this implies (to me at least) a lack correspondence between observed and predicted and not just the magnitude of either.

- lines 301-302 regarding thinning interval did not make sense to me. I wondered whether some words were missing.

- how are 'breeders' defined with respect to males? All males in the population at that time?

- How were years of 'fast' versus 'slow' annual responses defined? Were these defined for predicted or observed rates? Were these based on absolute values of rates, or were years of negative evolutionary rates defined to be 'slow' if if it were a rapid evolutionary reduction in clutch size? I assume the former (absolute values), but this was not clear.

- lines 334-338. In terms of notation, I don't think the subscript SC adds clarity since selection was only measured on one trait. The subscript k was included in line 334 but lost in the equation on line 338. Since selection was measured each year, but heritability was measured overall it would be helpful to add subscripts of t and k to R and S to clarify that these were year- and population-specific.

- line 121. I don't think this conclusion that flawed measures of selection are to blame for the stasis is supported. There are many possible explanations for the patterns but the absence of year-specific heritabilities does not imply that any of the other remaining explanations are more likely than others.

-I was unable to understand the section on the calculation of recruits through natives and immigrants (lines 375 - 381).

Reviewer #2

[identifies himself as Joel Pick. IMPORTANT: Please see attachment for fully formatted version]

This study looks at a 44 year dataset of two island populations of great tits, that initially differed in clutch size, a key life history trait. The study charts the changes in breeding values for clutch size through 15 years pre-experiment, then an 8 year artificial selection experiment to reverse this difference, and then for 20 years post artificial selection. This study is done in the context of earlier work on the initial differences between the populations, including the roles of local adaptation and immigration (Postma and Van Noordwijk 2005, Postma et al 2009), and the selection experiment itself (Postma et al 2007). In this study, the authors seek to attribute the differences and changes in breeding values between populations across these time periods to the relative influences of selection and immigration. This study uses a remarkable dataset, to address an interesting, timely, and largely ignored question in wild quantitative genetics.

Generally the writing style is very clear and easy to follow, although in several places I felt I needed more information, and particular parts of the method need clarification. I also felt it could have been put more firmly in the context of the previous work, which already established the important role of immigration in this population. I don’t think that this previous work diminishes the current contribution, but not knowing the previous work well meant that part of the results were hard to contextualise.

I have some major comments that are maybe due to my own ignorance or misunderstanding, but could at least do with some additional discussion in the manuscript. In particular, as there is a lot of complex analysis, which often using estimates from several different models, there is opportunity for patterns to arise due to statistical artefacts - it would be nice to be reassured that this wasn’t the case, or what the potential for these artefacts to arise was. I apologise for the length of the review, but this is the most interesting manuscript I have reviewed in a long while.

Major Comments

1. Overall framing

This is clearly up to the authors, but I find the overall framing a little confusing. I have a bit of a stumbling block over how the paradox of stasis fits in here. It seems like the starting point/framing of the manuscript is selection/local adaptation, and why we don’t find change in the presence of strong, persistent directional selection (paradox of stasis). Whilst I would a priori think that clutch size was under strong, persistent directional selection as it is a component of fitness (although various previous studies have suggested that it is under stabilising selection in tit populations), it doesn’t appear to be under clear selection in these populations at all (see also Postma and Van Noordwijk 2005). To my mind, for there to be local adaptation in two close populations, there needs to be evidence of differing optima, or consistently different directional selection regimes in the two populations. Based on figure two and table S2 (and Postma and Van Noordwijk 2005), there doesn’t seem to be clear evidence of directional selection on clutch size in either the same or opposite directions in the pre-experimental period. From the limited description on l318-328, it also seems like there is no clear stabilising selection, and no mention of differing optima between populations.

The previous work on this population seems to show that local adaptation isn’t the reason for the pre-experimental difference between populations, rather it is due to differential immigration from the mainland into the two island populations, with the mainland immigrants having larger eggs than the island birds, resulting in a larger egg size in the West population with greater immigration (Postma and Van Noordwijk 2005). This information seems lacking from the introduction, results and discussion, although a key bit of context for the framing of the study.

If we are then not looking at a trait that is under strong, persistent directional selection, or with clear evidence of divergent optima between populations, it seems strange to phrase this so heavily in the context of the paradox of stasis. I’m not sure that this study, although a valuable demonstration of the role of migration and gene flow in microevolution on small spatial scales, tells us anything about resolving the paradox of stasis, as it seems in this case we would predict stasis through the lack of selection. Or at least, if it does, I think it needs far better signposting to these results.

The question may then be what happens when we place a trait under strong selection in the wild, and how do different processes (such as immigration) affect selection response. During the period of artificial selection, there was a response in the breeding values (Postma et al 2007, and this study), which shows that strong directional selection in these populations would have a response. When the selection stops the response is then swamped by immigration. This is phrased as a ‘paradox of flux’ (l 98), but I am not clear as to why there is a paradox. Given the generally high immigration rates in tit populations (and in this population, shown Figure S3), is it surprising that immigration is a major driver of return to a previous state after selection ends? I’m just thinking here that, theoretically, if I took two open subpopulations, and performed an artificial selection experiment on a selectively neutral trait in opposing directions, then given immigration rates of 20-50% (figure S3), I would expect a rapid return to mean trait value when selection stopped. If the point is that this is what we would expect, but this is not typically considered by researchers (with which I totally agree), then I think that the introduction should lean on this much more and articulate this prediction, rather than this result being surprising (‘paradox of flux’).

I am not saying that an experimental demonstration of this effect is not very important, interesting and useful, I’m just wondering whether, giving the existing knowledge of the system, would anything else have been expected. The impression I get from the manuscript is that this is somehow unexpected. The paradox of stasis, for my perspective, relates to traits under strong, persistent directional selection, for example juvenile body size.

For me then, it is not super clear how this study shows that immigration is working against selection in this population. Upon reading this several times, I think that a key paragraph is l145-157, but I don’t think that the importance of this results is clearly signposted, or put well into the context of the previous work on the system. Here the demographic decomposition shows a reasonable impact of immigration in the western population, but not the eastern, during the experimental period. I think this would be expected, given the previous work on immigrations into the west and not the east, but this is not clearly articulated. There is then the effect of immigration in the legacy period in the east population, but these effects seem relatively small and uncertain, and it is difficult to separate the effects of drift (although there does seem to be phenotypic selection for larger clutches here, but I don’t think that this is statistically demonstrated anywhere).

Again the framing is up to the authors (and I am happy to be shown wrong), but my suggestion would be to put this much more firmly into the context of the previous work, and make predictions on how you would expect immigration and selection to balance each other in the two populations, given what is already known about immigration and clutch size in the system.

2. Results presented in Figure 3

There are flagged as a major results in the abstract and discussion. However, there is not enough information in the methods to access how this was done, and whether and how the uncertainty was propagated, and so how much weight can be put into these results.

First, it seems that the lack strong correspondence between the annual prediction from the breeders equation (hard to estimate because of large uncertainty in both heritability and selection differential) and the estimated genetic response (from estimates derived from an animal model with high uncertainty), in non-experimental years where selection seems to be stochastically fluctuating around 0 (and appears not to have been shown to be different from zero), leads to the conclusion that the breeders equation doesn’t work. From eyeballing the graph, most of the credible intervals seems to overlap the 1:1 line. For me, this figure provides pretty compelling evidence that the breeders equation does a pretty good job!

Second, something about the results in Figure 3b screams of regression to the mean, although I am happy to be convinced otherwise. The results here do not appear to explained anywhere in the methods, so I do not know whether uncertainty is taken into account. What worries me here is that there is higher uncertainty in the western populations (presumably due to smaller population sizes), and so more spread at the negative quarter of figure 3b than in the positive quarter (more dominated by eastern populations). This sampling variance creates the appearance of heteroscedasticity, which would lead to two different slopes being estimated when splitting the data by whether it is above or below the 1:1 line. Finally, it is worth noting that, although I understand the reason for this approach, the fitness metric used goes from recruit to recruits, and so represents a ‘mixed’ fitness measure (see Thomson & Hadfield 2017 MEE), which can be problematic. There is less risk using this as clutch size is is a sex limited trait, but it would be worth discussing the potential issues with using this measure (as explain in Thomson & Hadfield 2017 MEE). The fact that there is still a good correspondence between predicted and observed response despite using a potentially problematic fitness measure therefore seems even more in the favour of the breeders equation doing an OK job, rather than a bad one.

3. In neither this, or the earlier paper (Postma et al 2007), is there a mention of how the selection experiment affected population density. Perhaps I am wrong, but it seems likely that by allowing only around half the birds to breed, you would affect population size and so lower the breeding density of the birds quite fast, and this might additionally prompt more immigration. Whilst I realise that there is a lot of stochasticity in this, it is interesting that in Figure S2 the particularly high years for immigrants in the West occur during the experimental period. Given that the most interesting results in this paper seem to come from a shift in immigration across time, I feel this needs to be addressed. It would useful to at least include some figures showing the NUMBERS of locally recruited breeders, island breeders, and mainland breeders in both populations over the time period.

4. In an animal model, unknown immigrants (or founders), are sampled with a mean breeding value of 0 and variance VA. If they don’t have multiple female recruits in the population (i.e. phenotyped descents), then there is no (or very little if they have only one female offspring, or female grand-offspring etc) information about their deviation from 0. As I imagine many breeders have no female recruits, this would mean that there are a large bunch of immigrants that by definition have a mean breeding value of 0. Although it is not clear what kind of proportion that might be, it seems significant for many of the results. For example, Figure 5 shows that breeders with no known grandparents have a mean breeding value of 0, and Figure S3 shows that the mean immigrant breeding value fluctuates closely around 0 and doesn’t change over time – I think this is likely simply a consequence of the assumptions of an animal model.

It would be helpful if there was some exploration of this, and some assurance that the impact of immigrants on the mean breeding value of a population isn’t just because a load of individual with no information about the breeding value join the population, and essentially cause regression to the mean. In a similar vein, I think it would be very useful to distinguish between immigrants from the other island population (with parentage information), and unknown immigrants (mainly from the mainland?). For example, is the return from selected clutch sizes during the post-experimental phase due to immigration from the mainland, or immigration between island populations?

This might be due to a lack of knowledge about modelling multiple populations in the same animal model, but I am also slightly uneasy that the breeding values from both populations converge upon zero in the last decade, where it seems like there would be the most information. From discussing this with a colleague, I think it would be appropriate to use genetic group models here, with groups for west, east and immigrant populations, and allow heterogeneous variances across them. This would seem prudent given that, in the early years at least, the east and west populations seem quite distinct, and there is clearly an impact of immigrants arriving with potentially different bedding values.

Within this context, it would also be useful to have more information about how the pedigrees form east and west overlap, in other words how much migration is there between populations, and so how linked are the two pedigrees? I feel a little out of my depth here, but I would worry if they don’t overlap at all, in terms of making inferences from the same model. Having looked at the earlier papers, its seems that this is a reasonable amount (around 10%), but it would be helpful to clarify here.

Specific Comments:

Abstract

5. l 15 - ‘To resolve this’ - perhaps ‘address’ would be more suitable, I don’t think this study can claim to single handedly resolve the paradox.

Intro

6. l 28-38 Very nicely written paragraph!

7. l 42 ‘Besides the stochastic element introduced by genetic drift, immigrants are likely to be genetically distinct from their host population, such that gene flow will also shape a population’s genetic characteristics.’

This seems a bold claim, so I think this needs a reference. Whilst I think it is completely plausible that immigrants are genetically distinct, it really depends on the structure of the wider population. In mainland tit populations for example, I find this relatively hard to believe, a priori.

8. l 43 ‘Indeed, even very occasional arrivals can be highly influential on the host population’s evolutionary trajectory [17,18]’

True in these examples, but these are very small island populations.

9. l 45 - ‘immigration rates often covary’ - perhaps ‘can covary’, I’m not sure enough work has been done on this to say its common?

10. l 58 ‘Despite ongoing gene flow, both genotyping [36] and quantitative genetic modelling [35] has shown that breeders in western and eastern areas are genetically distinct.’

The introduction needs more of a summary of previous papers on this system. For example, it has previously been shown that differential immigration into the west from the mainland has seemingly led to this difference.

11. l 67 ‘assess the predictability of pre- and post-perturbation adaptation’ and ‘local adaptive processes’

I don’t think there is any evidence of adaptation here?

Results

12. l 71-81 It would seem prudent when discussing the pre period, to discuss the selection regimes. From Figure 2, it seems as thought there is little overall selection in either east of west, with it fluctuating around 0. Figure 2 doesn’t show any error on these estimates, so it is difficult to assess this. I think a random regression approach, showing whether there is any overall selection, and whether there is variation in selection across years in the two populations would be helpful. It would also be interesting to know whether there was stabilising selection acting on these two populations. If the difference was due to local adaptation, I would expect that either there would be fluctuation in selection with no overall directional selection (indicative of being at an optima - which could equally be estimated with a quadratic function) or directional selection in opposite directions.

13. l 74 I think it is interesting and worth noting that around 80% of the difference between populations appears to be environmental. The wording makes it sounds like the genetic difference was the major driver.

14. l 79-81 This could be more clearly explained, it took me a couple of goes to get it.

15. l 84 ‘when natural selection operated without experimental interference’

There doesn’t seem clear evidence that there was any natural selection!

16. In l 86, the ‘adaptationist expectation’ is mentioned. However, at this point from looking at figure 2 (and based on Postma & van Noordwijk 2005), I have no expectation that this is adaptation, as there generally appears to be no differences in selection in either population.

17. l 113 P (bfast > bslow)

In the first paragraph of the results there is just posteriors and credible intervals, then in the second paragraph there is frequentist p values, and then here there is posterior probabilities. These are essentially 1-pvalue, so I think it would be worth explaining this, as I was caught of guard, and thought this was wrong, and I think so would other readers.

18. l 105 As outlined in a comment above, I am sceptical of this, perhaps mainly because of the lack of explanation of the methods used. It feels like stats on stats on stats, and I hope the uncertainty has been appropriately propagated all the way through.

19. l 131 I think the distinction between unknown immigrants and ‘local’ immigrants from the other island population would be helpful, and might reveal interesting patterns.

20. l 142-143 The posterior probabilities are presumably calculated as the proportion of iterations. I wonder if it would be worth presenting quantile based credible intervals instead of HPD intervals to make them comparable? I imagine it doesn’t make much difference, just maybe slightly more consistent.

21. l 149-153 This is a very hard sentence, I would suggest rephrasing.

22. l 156 ‘first indication’ - apart from two papers showing differential immigration into the two areas, in a pattern that would predict the observed difference!

23. l 176 - this is the first mention in the manuscript of the fact that immigration comes form the mainland population, and in fact the only mention of this in the whole manuscript. I think this shift in the pattern of immigration is a really key point in this study, with the potential to be driving all of the results.

24. l 179 - As said above, I think this has to be true due to how the animal model works.

Discussion

25. l 189 ‘In an attempt to resolve the paradox of stasis [7,9], we assess how consistently the breeder’s equation— which has proven very reliable for guiding artificially selected evolution of captive [41] and free-living [30,42] populations—fails to predict adaptive trait evolution across a nearly half-centurial study of two, neighbouring, breeding populations.’

I disagree that this study shows that the breeders equation doesn’t work. In the period of strong artificial selection, the is strong concordance between predicted and observed response, but in other years, where there is no demonstrable selection, it doesn’t do well at picking up response. Personally, I don’t think this sheds light on the utility of the breeders equation in the wild.

26. l 196 ‘Thus, if efforts to quantify adaptive responses are made only when traits are a priori known to be under strong selection, then the apparent ubiquity of the paradox of stasis is attributable to observational selection bias’

I’m not sure I get this. So the argument here is that in a trait under no clear directional selection, where immigration dominates, we can find evidence for what resolves the paradox of stasis over/as much as in populations/traits where there clear strong, persistent directional selection but no response?

27. l 211 ‘we show that the genetic impact of immigration is central to understanding contemporary evolution of a key life-history trait, being at least as impactful as natural selection’

In this example! Whilst this might well be true in other cases, I think care should be taken with generalization

28. l 227 ‘relative isolation does not nullify the evolutionary impact of immigration on traits’ evolutionary trajectories’

I would argue that relative isolation was likely to massively amplify the evolutionary impact of immigrants, as the two populations are more likely to be genetically distinct. In a very panmitic situation, I would expect immigration to have much lower impacts, as the genetic differentiation between populations was lower.

29. l 231 - I completely agree

Methods

Generally I think a lot more information if needed throughout the methods to make this easier to follow.

Study system

30. What proportion of the island population is caught every year? Do the nest boxes in the woodland cover the majority of the population? What I am really getting at here is that are ‘unknown’ individuals assumed to be all from the mainland, or is there a substantial unringed island population.

31. I think there needs to be more discussion of the second clutches. What proportion produce second clutches? How much are these contributing to recruitment (and so fitness as measured here)? Are the recruits in the analysis just from first clutches? Does the likelihood of second clutches differ between populations? Did the selection regimes affect second clutches?

32. l 257 - I agree that it is unlikely, but presumably you could check whether this had carry over effects on the following years reproduction?

Quantifying Va

33. l 283 - in reference to an comment above - about a third of individuals are founders. It would be good to know how many of the phenotyped ‘founder’ females had females recruits.

34. l 288 there is a rogue brackets towards the end of the line.

35. l 300 Thank you for including some kind of sensitivity analysis

36. l 301 ‘A thinning interval of 10,000 correlations among successive samples of <0.03 for all random effects’ I think there is a missing word somewhere

Quantifying evolutionary change

37. Should the means be weighed by SE or 1/N within each regression? I don’t know how much population size varies across years?

Quantifying selection

38. As mentioned in another comment above, this is a recruit-recruit measure of fitness which mixes fitness of parent and offspring. It would be good to acknowledge the potential issues this can cause in terms of estimation of selection (see Thomson and Hadfield 2017 MEE). For example, maternal clutch size might well indirectly affect offspring survival as it affects juvenile size.

39. l 318-328 - This could be much clearer, and I think these results and a much clearer methodology should be presented in the supplements. It is not clear what models were run. If individual linear regressions on relative fitness (relativised by dividing by the mean), then I am not surprised that sometime disruptive selection was found, as there will be clear non-linearity in the fitness function, due to the limit at 0, which will appear like disruptive selection. This could also cloud stabilising selection. Something like a Poisson GLM seems much better suited for this, as it accounts for this inherent non-linearity. It would also be very informative to do a random regression analysis here, looking for the overall direction of selection in each population (possibly per period) and whether it differs between populations (as an interaction), and whether there is detectable variation in selection across years. There is an implicit assumption in the breeders equation analysis that directional selection varies across years, but this never seems to be tested. This cannot be assessed from Figure 2, as there is no uncertainty plotted, and if they are estimates from individual models, then the variance among them will be inflated.

Predicting and inferring local adaptation and Figure 3

40. There is unfortunately not enough information presented here to assess the methodology. It is not clear a) whether uncertainty in selection was quantified and incorporated into the uncertainty on the annual predictions of evolutionary change b) whether the uncertainty in both the annual predictions and the inferred responses was incorporated into the regression (e.g. in a bivariate meta-analysis), c) how the analysis in in Figure 3b was done, as it is not even mentioned in the methods - was this a single model, testing the interaction with the uncertainty incorporated? If not, my concern is that this result is due to sampling variance.

41. It is worth noting that the sensitivity analysis with varying h2 per year doesn’t allow the Va to change per year - that is fixed in the animal model - it is essentially allowing the environmental component to vary. So whilst this is a useful additional, it might also be that there is GxE, which this doesn’t test.

42. Very minor point, but the ‘CS’ subscript on the S seems unnecessary sine there is only selection on clutch size. When I first read it I thought it was an index.

Decomposing evolutionary change

43. l 348 ’ democratic’ - presumably demographic?

44. I find this part hard to follow, and still can’t work out whether the logic of l 375-381 works, or whether the resulting sum of components will give back the correct change. The contribution to a current years mean can also be thought of as the weighted averages of the different groups. It would seem that this much much easier to calculate and explain:

¯a = psurvivors̄asurvivors + precruits̄arecruits + pimmigrants̄aimmigrants

where p is the proportion. The contribution of group x can then just be px̄ax. Perhaps there is a reason why this wouldn’t work and the other method would, but as it stands I don’t understand the current method!

45. It would be interesting to also separate to within island from mainland immigration.

46. l 369-373 The relevance of this information for this section is not clear to me.

47. Figure 5 is never described in the methods, and only referred to in passing in the results.

Data Availability

48. Data and code doesn’t seem to be available for review (at least the provided DOI didn’t work for me). I think this is a shame, as it would have helped me work out what was done in several places where the methods were unclear (especially in the selection analyses), which would save a bit of back and forth in review.

Reviewer #3:

The manuscript "Paradox lost? Gene flow determines trait evolution in a wild bird population" is a timely work that goes beyond the typical approach in a free-living population to explaining evolutionary change based solely on response to natural selection. This manuscript attempts to add gene flow to the evolutionary processes responsible for genetic trends through time by capitalizing on an impressive experimental manipulation conducted within a very long-term dataset on free-living birds.

While incorporating evolutionary change due to gene flow into prediction of phenotpyic evolution from wild populations is an area of great interest currently, there are several aspects to the analysis, approach, and interpretation that need clarification and justification. I elaborate on these main comments and then include some smaller, more specific comments at the end.

## Main Comments

### 1. Evolution, demography, and selection

1.1. The authors did not justify the simultaneous use of a framework for prediction from evolutionary quantitative genetics (QG) and several metrics that are not consistent with that framework. Specifically, evolutionary QG theory (e.g., breeder's equation) separates natural selection from inheritance to make predictions about evolutionary change, but the measures of selection and descriptions of demographic changes in genetic value include some individuals across timepoints. This fact that an individual survives and so resembles itself between time periods, conflates selection and inheritance in a way that is not consistent with the QG theory for how inheritance is modeled.

1.1.2. A specific example of point 1.1 comes from contrasting the breeder's equation prediction about change over a generation with the timeline over which the manuscript evaluates changes - namely, each year (in an animal with overlapping generations). The heritability used in the breeder's equation comes from the additive genetic and phenotypic variances estimated in the animal model (Lines 331-344). However, selection is estimated using an individual's own survival as part of its fitness measure. Without year-specific breeding values then the breeder's equation prediction has an inter-generation measure of heritability combined with a measure of selection that uses both intra- and inter-generational change. Consequently, the observed response to selection includes the genetic value of many individuals that occur in both time points for the response to selection (e.g., timepoint x and x+1). This is then not consistent with the heritability measure. Specifically, the breeder's equation will fail to correctly predict the change in genetic merit when the current and future timepoints include a large overlap of individuals.

### 2. Breeding value prediction

The manuscript relies heavily on the predicted breeding values from the animal model to demonstrate genetic trends through time and evaluate proposed dynamics for how natural selection and immigration will cause these trends in genetic values. Therefore, un-biased breeding value predictions are critical. There are a couple of key points that need to be considered.

2.1. Estimation of mean breeding value for groups (e.g., timepoints) especially need to control for environmental differences in mean phenotype between groups. The authors include woodlot identity as fixed effects in the model, but this does not control for environmental differences across years that affect the mean phenotype (and hence mean breeding value). Environmental differences (Hadfield et al. 2010) or trends (Sorensen et al. 2001) have a great impact on inferences using breeding value predictions derived from quantitative genetic mixed models. Temporal changes in the environment should be removed in order to obtain an environment-independent prediction of breeding values. The solution, then, is to include year-by-woodlot fixed effects in the model.

Sorensen, Fernando, & Gianola. 2001. Inferring the trajectory of genetic variance in the course of artificial selection. Genet. Res. Camb. 77:83-94. DOI: 10.1017/S0016672300004845

Hadfield, Wilson, Garant, Sheldon, & Kruuk. The misuse of BLUP in ecology and evolution. The American Naturalist 175(1):116-125. DOI: 10.1086/648604

2.2. Additive genetic variance will be biased when immigrant groups are not specifically recognized in the analysis yet differ on average from the residents (Wolak & Reid 2017). Biased additive genetic variance estimates will cause problems for breeding value prediction since such predictions assume the additive genetic variance is known (see Sorensen et al. 2001 referenced above). This is potentially a problem with the current analyses, given the authors argue that later in the experiment immigrants carry alleles that shift the mean breeding values and there is a substantial proportion of individuals for whom the natal origin is unknown (e.g., Figure S2). Unless specifically treated otherwise, individuals of unknown origin and unknown parentage will be added to the pedigree "founder population". Additionally, there is the further risk that breeding value prediction will be heavily influenced by phenotype particularly for individuals with unknown parents that do not have many offspring with phenotypes. Consequently, an increasing proportion of individuals with unknown origin (e.g., Figure S2) could mean that the trend in breeding values is driven by phenotypic change which is not genetic change. This would support the importance of gene flow, but at the phenotypic and not genetic level. The phenotypic and genetic levels will diverge particularly if there are environmental trends through time (which the authors note).

Wolak, M.E., & J.M. Reid. 2017. Accounting for genetic differences among unknown parents in microevolutionary studies: how to include genetic groups in quantitative genetic animal models. J. Anim. Ecol. 86:7-20. doi: 10.1111/1365-2656.12597

### 3. No tests for the effect of drift

Though this is recognized (e.g., Line 133), it is surprising that some form of the Hadfield et al. 2010 method of simulating expected changes in breeding values due to drift was not implemented. Without this, the changes in mean genetic merit cannot be attributed to either natural selection or immigration without drift being a plausible null hypothesis.

## Detailed Comments

4. Lines 88 to 93: The authors do not consider uncertainty in selection estimates when aggregating over them to test the significance or difference between groups.

5. Lines 95 to 98: The authors describe the conclusions about the system by contrasting a situation of little to no adaptive evolution despite strong selection to the observation from their results of genetic change/evolution in the absence of strong natural selection. It is unclear whether the statement about no adaptive evolution despite strong selection refers to the general paradox of stasis or whether they are invoking this to summarize the pre-experimental period. Either way, such conclusions seem at odds with the results that there are no differences in the strength of selection when comparing the pre- and post-experiment periods (My comment 4. Lines 88 to 93) - though see my other comment about this result.

6. Line 266: Please expand to clarify this sentence.

7. Line 288: There is a stray ")" after "effect".

8. Lines 297 to 299: I do not think the description of this prior is correct. For example, as the prior was coded with `alpha.nu = diag(1) * 1000` that does not refer to the prior variance but a hyperparameter on the variance. The prior was specified `G1 = list(V = diag(1), nu = 1, alpha.mu = 0, alpha.V = diag(1) * 1000)` so it would be more informative and perhaps more correct to say this prior is a "... parameter expanded prior that is relatively uninformative as it closely approximates the scaled non-central F-distribution with numerator and denominator degrees of freedom equal to one and a scale parameter of 1,000 (Gelman 2006; Hadfield 2010)."

Gelman A. 2006 Prior distributions for variance parameters in hierarchical models

(comment on article by Browne and Draper). Bayesian Anal. 1, 515-534.

(doi:10.1214/06-BA117A)

Hadfield J. 2010 MCMC methods for multi-response generalized linear mixed models:

the MCMCglmm R package. J. Stat. Softw. 33, 1-22.

(doi:10.18637/jss.v033.i02)

9. Lines 301 to 302: This sentence seems to be missing part of the middle between "10,000" and "correlations". Also, are the auto-correlation values for the random effects of the variance of random effects?

10. Line 348: "democractic" should be "demographic"

Attachment Submitted filename: review.pdf

# Author comment: Author response to Decision Letter 2

**DOI:** 10.1371/journal.pbio.3004004.r003

31 Mar 2026

Attachment Submitted filename: Response to reviewers comments.pdf

# Peer review: Decision Letter 2

**DOI:** 10.1371/journal.pbio.3004004.r004

**Authors:** Roland Roberts

21 May 2026

Dear Dr Evans,

Thank you for your patience while we considered your revised manuscript "Paradox lost: Gene flow drives periods of both evolutionary stasis and change in a wild bird population" for consideration as a Research Article at PLOS Biology. Your revised study has now been evaluated by the PLOS Biology editors, the Academic Editor and the original reviewers.

You'll see that reviewer #1 is generally pleased with the revisions, but wants you to draw out a couple of aspects of the study more clearly. Reviewer #2 is also positive about the re-worked paper. However, he still struggles with the framing and wonders whether some re-structuring might help (and he’s sceptical that the paper presents a solution to the “paradox of stasis”). He then has a list 29 (!) minor points. Reviewer #3 is now fully satisfied and has no further requests.

I discussed the rather substantial nature of reviewer #2's remaining requests with the Academic Editor, who said "I think it is appropriate to ask the authors to attend to any specific technical challenges posed by reviewer #2 - for example, Point 11 claiming that a value should be doubled; but we are in the realm of personal preference and perfectionism when it comes to suggesting how to frame the study... I think it is obvious that Evans et al have extensively rewritten the MS in the light of feedback and done their best. My view is that the data and analysis (and to a lesser extent the interpretation) are the most crucial parts of a paper. It is clear from the comments from all three reviewers that they think this is an interesting study with good data and interesting analyses. So, I'd give the authors once more chance to tidy up loose ends and double check the technical queries. You should consider reviewer #2's suggestions for reframing but acceptance will not be contingent on complying with the more stylistic requests." (lightly edited)

In light of the reviews, which you will find at the end of this email, we are pleased to offer you the opportunity to address the remaining points from the reviewers in a revision that we anticipate should not take you very long. We will then assess your revised manuscript and your response to the reviewers' comments with our Academic Editor aiming to avoid further rounds of peer-review, although we might need to consult with the reviewers, depending on the nature of the revisions.

In addition to these revisions, you may need to complete some formatting changes, which you will receive in a follow up email. A member of our team will be in touch with a set of requests shortly. If you do not receive a separate email within a few days, please assume that checks have been completed, and no additional changes are required.

We expect to receive your revised manuscript within 1 month. Please email us (plosbiology@plos.org) if you have any questions or concerns, or would like to request an extension.

At this stage, your manuscript remains formally under active consideration at our journal; please notify us by email if you do not intend to submit a revision so that we withdraw the manuscript.

**IMPORTANT - SUBMITTING YOUR REVISION**

Your revisions should address the specific points made by each reviewer. Please submit the following files along with your revised manuscript:

1. A 'Response to Reviewers' file - this should detail your responses to the editorial requests, present a point-by-point response to all of the reviewers' comments, and indicate the changes made to the manuscript.

*NOTE: In your point-by-point response to the reviewers, please provide the full context of each review. Do not selectively quote paragraphs or sentences to reply to. The entire set of reviewer comments should be present in full and each specific point should be responded to individually.

You should also cite any additional relevant literature that has been published since the original submission and mention any additional citations in your response.

2. In addition to a clean copy of the manuscript, please also upload a 'track-changes' version of your manuscript that specifies the edits made. This should be uploaded as a "Revised Article with Changes Highlighted " file type.

*Resubmission Checklist*

When you are ready to resubmit your revised manuscript, please refer to this resubmission checklist: https://plos.io/Biology_Checklist

To submit a revised version of your manuscript, please go to https://www.editorialmanager.com/pbiology/ and log in as an Author. Click the link labelled 'Submissions Needing Revision' where you will find your submission record.

Please make sure to read the following important policies and guidelines while preparing your revision:

*Published Peer Review*

Please note while forming your response, if your article is accepted, you may have the opportunity to make the peer review history publicly available. The record will include editor decision letters (with reviews) and your responses to reviewer comments. If eligible, we will contact you to opt in or out. Please see here for more details:

https://blogs.plos.org/plos/2019/05/plos-journals-now-open-for-published-peer-review/

*PLOS Data Policy*

Please note that as a condition of publication PLOS' data policy (http://journals.plos.org/plosbiology/s/data-availability) requires that you make available all data used to draw the conclusions arrived at in your manuscript. If you have not already done so, you must include any data used in your manuscript either in appropriate repositories, within the body of the manuscript, or as supporting information (N.B. this includes any numerical values that were used to generate graphs, histograms etc.). For an example see here: http://www.plosbiology.org/article/info%3Adoi%2F10.1371%2Fjournal.pbio.1001908#s5

*Blot and Gel Data Policy*

We require the original, uncropped and minimally adjusted images supporting all blot and gel results reported in an article's figures or Supporting Information files. We will require these files before a manuscript can be accepted so please prepare them now, if you have not already uploaded them. Please carefully read our guidelines for how to prepare and upload this data: https://journals.plos.org/plosbiology/s/figures#loc-blot-and-gel-reporting-requirements

*Protocols deposition*

To enhance the reproducibility of your results, we recommend that if applicable you deposit your laboratory protocols in protocols.io, where a protocol can be assigned its own identifier (DOI) such that it can be cited independently in the future. Additionally, PLOS ONE offers an option for publishing peer-reviewed Lab Protocol articles, which describe protocols hosted on protocols.io. Read more information on sharing protocols at https://plos.org/protocols?utm_medium=editorial-email&utm_source=authorletters&utm_campaign=protocols

Thank you again for your submission to our journal. We hope that our editorial process has been constructive thus far, and we welcome your feedback at any time. Please don't hesitate to contact us if you have any questions or comments.

Sincerely,

Roli Roberts

Roland Roberts, PhD

Senior Editor

PLOS Biology

rroberts@plos.org

----------------------------------------------------------------

REVIEWERS' COMMENTS:

Reviewer #1:

I want to commend the authors on a much improved manuscript. The focus and writing are now much clearer to my eyes and the manuscript is now much more effective at addressing the more limited set of goals. As currently framed, the manuscript now focuses specifically on how immigration can affect evolutionary changes in addition to in situ changes in that trait in response to natural selection. As the authors correctly mention in their manuscript, most long-term studies of individually marked animals that are used to perform studies such as this are 'by design' relatively isolated. This means that immigration is minimized with respect to other evolutionary processes, but this does not mean that it is completely eliminated. This study does a very nice job of showing the importance of this immigration to evolutionary change and the degree to which this might cloud our ability to detect evolutionary responses to natural selection within the population (i.e. apparent paradox of stasis).

There is one more technical issue that I wanted to discuss and there are some ways in which I think the authors could make their case more strongly. First, I think we all agree that by definition the mean breeding value of base animals in the pedigree is defined to be zero. Base animals are those at the start of the study and immigrants with unknown parents. I was, therefore, confused by the sentence on lines 247-249. I am unclear on how the genetic merit of unknown-origin immigrants could change since their mean breeding values should always be zero. This made me worry I was misunderstanding something here. Beyond this specific point, I think there is a more general point that the authors could make more strongly. What I think is unique and very valuable about this study is that much of the immigration was not by animals of unknown origin but instead was of animals of known origin from another population. I think this is very different from most comparable studies in which almost all immigration will be by animals of unknown origin. This the case of other studies this animals are assumed to have a mean breeding value of zero and they will simply dilute any population change in mean breeding value. In contrast, the breeding values of immigrants in this study can be substantially non-zero because they are often not defined to be base animals. This leads to the very interesting plot (Fig 5) showing how very non-zero breeding values in immigrants could accentuate or oppose in situ evolution. I know this is the authors' main point but I think they could make their case stronger for the way in which their study has revealed a process that is otherwise very difficult to uncover. Mean breeding values of unknown immigrants is often very likely different from zero but when we have no other information we must assume the mean is zero. This study shows how we might be quite misled in this necessary assumption. I would suggest that the authors try and make this case more strongly since I think this is one of the most impactful stories of this study.

My other general comment is that I don't think the authors have fully discussed the degree to which immigration explains the observed mismatch between expected and observed responses to selection. To be transparent the degree of uncertainty around the metric for the degree to which observed change is below expected change is so large that the change observed with immigrants is not statistically different from the in situ change. I will be honest and say that i am not too hung up on these p-values and uncertainty around these metrics. I appreciate the authors' quantitative approach. Others might be more concerned about the P-values so I would suggest the authors be more transparent about how this high uncertainty might be interpreted. Quantitatively though observed changes were only 37% of expected (if I understand the metric correctly), whereas after accounting for immigration the observed in situ changes were 75% of expected. Quantitatively this story is interesting to me despite the large uncertainty in these metrics. Immigration accounted for a large component of the 'stasis', but I think it is worth also discussing how even after accounting for immigration there remained some degree of stasis. I think this needs to be discussed at the end of the paper. Some stasis can be explained by immigration in this system but not all stasis.

Overall, I think there are some important new lessons to be learned from this study. The authors have a unique study system that has allowed them to use clever and innovative approaches to reveal some interesting patterns that could not have been studied in other systems. These patterns have the potential to explain some (but not all) of the more general pattern of observed evolutionary changes being weaker than expected based on quantitative genetic predictions. Together, I think this makes for a compelling and valuable study.

Some Specific Comments:

- text around figures 2 and 3 suggests that there is no sampling error around the estimate of S. I suppose this is true in the calculation but there is still uncertainty in this measure that can be calculated through resampling techniques. That said, I'm not connived that ignoring uncertainty in S is detrimental to the overall story.

- please be clear in the figure legends about exactly what years were analyzed. I think the authors excluded the experimental period (based on the response letter) but this wasn't clear to me in the text.

- Fig 3 essentially depicts a realized heritability, but I don't think it is described in this way in the legend or the text.

- Line 157. The Paradox of stasis was reduced (not significantly because the uncertainty around the 0.367 and the 0.749 are very large) but not eliminated. This needs to be more fully discussed. I don't think the degree to which immigration can explain stasis in this system is currently discussed.

- Side note regarding above - I think the magnitude of the confidence intervals is so large that it is not informative to measure these ratios (delta a/bo) to three decimal places. Perhaps only one is sufficient. Evolutionary change goes from 0.4 of expected to 0.7 of expected.

Reviewer #2:

[identifies himself as Joel Pick]

This revision of the paper has addressed many of my previous comments. Overall I really like the study. I think it has a lot of value, and many of the analyses are very insightful. I think the results are well suited to PLOS Biology.

However, I found the manuscript quite hardwork to follow in places. I would suggest that an easier way for a reader would be to clearly set out at the end of the introduction what hypotheses are being tested/ what data is being used to test them, and then include subheading in the results to help guide a reader through, preferably matched to the methods. The intro focuses on immigration, but then the results goes through a performance of setting up the return after selection ends should be due to natural selection, and poses a new paradox. However, as a reader this seems unnecessary, as immigration has already been introduced as a solution. The results also move back and forth between the selection experiment and overall selection/response to selection in the population as a whole. I find this a bit difficult to deal with as a reader. Again feel that a bit of reorganisation, better signposting in the introduction of what is to come, and some subheadings would really help.

Main comment

I still am struggling to put this study into the context of the paradox of stasis/general problem of misprediction.

The paradox of stasis, in my opinion (and I think is supported by commonly cited papers in this area e.g. Merila et al 2001, Pujol et al 2018), is typically used to refer to traits under strong persistent direction selection, i.e. the same selection pressure (give or take) over time, and across populations, and often species (an example of this being juvenile body size). In the response, it is stated that the paradox of stasis is more about unmet expectations - whilst I agree that this is a more general problem, into which the paradox of stasis fits, I think the paradox of stasis itself is more specific than this - after all it is a paradox of *stasis*, i.e. why we find stasis when we don't expect to, due to strong persistent directional selection. This is a matter of framing, and ultimately up to the authors, but I suspect other readers may have a similar response.

However, my issue is a broader point about misprediction (or however we choose to define the paradox of stasis). I think the second paragraph of the introduction gets to the crux of my problem. I can see that for very isolated populations, like the ones cited, there may be very local selection pressures, and the few immigrants from other populations will erode selection response, if those other populations face different selection pressures. In this case, we may study that isolated population, and then wonder why there is no response to the selection. My question is, how common is this? I agree that generally in wild quant gen studies, we do often ignore gene flow. However, for immigration to erode selection response, there needs to be clear differences in the selection pressures in populations connected by immigration. Yet, there seems reasonable evidence that strong selection is often temporally and spatially consistent, especially in direction (Morrissey and Hadfield 2012 https://doi.org/10.1111/j.1558-5646.2011.01444.x, Siepielski et al. 2013 https://doi.org/10.1111/ele.12174), suggesting pockets of strong and persistently different selection within a metapopulation would be unlikely, especially in traits that are emblematic of the paradox of stasis.

In this population, clutch size appears not to be under persistent directional selection, so the paradox of stasis does not refer to the phenotype as a whole in this population. Rather selection appears to fluctuate around 0. Although it is argued that you do not need to consider this selection with uncertainty (discussed more in comments below), the reality is that differences in fitness among individuals, especially in small populations (as here, especially in the west), will largely reflect stochastic variation, and so there will be a certain amount of apparent selection by chance (i.e. drift), fluctuating around zero. There is no apparent test as to whether this is more variation in selection than would be expected by sampling variance alone. This apparent selection and the subsequent genetic change are matched up and used as a test for stasis. However, in the years outside of the selection experiment, it is difficult to see that this is a test of stasis, given that there is little evidence that this is selection rather than drift. In terms of general misprediction, the initial comparison of selection and response includes immigrants. Given the precise definition of selection, this seems misleading to present first, and indeed when the immigrants are removed the prediction matches much better.

The study also focuses on an experiment inducing a short period of intense selection. However, this selection is geographically isolated (indeed being in opposite direction in two neighbouring populations), in populations with high immigration rates. During the period of selection, there seems to be a clear response to that selection, indicating that while selection is acting, immigration does not prevent this response, i.e. immigration does not drive stasis, or misprediction? When it stops, the response is then swamped by immigration, from surrounding areas. If in an artificial selection experiment in the lab, I selected for clutch size, and then stopped selection and bred the lines with an unselected control line afterwards, it would not seem a surprise that they return to the control phenotype. Again, I find this very hard to reconcile with the paradox of stasis.Before the experiment, there was no persistent directional selection, and there was no change. During the experiment, there was strong directional selection and there was a change. After the experiment, when there was no persistent directional selection, and high immigration from surrounding unselected, or differentially selected areas, the clutch size then returned to the previous state. In each case I think that this is what we would predict?

I don't say this to try and diminish the ambition or importance of this study. I think examining what is happening in a population with this level of detail is extremely illuminating, especially in the context of this large scale quant gen experiment, which is very rare. I think the decomposition of what is driving change in the system, in terms of immigration and in situ change is very elegant, and overall this is a very valuable study. However, the introduction sets it up as pitching for a mechanism that would provide a general solution to the paradox of stasis. Whilst I love a good pun, the title even hints that we have solved the paradox. I don't think that this study can provide a general solution to this paradox, without providing compelling evidence that this phenomenon (very strong, but temporary and localised selection, in a background of no selection) is common, and represents a large amount of what we see when referring to the paradox of stasis.

I would therefore again encourage the authors to consider a slight reframing, and particularly in the context of the 'paradox lost' part of the title. Although I see this as an important and interesting study, I do not think it takes us nearer to a resolution of the paradox of stasis, especially as there is likely no global solution.

---

Specific comments

1. L39 'the 'paradox of stasis' has withstood attempts at general resolution and become emblematic of our struggle to understand contemporary adaptive evolution in natural populations.'

I agree with the second half of this sentence, but do we expect a general resolution? In the well cited concept papers on this topic (e.g. Merila et al. 2001, Pujol et al. 2018), there has been no suggestion of a global solution - indeed they suggest many, not mutually exclusive reasons. Also some work has found resolution of the problem within single populations (e.g. Mittell et al 2025 https://doi.org/10.1073/pnas.2513969122).

2. L42 'the paradox of stasis is seldom subject to objective assessment; rather, it is generally evoked a posteriori.'

I don't think I quite get this. I would say that a lot of studies in recent years have focussed on why there is no change, for example, looking at environmental covariances, missing fraction, phenotypic skew, social interactions and immigration. This makes it sounds as though lots of studies are targeted at finding a change, and then when they don't find one, throw out the paradox of stasis as a general solution. This is not my experience of the literature. Perhaps I have missed the point the authors are trying to make, but then it should perhaps be more obvious!

3. L47 'Immigrants are likely to be genetically distinct from their host population'

This seems a bold claim, and I think this needs some context, and perhaps some evidence. The two references given are of isolated island populations, with low migration rates, that are more likely to be genetically distinct. In the context of many studies looking at the paradox of stasis, (including many mainland populations), it seems unlikely this is generally the case. It seems important to point this out, when discussing a 'general resolution' to this problem.

4. L 64. Once again, it would be helpful to frame this study in the light of the previous work done on this population, in which differential immigration from the mainland was found to be a major driver of the differences between populations.

5. Figure 2 - A zero line would be helpful for the selection plot (b). I would also suggest adding errors to these estimates! As I discuss in a comment below, I understand the argument that the selection differential used in the regression of selection and response - the in situ selection here is known from the breeders and recruits. However, in small populations, there is considerable drift, driven by stochastic associations between a phenotype and fitness. The uncertainty on these estimates therefore gives an idea of the level of uncertainty there is in whether and how often these values differ from 0, and so how much of this apparent selection might represent neutral processes.

6. L104'This evolutionary return toward the pre-experimental state is consistent with the adaptationist expectation that the experimental evolutionary perturbation (which was driven by strong directional artificial selection) is opposed by natural selection, with local adaptation returning the difference between the two populations toward the status quo ante.'

I am a little confused by this statement. The pre-experiment state was a difference between the two populations. After the selection, the two populations are the same. I would not interpret this as things returning to the status quo, as they are clearly different post selection. If local adaptation was acting before and after, then they should also be different after. As per a comment above, previous studies of this system have also shown that this difference appeared to be quite substantially driven by immigration, so I think this phrasing is a little misleading.

7. L 107 'However, in contrast to this scenario, annual selection on clutch size after the experiment was negligible (one-sample t-tests: West: 7 = −0.53, = 0.612, East: 7 = 1.12, = 0.299)'

It's not clear here what this test is - is it directional testing against 0? It is also not clear in the figure or text what these selection estimates are - selection differentials?

8. L 112 'which was characterised by evolutionary stasis of clutch size in both West and East'

Although it is technically correct, I think this is a confusing use of this term here given the pitch of the manuscript - it made me think of traits not evolving when there is selection, when in this case there is no evolution and no selection.

L 116 ' and might therefore might be argued to represent a corresponding 'paradox of change'

Once again - I see no paradox here. If you were doing this study in a completely isolated population, like with artificial selection in the lab, then the return to pre-experiment would make sense in terms of opposing natural selection. However, doing this experiment in an open population, immigration would be expected to swamp the response after selection stops. So this 'paradox of change' seems like setting up a strawman. This is possibly some narrative device to slowly build up the bigger picture, but given the previous research on this system, I feel this is unwarranted. I think this can much more simply be presented as 'in an open population, we might expect that reversal of evolutionary response after a brief period of selection occurs either due to opposing natural selection or to immigration, and we test between them.'

This framing also clashes with the introduction, where in the second paragraph, immigration is introduced as a solution to the paradox of stasis, but then there are 3 paragraphs of the results ignoring immigration and focussing on selection. I would suggest a slight re-ordering, so that after considering what happened after the period of artificial selection in terms of natural selection, it is then considered in terms of immigration. As it stands, it then moves to generally looking at general selection on clutch size across the whole period.

9. L 125 ' S (i.e. the covariance between clutch size and annual fitness) was an unreliable predictor of interannual genetic change in the breeding population'

This seems like a correlation. As articulated in my last review, both genetic change (and possibly selection) are measured with considerable error. Without taking this into account, the relationship will of course be weak. Measurement error attenuates relationships. One solution is to model this in a meta-analytic framework.

10. In the previous review, I criticised the lack of error on the selection differential. I can see that the argument is that because all individuals and their reproduction is known in the population, then the within generation change is known exactly. This is similar to when this is applied in an animal breeding context. However, in the context of animal breeding, we also know all the individuals at the next timepoint come from the individuals present (i.e. there is no immigration). I think then comparing this with all the breeding values in the next timepoint is a bit disingenuous. First, some of the new individuals are immigrants, and second there will have been emigrant offspring, so the actual fitness of the breeders is not actually known - only their local fitness (hence selection is actually estimated with error!). In this case, I would argue that you can only apply this to local recruits. This is done in a second step, but as stated above, I think this is part of setting up a strawman argument. I can see that the comparison of the two is useful to demonstrate the impact of immigration to this local change, but this should be presented in a second step.

11. The regression of S on R gives the estimated heritability. As said later in the manuscript, selection should be halved (or slope doubled). So the predicted heritability from this method is 0.066 [−0.134, 0.278], including immigrants, and 0.156 [-0.068, 0.378] without. I can't see this being compared against the estimate of h2 presented in supplements 0.226 [0.172, 0.284], which would seem logical to me.

12. L130 It is correct that the selection could be biased by an environmental covariance, but this then represents a whole other hypothesis for the paradox of stasis, which hasn't been introduced sufficiently. To be perfectly honest, it seems that much of this might be more directly tested with a multivariate mixed model of fitness and clutch size than the roundabout way presented.

13. L135 - 141. I don't think I really follow this? Is this the same as the year specific heritability calculation in the methods? If so, I would highly recommend labelling it as such, rather than a 'conversion rate'. It's also worth noting there that this assumes that Va is constant, as individuals have a constant breeding value, and so the year specific heritability is being generated by allowing Vp to vary rather than Va.

14. L157 As above, I didn't really understand these statistics, and could link them clearly to a part of the methods.

15. L159 'perhaps owing to prevailing expectations that the evolutionary effect of gene flow is ignorable [33]'

I think this is a misrepresentation. I presume that this refers to part of the questionnaire where researchers were asked 'How important are each of these factors in limiting adaptive responses to climate change', and gene flow was the ranked lowest. Climate change works at much larger spatial scales to what is being described here, and so when we are considering response to climate change, any shift in selection pressure would likely be persistent across populations connected by immigration. Therefore I don't think this can be used to state that researchers generally think that 'the evolutionary effect of gene flow is ignorable'.

16. Figure 4 - Given that this is simulated data and not results, it seems like a figure more suited to the introduction, as a demonstration of how immigration can affect observed change. I dont think it adds much to a results section.

17. L 212 'This describes the genetic impact of local adaptation'

And drift!!

18. L 219 'The latter may be linked to long-term environmental changes in the West creating a post-experimental selection regime that differs from that experienced by earlier generations.'

But is not reflected in the in selection shown in figure 2b

19. L 229 'conceals negative selection on clutch size at a genetic level'

Selection is phenotypic, and describes a causal relationship between a phenotype and fitness. There are several forces that may make it seem as though selection is stronger or weaker, e.g. an environmental covariance between a trait and fitness can make it seem as though there is selection when there isn't (as mentioned), or soft selection attenuating selection when hard selection is assumed (although as this is within year selection this wouldn't be an issue here). If there is a genetic relationship but not a phenotypic one, I don't think this provides any evidence for selection. It seems more plausible that there is a genetic correlation with another trait under selection, or this is drift.

20. L269 'While adaptive responses to observed selection on clutch size were directionally consistent with adaptationist expectations, they were, overall, smaller than predicted via application of Robertson's secondary theorem'

I don't think there is any evidence of adaptive response outside of the selection experiment. This is also the first (and only?) mention of the secondary theorem, so I think this either needs to be explained or dropped. I also don't quite see how it comes in here, as the genetic covariance between clutch size and fitness is not estimated (although doing so may simplify some of what is presented).

21. I haven't reviewed more of the discussion, as I can't see any convincing evidence that there is any natural selection acting on clutch size at any point, but the discussion is written completely in the context of the paradox of stasis.

22. L379 'quantitative genetic statistics are calculated for each sampled iteration and the distributions across all sampled iterations taken to represent posterior distributions.'

I think this needs to be clearer what this refers to. For example, in a comment above, I have written about the correlations between R and S needing to account for error. Now I am not sure from this sentence whether this analysis is included in this, as it is not a quantitative genetic statistic.

23. L 391 I'm not sure where this is presented! Whilst this does take into account one source of error, it assumes that the mean in each year within an iteration is also estimated without error, and so some years the sample sizes are not that big.. Each year will also be weighed the same, even though the population sizes in each year may be quite different from each other. I can't fully workout if this is an issue, but a weighted regression for each interaction might help?

24. L 416 a statistically significant quadratic is not evidence of either stabilising or disruptive selection, rather that there is just non-linearity. There would have to be evidence that the optimum was within the range of the data. I would suggest not referring to stabilising or disruptive selection and rather refer to positive and negative quadratic selection.

25. L 426 'The breeder's equation predicts the phenotypic change from one generation to the next [5]. We cannot use the breeder's equation to predict the genetic change from one year to the next because the breeding population in the next year is partially made up of survivors from the previous year, whose phenotype will be more similar to their phenotype last year than expected based on the heritability of the trait (i.e. due to permanent environment effects). '

This is true for phenotypic change, but not genetic change - the breeders equation with 'annual genetic success' will correctly predict genetic change, as the genetic change doesn't not include the permanent environment effects. If fitness is half fecundity(f) plus survival(s) then:

R = h2S

R = h2 cov(f/2+s,z)

R = h2 cov(f/2,z) +h2 cov(s,z)

In terms of the phenotypic response, this does not work, and the surviving individuals will either have the same phenotype in the case of fixed traits, or a phenotype dictated by the traits repeatability across years, and so scaling the survival component by heritability doesn't work. For the phenotypic change you would want

Delta_p = h2 cov(f/2,z) + cov(s,z)

However it does work for the genetic change, as the heritability also represents the relationship between genotype and phenotype, and so phenotypic selection via survival is scaled by the h2 to get the genetic change:

Delta_a = h2S

Delta_a = h2 cov(f/2+s,z)

Delta_a = h2 cov(f/2,z) +h2 cov(s,z)

As is pointed out, selection would also be halved, as it is only on females.

26. L 438 there seems to be a mix of notation with a_bar and mu_a. The mu_a notation has loads of nested subscriptions and becomes hard to follow. Perhaps adopting the a_bar notation throughout would be easier to follow? Given that these are realised values that you are calculating, and not theoretical parameters, I think the a_bar notation is more suitable. Also is there a reason to use x for time, instead of th more traditional t? I think this would also help digest the many subscripts

27. L 445 is there a couple of 'female breeder's missing from the first covariance?

28. L446 - I don't understand this - there is an unexplained leap. Unless it is meant to be sigma^2_{a_{female breeder_{x}}} / sigma^2_{*z*_{female breeder_{x}}}, and not sigma^2_{*w*_{female breeder_{x}}}? I think this might emphasise how difficult it is to keep track of this notation.

29. L449-L458 This is a nice mathematical demonstration of this, but it is known that heritability is given by the regression of a on z, so it could just be stated, given that there is a fair bit of complexity and notation to follow. It also isn't well linked back to the results, or its relevance made very clear. As noted above, this calculation of year specific heritability assumes a constant va, and so variation in this only comes from changing Vp.

Joel Pick

Reviewer #3:

The authors have thoroughly and thoughtfully addressed all my previous comments (Reviewer 3) and sufficiently clarified and revised the manuscript. After a careful reading of the manuscript, I do not have any criticisms or comments and think this is an excellent study!

# Author comment: Author response to Decision Letter 3

**DOI:** 10.1371/journal.pbio.3004004.r005

10 Aug 2026

Attachment Submitted filename: Response to reviewers comments.docx

# Editor report: Decision Letter 3

**DOI:** 10.1371/journal.pbio.3004004.r006

**Authors:** Roland Roberts

1 Sep 2026

Dear Dr Evans,

Thank you for your patience while we considered your revised manuscript "Paradox lost? Gene flow drives periods of both evolutionary stasis and change in a wild bird population" for publication as a Research Article at PLOS Biology. This revised version of your manuscript has been evaluated by the PLOS Biology editors and the Academic Editor.

Based on our Academic Editor's assessment of your revision, we are likely to accept this manuscript for publication, provided you satisfactorily address the following data and other policy-related requests.

IMPORTANT - please attend to the following:

a) We're not sure that the initial "paradox lost?", while nifty, contributes much to the Title; in addition the includion of punctuation in titles runs the risk of truncation by some aggregators. We therefore suggest that you remove it, changing your Title to simply "Gene flow drives periods of both evolutionary stasis and change in a wild bird population"

b) Please address my Data Policy requests below; specifically, we need you to supply the numerical values underlying Figs 2ABC, 3AB, 4, 5AB, 6ABC, S1, S2, S3AB, S4, S5, S6, S7, S8, S9AB, S10AB, S11, either as a supplementary data file or as a permanent DOI’d deposition. I note that you already have an associated Zenodo deposition (https://zenodo.org/records/21861701). Please could you confirm whether the data and code in this deposition are sufficient to recreate the Figures?

c) Please cite the location of the data clearly in all relevant main and supplementary Figure legends, e.g. “The data underlying this Figure can be found in S1 Data” or “The data and code needed to generate this Figure can be found in https://zenodo.org/records/21861701".

d) I note that you mention the reviewers (“as well as J. Pick and two anonymous reviewers”) in the Acknowledgements. While we appreciate the sentiment, this is against PLOS policy, so please could you remove this?

e) Please make any custom code available, either as a supplementary file or as part of your data deposition.

As you address these items, please take this last chance to review your reference list to ensure that it is complete and correct. If you have cited papers that have been retracted, please include the rationale for doing so in the manuscript text, or remove these references and replace them with relevant current references. Any changes to the reference list should be mentioned in the cover letter that accompanies your revised manuscript.

In addition to these revisions, you may need to complete some formatting changes, which you will receive in a follow up email. A member of our team will be in touch with a set of requests shortly. If you do not receive a separate email within a few days, please assume that checks have been completed, and no additional changes are required.

We expect to receive your revised manuscript within two weeks.

To submit your revision, please go to https://www.editorialmanager.com/pbiology/ and log in as an Author. Click the link labelled 'Submissions Needing Revision' to find your submission record. Your revised submission must include the following:

- a cover letter that should detail your responses to any editorial requests, if applicable, and whether changes have been made to the reference list

- a Response to Reviewers file that provides a detailed response to the reviewers' comments (if applicable, if not applicable please do not delete your existing 'Response to Reviewers' file.)

- a track-changes file indicating any changes that you have made to the manuscript.

NOTE: If Supporting Information files are included with your article, note that these are not copyedited and will be published as they are submitted. Please ensure that these files are legible and of high quality (at least 300 dpi) in an easily accessible file format. For this reason, please be aware that any references listed in an SI file will not be indexed. For more information, see our Supporting Information guidelines:

https://journals.plos.org/plosbiology/s/supporting-information

*Published Peer Review History*

Please note that you may have the opportunity to make the peer review history publicly available. The record will include editor decision letters (with reviews) and your responses to reviewer comments. If eligible, we will contact you to opt in or out. Please see here for more details:

https://plos.org/published-peer-review-history/

*Press*

Should you, your institution's press office or the journal office choose to press release your paper, please ensure you have opted out of Early Article Posting on the submission form. We ask that you notify us as soon as possible if you or your institution is planning to press release the article.

*Protocols deposition*

To enhance the reproducibility of your results, we recommend that if applicable you deposit your laboratory protocols in protocols.io, where a protocol can be assigned its own identifier (DOI) such that it can be cited independently in the future. Additionally, PLOS ONE offers an option for publishing peer-reviewed Lab Protocol articles, which describe protocols hosted on protocols.io. Read more information on sharing protocols at https://plos.org/protocols?utm_medium=editorial-email&utm_source=authorletters&utm_campaign=protocols

Please do not hesitate to contact me should you have any questions.

Sincerely,

Roli

Roland Roberts, PhD

Senior Editor

rroberts@plos.org

PLOS Biology

------------------------------------------------------------------------

DATA POLICY:

You may be aware of the PLOS Data Policy, which requires that all data be made available without restriction: http://journals.plos.org/plosbiology/s/data-availability. For more information, please also see this editorial: http://dx.doi.org/10.1371/journal.pbio.1001797

Note that we do not require all raw data. Rather, we ask that all individual quantitative observations that underlie the data summarized in the figures and results of your paper be made available in one of the following forms:

1) Supplementary files (e.g., excel). Please ensure that all data files are uploaded as 'Supporting Information' and are invariably referred to (in the manuscript, figure legends, and the Description field when uploading your files) using the following format verbatim: S1 Data, S2 Data, etc. Multiple panels of a single or even several figures can be included as multiple sheets in one excel file that is saved using exactly the following convention: S1_Data.xlsx (using an underscore).

2) Deposition in a publicly available repository. Please also provide the accession code or a reviewer link so that we may view your data before publication.

Regardless of the method selected, please ensure that you provide the individual numerical values that underlie the summary data displayed in the following figure panels as they are essential for readers to assess your analysis and to reproduce it: Figs 2ABC, 3AB, 4, 5AB, 6ABC, S1, S2, S3AB, S4, S5, S6, S7, S8, S9AB, S10AB, S11. NOTE: the numerical data provided should include all replicates AND the way in which the plotted mean and errors were derived (it should not present only the mean/average values).

IMPORTANT: Please also ensure that figure legends in your manuscript include information on where the underlying data can be found, and ensure your supplemental data file/s has a legend.

Please ensure that your Data Statement in the submission system accurately describes where your data can be found.

------------------------------------------------------------------------

CODE POLICY

Per journal policy, if you have generated any custom code during the course of this investigation, please make it available without restrictions. Please ensure that the code is sufficiently well documented and reusable, and that your Data Statement in the Editorial Manager submission system accurately describes where your code can be found. More information on our Code Policy, what and how to share can be found here: https://journals.plos.org/plosbiology/s/code-availability

Please note that we cannot accept sole deposition of code in GitHub, as this could be changed after publication. However, you can archive this version of your publicly available GitHub code to Zenodo. Once you do this, it will generate a DOI number, which you will need to provide in the Data Accessibility Statement (you are welcome to also provide the GitHub access information). See the process for doing this here: https://docs.github.com/en/repositories/archiving-a-github-repository/referencing-and-citing-content

------------------------------------------------------------------------

DATA NOT SHOWN?

- Please note that per journal policy, we do not allow the mention of "data not shown", "personal communication", "manuscript in preparation" or other references to data that is not publicly available or contained within this manuscript. Please either remove mention of these data or provide figures presenting the results and the data underlying the figure(s).

------------------------------------------------------------------------

# Editor report: Decision Letter 4

**DOI:** 10.1371/journal.pbio.3004004.r007

**Authors:** Roland Roberts

3 Sep 2026

Dear Simon,

Thank you for the submission of your revised Research Article "Gene flow drives periods of both evolutionary stasis and change in a wild bird population" for publication in PLOS Biology. On behalf of my colleagues and the Academic Editor, Michael Jennions, I'm pleased to say that we can in principle accept your manuscript for publication, provided you address any remaining formatting and reporting issues. These will be detailed in an email you should receive within 2-3 business days from our colleagues in the journal operations team; no action is required from you until then. Please note that we will not be able to formally accept your manuscript and schedule it for publication until you have completed any requested changes.

Please take a minute to log into Editorial Manager at http://www.editorialmanager.com/pbiology/, click the "Update My Information" link at the top of the page, and update your user information to ensure an efficient production process.

PRESS: We frequently collaborate with press offices. If your institution or institutions have a press office, please notify them about your upcoming paper at this point, to enable them to help maximise its impact. If the press office is planning to promote your findings, we would be grateful if they could coordinate with biologypress@plos.org. If you have previously opted in to the early version process, we ask that you notify us immediately of any press plans so that we may opt out on your behalf.

We also ask that you take this opportunity to read our Embargo Policy regarding the discussion, promotion and media coverage of work that is yet to be published by PLOS. As your manuscript is not yet published, it is bound by the conditions of our Embargo Policy. Please be aware that this policy is in place both to ensure that any press coverage of your article is fully substantiated and to provide a direct link between such coverage and the published work. For full details of our Embargo Policy, please visit http://www.plos.org/about/media-inquiries/embargo-policy/.

Thank you again for choosing PLOS Biology for publication and supporting Open Access publishing. We look forward to publishing your study.

Sincerely,

Roli

Roland G Roberts, PhD, PhD

Senior Editor

PLOS Biology

rroberts@plos.org
